# Regional-Scale In-Depth Analysis of Soil Fungal Diversity Reveals Strong pH and Plant Species Effects in Northern Europe

**DOI:** 10.3389/fmicb.2020.01953

**Published:** 2020-09-04

**Authors:** Leho Tedersoo, Sten Anslan, Mohammad Bahram, Rein Drenkhan, Karin Pritsch, Franz Buegger, Allar Padari, Niloufar Hagh-Doust, Vladimir Mikryukov, Daniyal Gohar, Rasekh Amiri, Indrek Hiiesalu, Reimo Lutter, Raul Rosenvald, Elisabeth Rähn, Kalev Adamson, Tiia Drenkhan, Hardi Tullus, Katrin Jürimaa, Ivar Sibul, Eveli Otsing, Sergei Põlme, Marek Metslaid, Kaire Loit, Ahto Agan, Rasmus Puusepp, Inge Varik, Urmas Kõljalg, Kessy Abarenkov

**Affiliations:** ^1^Institute of Ecology and Earth Sciences, University of Tartu, Tartu, Estonia; ^2^Zoological Institute, Technische Universität Braunschweig, Brunswick, Germany; ^3^Department of Ecology, Swedish University of Agricultural Sciences, Uppsala, Sweden; ^4^Institute of Forestry and Rural Engineering, Estonian University of Life Sciences, Tartu, Estonia; ^5^Helmholtz Zentrum München – Deutsches Forschungszentrum für Gesundheit und Umwelt (GmbH), Neuherberg, Germany; ^6^Chair of Forest Management Planning and Wood Processing Technologies, Institute of Plant and Animal Ecology, Ural Branch, Russian Academy of Sciences, Yekaterinburg, Russia; ^7^Forest Health and Biodiversity, Natural Resources Institute Finland (Luke), Helsinki, Finland; ^8^Chair of Plant Health, Estonian University of Life Sciences, Tartu, Estonia; ^9^Natural History Museum and Botanical Garden, University of Tartu, Tartu, Estonia

**Keywords:** island biogeography, community ecology, niche analysis, forest management, anthropogenic impact, ectomycorrhizal fungi, PacBio SMRT sequencing

## Abstract

Soil microbiome has a pivotal role in ecosystem functioning, yet little is known about its build-up from local to regional scales. In a multi-year regional-scale survey involving 1251 plots and long-read third-generation sequencing, we found that soil pH has the strongest effect on the diversity of fungi and its multiple taxonomic and functional groups. The pH effects were typically unimodal, usually both direct and indirect through tree species, soil nutrients or mold abundance. Individual tree species, particularly *Pinus sylvestris*, *Picea abies*, and *Populus x wettsteinii*, and overall ectomycorrhizal plant proportion had relatively stronger effects on the diversity of biotrophic fungi than saprotrophic fungi. We found strong temporal sampling and investigator biases for the abundance of molds, but generally all spatial, temporal and microclimatic effects were weak. Richness of fungi and several functional groups was highest in woodlands and around ruins of buildings but lowest in bogs, with marked group-specific trends. In contrast to our expectations, diversity of soil fungi tended to be higher in forest island habitats potentially due to the edge effect, but fungal richness declined with island distance and in response to forest fragmentation. Virgin forests supported somewhat higher fungal diversity than old non-pristine forests, but there were no differences in richness between natural and anthropogenic habitats such as parks and coppiced gardens. Diversity of most fungal groups suffered from management of seminatural woodlands and parks and thinning of forests, but especially for forests the results depended on fungal group and time since partial harvesting. We conclude that the positive effects of tree diversity on overall fungal richness represent a combined niche effect of soil properties and intimate associations.

## Introduction

Soil microorganisms such as bacteria, archaea and fungi play integral roles in soil ecosystem functioning. In particular, fungi act as principal decomposers of organic material, key root mycorrhizal symbionts of plants as well as harmful pathogens in forest, grassland and agricultural habitats. It has been argued that the diversity of soil organisms is of particular importance for ecosystem services and resilience to disturbances, with a potential to ameliorate stress caused by global change (Langenheder et al., 2010; Bardgett and van der Putten, 2014). Many of these soil organisms are vulnerable to shifts in land use, changing climate and ecosystem management (Van der Putten, 2013; George et al., 2019; Makiola et al., 2019; Sterkenburg et al., 2019); therefore, some of the most conspicuous rare species are protected (Dahlberg et al., 2010). However, fungal species that have large fruitbodies represent only a tip of the iceberg in the enormous diversity of soil mycobiome, much of which is cryptic and previously undocumented. Advances in the so-called environmental DNA (eDNA) analyses have enormously improved our understanding about the diversity and distribution of small soil organisms including fungi (Taberlet et al., 2018; Nilsson et al., 2019).

Virtually all members of the soil biota exhibit distinct distribution patterns from microscale to global scale in relation to soil parameters, vegetation and climate. These environmental effects are also linked to spatiotemporal patterns caused by seasonal climate variability as well as dispersal limitation at the landscape and continental scales (Bahram et al., 2015; Cavicchioli et al., 2019). While several global efforts have provided rough information about the distribution of soil microbes on the Earth, where climate typically drives large-scale patterns (Tedersoo et al., 2014; Bahram et al., 2018; Delgado-Baquerizo et al., 2018), we still lack comprehensive studies from landscape to regional scales that are more relevant to understanding the immediate effects of vegetation, land use and edaphic properties on soil organisms (but see Karimi et al., 2018; George et al., 2019). The regional geographic scale is important to consider, because this is typically little affected by historical dispersal limitation and climate (except montane systems), especially the covariation of climate with vegetation and soil properties – all inherent to larger geographic scales (Lomolino et al., 2006). Multiple highly focused studies have demonstrated the effects of land use, dominant plant species, soil properties, pollution and management by humans on soil microorganisms (Rousk et al., 2010a; Hartmann et al., 2012; Urbanova et al., 2015; Creamer et al., 2016; Sterkenburg et al., 2019). However, these studies do not enable us to understand the relative importance of these factors because of their limited ecological scope.

**FIGURE 1:**
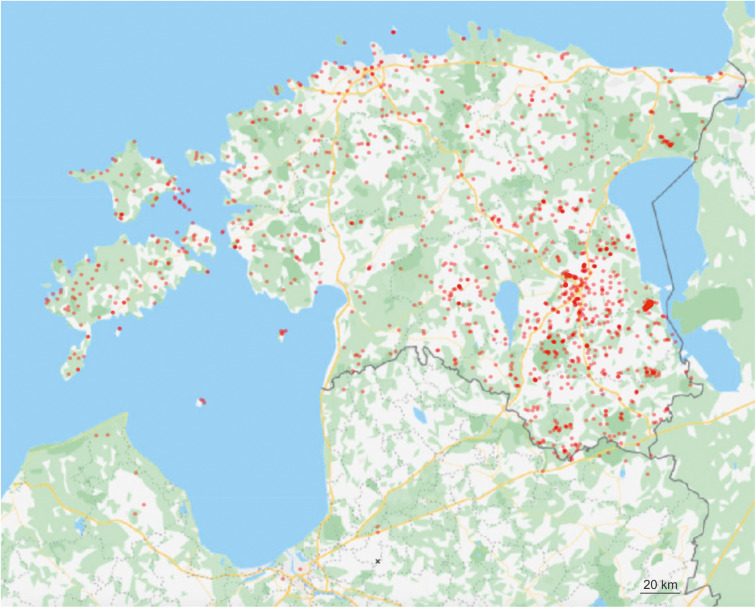
Placement of sampling plots in the Northern Baltic region.

Using third-generation PacBio sequencing and 1251 standard composite samples, we provide a detailed view on the biotic and abiotic factors underlying the distribution of soil fungi, across different habitat and vegetation types, ecosystem management regimes and edaphic gradients in the northern Baltic region (Figure 1). Our main purpose was to evaluate differences in the response of taxonomic and functional groups of fungi to various abiotic and biotic variables by explicitly accounting for spatial and temporal autocorrelation, microclimatic differences and technical biases in a multi-year sampling effort. We tested the following hypotheses: (1) diversity of biotrophic groups responds strongest to dominant vegetation, whereas free-living groups are more affected by abiotic variables; (2) diversity of soil fungi is relatively lower in island and fragmented forest ecosystems, especially in small and distant habitats following the island biogeography theory; (3) anthropogenic ecosystems harbor lower mycobiome diversity than natural and particularly pristine habitats; (4) management of forests, seminatural woodlands and parks reduces soil fungal biodiversity.

## Materials and Methods

### Sampling and Pre-treatment

Within the period of July, 2011 to May, 2018, we collected 1251 composite soil samples comprising altogether > 50,000 individual samples in the projected area of 80,000 km^2^ in the northern Baltic region (Estonia and North Latvia; Supplementary Table S1 and Figure 1). The sites were selected based on aerial photographs, forestry and protected areas databases and haphazard visitation to cover a great variety of habitats. We also focused on old and protected habitats and monocultures of tree stands to improve our capacity to detect the effects of age and dominant plant species. Selection of the sites followed the basic criteria: (1) homogeneous area with no visible ecotones (except islands) and vegetation gradients; (2) >30 m distance from other vegetation type or trees belonging to species not present in the plot (to avoid influence by roots or litter), cutting of no more than 40% of trees basal area; and (3) no legal constraints.

As described in Tedersoo et al. (2014), we established circular 2500-m^2^ plots in homogeneous vegetation (avoiding ecotones) in forests, bogs, grasslands, croplands, parks and around abandoned buildings (ruins) of contrasting age and plant species composition (for ecosystem types and measured variables, see Supplementary Table S2). From each four quadrats of the plots, five trees located at least 8 m apart were randomly selected. From two opposing sides of each 20 trees per plot, 1–1.5 m from the tree trunk, soil cores (5 cm diam. to 5 cm depth) were collected using a sterilized PVC tube or sharp knife. In non-wooded habitats, randomly located spots were selected instead of trees for collecting samples. About 5–10 g of soil from the margins of each core was placed into a zip-lock plastic bag, with the interior of the soil core placed back into the hole. The material from all 40 cores per plot was pooled into the same bag, without removing fine roots or small stones (<1 cm diam.). The composite soil sample was laid on clean newspaper and air-dried as soon as possible, within at least 24 h since collection, at <40°C in a dry room, with heat from the sun, oven, light bulbs, floor heating system or other type of heating body. The newspaper was replaced when becoming moist to speed up the drying process. In a few exceptional cases (<2%), the sampling area was smaller (small island habitats) or larger (sparse woodlands) than 2500 m^2^ (for exceptions, see Supplementary Table S1).

### Molecular Analysis and Bioinformatics

The dried composite soil samples were kept in zip-lock plastic bags and homogenized manually by intensively rubbing the bag and its contents by hands for 3 min. DNA was extracted from 2.0 g of soil dust using the PowerMax Soil DNA Isolation kit (Qiagen, Carlsbad, CA, United States) following manufacturer’s instructions. The DNA extracts were further purified using FavorPrep^TM^ Genomic DNA Clean-Up Kit (Favorgen, Vienna, Austria).

The purified DNA extracts were subjected to amplification with the universal eukaryotic primers ITS9mun and ITS4ngsUni that have been nominated for global microbiome analyses of eukaryotes at species-level resolution (Tedersoo and Lindahl, 2016; Tedersoo and Anslan, 2019). These primers amplify 170 bp of the 18S rRNA gene and full-length Internal Transcribed Spacer (ITS), which serves as an official barcode for fungi (Schoch et al., 2012; Nilsson et al., 2018) and performs well on many groups of soil animals and protists (Coleman, 2009; Pawlowski et al., 2012). We selected this partial 18S + ITS marker for approximating operational taxonomic units (OTUs) at roughly species level, increased taxonomic resolution and higher capacity to detect and remove artifacts compared with regular short ITS1 or ITS2 and partial 18S markers (Tedersoo et al., 2018b). In spite of a high single-pass error rate, advances in PacBio third-generation sequencing technology enable producing circular consensus sequences (CCS) of >3000 bp with high quality.

For amplification, the PCR mixture comprised 5 μl of 5x HOT FIREPol Blend Master Mix (Solis Biodyne, Tartu, Estonia), 0.5 μl of each forward and reverse primer (20 mM), 1 μl of DNA extract and 18 μl ddH2O. Thermal cycling included an initial denaturation at 95°C for 15 min; 25–30 cycles of denaturation for 30 s at 95°C, annealing for 30 s at 57°C, elongation for 1 min at 72°C; final elongation at 72°C for 10 min and storage at 4°C. PCR products were normalized for library preparation and sequenced on PacBio Sequel instrument using SMRT cell 1M, v2 LR; Sequel Polymerase v2.1 and Sequencing chemistry v2.1. Loading was performed by diffusion, one SMRT cell was used for sequencing with a movie time of 600 min and pre-extension time of 45 min.

PacBio CCS reads (minPasses = 3, MinAccuracy = 0.9) were generated using SMRT Link v 6.0.0.47841. Subsequent quality filtering was performed using PipeCraft 1.0 (Anslan et al., 2017) as described in Tedersoo and Anslan (2019). UNITE 7.2 (Kõljalg et al., 2013) served as a reference database for chimera filtering and identification. Using UPARSE (Edgar, 2013), sequences were clustered to OTUs at 98% sequence similarity to enable separation of closely related species while keeping lower-quality sequences and rare variants adhered to OTUs’ centroids. Global singletons were removed. The entire workflow is given in Supplementary Table S3. For taxonomic assignments, we evaluated the 10 best hits and conservatively kept OTUs with conflicting best matches unidentified at the level of that taxonomic rank. The criteria for kingdom-level treatment included *e*-value < e^–20^ and sequence similarity > 70% (BLASTn values); that for phyla and class was *e*-value < e^–50^ and sequence similarity > 75%, whereas orders, families and genera were assigned at >80%, >85%, and >90% sequence similarity, respectively. Taxa falling below these thresholds were considered unknowns at particular taxonomic levels. Fungal taxa [taxonomy follows Tedersoo et al. (2018a) as updated in Wijayawardene et al., 2020] were used at the level of phyla (early diverging lineages), classes (Chytridiomycota, most Mucoromyceta) and orders (Dikarya, molds) to balance between taxonomic resolution and coverage.

To assign functions to fungal OTUs, we used two parallel approaches. First, we used the newly built FungalTraits database (Põlme et al., unpublished; beta version available at doi: 10.15156/BIO/807446) to assign OTUs to guilds (Supplementary Table S4) and EcM fungi further to lineages and exploration types. For genera with multiple lifestyles, we used the assignments based on annotations at the level of sequences and species hypotheses (SHs) as given in UNITE 8.1.

### Environmental Variables

During sampling, we recorded all woody plant and dominant herb species. Except for ericoid subshrubs, we estimated the relative basal area using direct measurements (Tedersoo et al., 2016) or visual estimates, for which participants were trained. For tree species, we considered presence when saplings were >1 m high. For non-wooded habitats, we estimated the proportion of plant species based on their coverage. For small-sized and rare species with no or minimal basal area, relative abundance of 0.1% was assigned. The age of vegetation was determined based on forestry databases, annual growth of trees based on branching, or circumference of largest trees at breast height (for deciduous trees > 200 cm circumference, 2 cm corresponds to 1 year based on increment cores). According to the evidence for mowing or grazing and growth of coppice, we estimated the management status for woodlands and parks (categories: managed, unmanaged and coppiced). Based on forestry databases or visual estimates, we determined the proportion of trees harvested and time since harvesting for selectively harvested or thinned stands. We considered old forests as virgin habitats, when these were at least 120 years old according to the oldest trees and were databased as old-growth forests or harbored large amounts of woody debris in all stages of decomposition and showed no visible evidence for historical human interference (e.g., no visible stumps).

Chemical properties of each composite sample were measured from 20 g of dried, homogenized material. Soil pH_*KCl*_, P, K, Mg, and Ca concentration as well as ^15^N and ^13^C abundances were determined following Tedersoo et al. (2012a). Concentrations and ratios of C/N, C/P and N/P were log-transformed for statistical analyses.

The climatic conditions in the region displayed little variation in mean annual temperature (5-7°C) and precipitation (500–750 mm). Although we expected that this has little effect on soil biota, we included a set of microclimatic variables (Karger et al., 2017) in our analysis (Supplementary Table S2).

Based on geographical coordinates of sample centroids, we calculated Moran’s Eigenvector Maps (MEMs) to represent geographic distance vectors (gMEMs; *n* = 319) as implemented in *adespatial* package (Dray et al., 2018) of R (R Core Team, 2019). We also calculated 74 temporal distance vectors (tMEMs) based on sampling dates. We treated year and month of sampling (February to April pooled; November to January pooled) as dummy variables and considered these and square-root-transformed linear time as covariates.

Phylogenies of woody plant species were adapted from Zanne et al. (2014). Based on an ultrametric tree, we calculated nearest neighbour (comdistnt), average neighbor (comdist) and phylosor distances for all samples as implemented in *picante* package (Kembel et al., 2010). Using *adespatial* package, we generated the respective 32 ntMEMs, 44 cdMEMs and 119 psMEMs based on these distances for use as covariates.

Based on estimates of basal area (or herb/shrub coverage), we calculated the relative abundance of EcM plants (Soudzilovskaia et al., 2020). Proportion of EcM plants and individual species were log-ratio-transformed (+0.01%) to vary from −4 (0.0%) to 0 (50.0%) to 4 (100.0%). The age of vegetation, number of woody plant species and EcM plant species sampled were used in both untransformed and square-root-transformed formats.

We also included dummy variables for collector, habitat type, selective harvesting or thinning, island type, management type (woodlands and parks only), presence of water bodies, and anthropogenic habitat (wild, village, urban). In more specific analyses and for illustration, these variables were used as multi-level categorical predictors (Supplementary Table S2).

As a proxy for fungal diversity, we used both OTU richness and Shannon diversity index. We also calculated OTU richness for dominant functional guilds, taxonomic groups and EcM lineages of fungi (Supplementary Table S4). All OTU richness measures were converted to residuals based on the average values of raw residuals taken from regression analyses of OTU richness vs. square-root-transformed sequencing depth and log-transformed sequencing depth. Averaging was used, because these functions tended to overestimate residual richness at either low or high sequencing depth. The relative sequence abundance-based proportions of fungal guilds, mold groups, EcM exploration types and individual OTUs were log-ratio-transformed.

### Pre-statistics Data Quality Evaluation

Before statistical analyses, we sorted the OTU matrices by negative and positive controls to seek for potential laboratory and technical contamination. The initial dataset#1 included 3,060,546 quality-filtered sequences from 1310 samples that were clustered into 23,787 OTUs (Table 1). We removed 59 control samples and 40 samples with low sequencing depth (<500 reads). Based on analysis of sequence distribution in control samples, tag switching and non-targeted markers, 28 OTUs were specifically excluded.

**TABLE 1:** Characteristics of data subsets used in various analyses.

Dataset	Plots	Sequences	OTUs	Purpose
dataset#1	1251 + 59 controls	3060546	23787	Quality control; removal of negative and positive controls, samples with low sequencing depth, OTUs of control, suspected of contamination and metagenomics origin
dataset#2	1211	2932102/2404613*/1169772**	23464/14881*/2782**	Mold control and analyses, removal of samples with low proportion of fungal sequences and those infested with molds
dataset#3	1175	2852942/2330914*/1162873**	23437/14866*/2781**	Main analyses, habitat type effect
dataset#4	1111	2237918*/1156228**	14217*/2750**	EcM-specific analyses
dataset#4a	1088	1141514**	2090**	EcM-specific community analyses (OTUs in > 4 plots, samples with > 20 EcM fungal sequences)
dataset#5	1051	2070903*/1080844**	13909*/2742**	Urbanization effect
dataset#6	784	1551976*/795056**	13197*/2663**	Island and fragmentation effects
dataset#7	58	125590*/72199*	5989*/1378**	Forest fragmentation effect
dataset#8	458	913658*/474225**	12104*/2543**	Virgin habitat effect
dataset#9	756	1533505*/809528**	12902*/2627**	Forest selective harvesting or thinning
dataset#10	157	261459*/148667**	7738*/1725**	Management of parks and woodlands

*^∗^Sequences of fungi. ^∗∗^sequences of ectomycorrhizal fungi.*

The resulting dataset#2 (1211 samples, 2,932,102 sequences and 23,464 OTUs) comprised on average 2421.2 sequences (*SD* = 1242.6; min = 504; max = 12763). We observed that a considerable proportion of samples revealed a high proportion of reads from molds (rapidly growing opportunistic saprotrophs), either a single high-abundance OTU or multiple OTUs from one or several phylogenetic groups. Previous studies suggested that high mold abundance is at least partly related to poor condition or poor preservation of samples (Tedersoo et al., 2014; Tedersoo et al., 2016). Due to high climatic variability or small sample size, we did not address this issue in-depth previously. Molds assigned to Mortierellales, Umbelopsidales, Mucorales and Pezizomycotina (mostly Eurotiales) contributed to 6.9 ± 4.8% (mean ± SD), 3.2 ± 7.5%, 0.2 ± 0.6%, and 2.4 ± 9.0% of sequences per sample, respectively, with maximum values between 12 and 90%. The negative effect of molds was tested on residuals of total fungal OTU richness, EcM fungal OTU richness and proportion of EcM fungal sequences, because we expected the latter obligately mutualistic group to be most responsive to molds (Tedersoo et al., 2014). The relative abundance of Umbelopsidales molds and proportion of most abundant OTU of Umbelopsidales had strongest negative correlations with overall fungal richness (*R* = −0.437; *P* < 0.001 and *R* = −0.325; *P* < 0.001, respectively), followed by negative effects of relative abundance of the most common Pezizomycotina mold OTU (*R* = −0.213; *P* < 0.001) and Mortierellales OTU (*R* = −0.140; *P* < 0.001). Residuals of EcM fungal richness were negatively affected by relative abundance of the most common Umbelopsidales OTU (*R* = −0.300; *P* < 0.001), Mortierellales OTU (*R* = −0.222; *P* < 0.001) and Mucorales OTU (*R* = −0.164; *P* < 0.001) as well as relative proportion of Mortierellales sequences (*R* = −0.189; *P* < 0.001). Random forest modeling (see below) confirmed that Mortierallales and Eurotiales mold relative abundances are among the strongest predictors of fungal richness, EcM fungal richness and EcM fungal proportion (Supplementary Figure S1). Accordingly, we removed 36 samples that were heavily occupied by molds (maximum OTU relative abundance > 30% in Pezizomycotina, >15% in Umbelopsidales, or >15% in Mortierellales molds).

Further analyses of molds revealed that relative abundance of Mortierallales and Umbelopsidales peaked at low-pH and C-rich forest soils, whereas Mucorales showed occasional high values in croplands. Pezizomycotina mold abundance was unrelated to any biological variables, suggesting that these molds are the most opportunistic but avoid samples where Mucoromyceta molds, especially Mortierellales are abundant. As relative abundances of all mold groups had strong temporal and/or collector biases, we conclude that their high abundance is related to either unfavorable meteorological conditions during the time of sampling (e.g., freeze-thaw and rewetting of dry soils) and/or differential post-sampling sample spoilage (too slow or incomplete drying) in hands of collectors. More detailed information about the environmental and technical variables driving mold abundance is given in Supplementary Figure S2 and Supplementary Table S5.

### Statistical Analyses

We used the mold-corrected dataset#3 for the main analyses (Table 1), including the proportion of molds as covariates because of their potential residual effects. First, we used the random forest machine learning algorithm to generate non-linear models for all richness variables using combined features of randomforest (Liaw and Wiener, 2002) and VSURF (Genuer et al., 2019) packages in R. Model evaluation was performed using 999 trees. Because these analyses provide no information about the type of fit or determination coefficient, we tested these pre-selected best predictors by fitting quadratic functions and general linear modeling for each dependent variable as implemented in Statistica 13 (TIBCO Software Inc., NY, United States). For EcM fungi, we removed 64 non-EcM plots and samples containing < 100 EcM fungal sequences (dataset#4). The above-described model selection procedure was used for richness residuals of EcM fungi and EcM fungal lineages and relative abundance of exploration types. We also calculated niche models for 50 most frequent EcM and non-EcM fungal OTUs using log-ratio-transformed relative abundances. We further used piecewise structural equation modeling to delve into direct and indirect relationships between OTU richness and the key potential predictors, as implemented in piecewiseSEM package (Lefcheck, 2016) of R.

**FIGURE 2:**
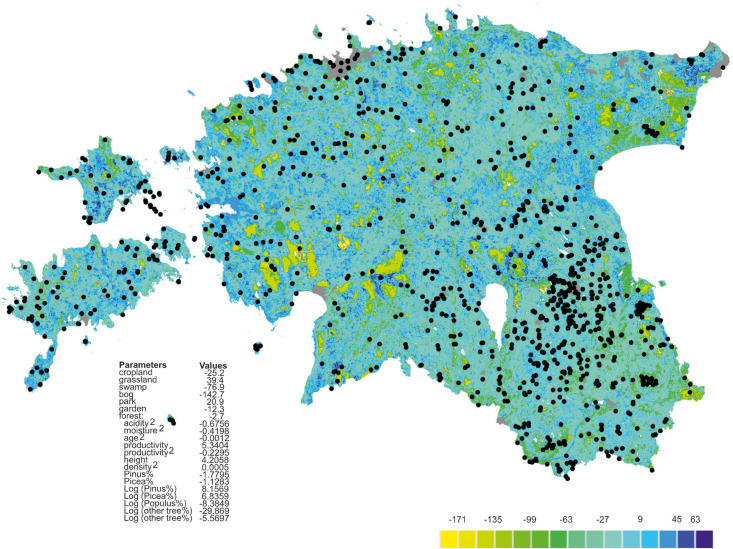
Extrapolated fungal richness map of Estonia. Colors represent relative richness of fungi accounting for land use type and estimated soil litho-genetical scalar (acidity), estimated soil moisture scalar (humidity) and vegetation data as based on the best GLM model. Gray represents towns, roads and buildings; black circles represent plots.

Richness residuals of fungi (Figure 2) and major fungal guilds were extrapolated to a land use and forest map of Estonia at 250 × 250 m resolution based on available map layers. The estimates of stand productivity were obtained from State Registry of forest data and soil map (Environment Agency, Tartu^1^). Proportions of tree species (Lang et al., 2018) and stand height, volume and stand density (Lang et al., 2012) were calculated by equations from the Regulation of Ministry of Environment (Regulations of the Forest Survey, 2018; Regulation of Minister of the Environment No 2. Enforced 16.01.2009.). The estimated soil acidity and humidity scalars were adopted from the Estonian Soil Classification for Postlithogenic normally developed mineral soils (Astover et al., 2012) by converting from specific average values of soil types^2^. The extrapolated fungal richness values were transferred to the Estonian base map^3^.

To infer the most influential variables affecting the composition of fungi, we constructed multivariate PERMANOVA models (Anderson et al., 2008), followed by supporting analyses using Non-metric Multidimensional Scaling (NMDS) in vegan package of R (Oksanen et al., 2019) and estimates of potential non-linear effects as implemented in General Dissimilarity Modeling (GDM; Manion et al., 2018). For these multivariate analyses, environmental variables were normalized, abundances of OTUs were Hellinger-transformed, and Bray-Curtis dissimilarity was used as a distance metric. We assessed three broad groups of fungi: (1) EcM fungi including log-ratio-transformed proportions of lineages in a separate analysis; (2) putative plant pathogen guilds combined; (3) saprotrophs combined (excluding taxa also classified as plant pathogens). In all cases, samples with <20 OTUs in the particular categories were excluded from analyses. For the NMDS graphs, we fitted the environmental predictors and calculated 95% confidence ellipses using the vegan functions envfit and ordiellipse.

## Results and Discussion

### Taxonomic and Functional Assignment of Fungi

**FIGURE 3:**
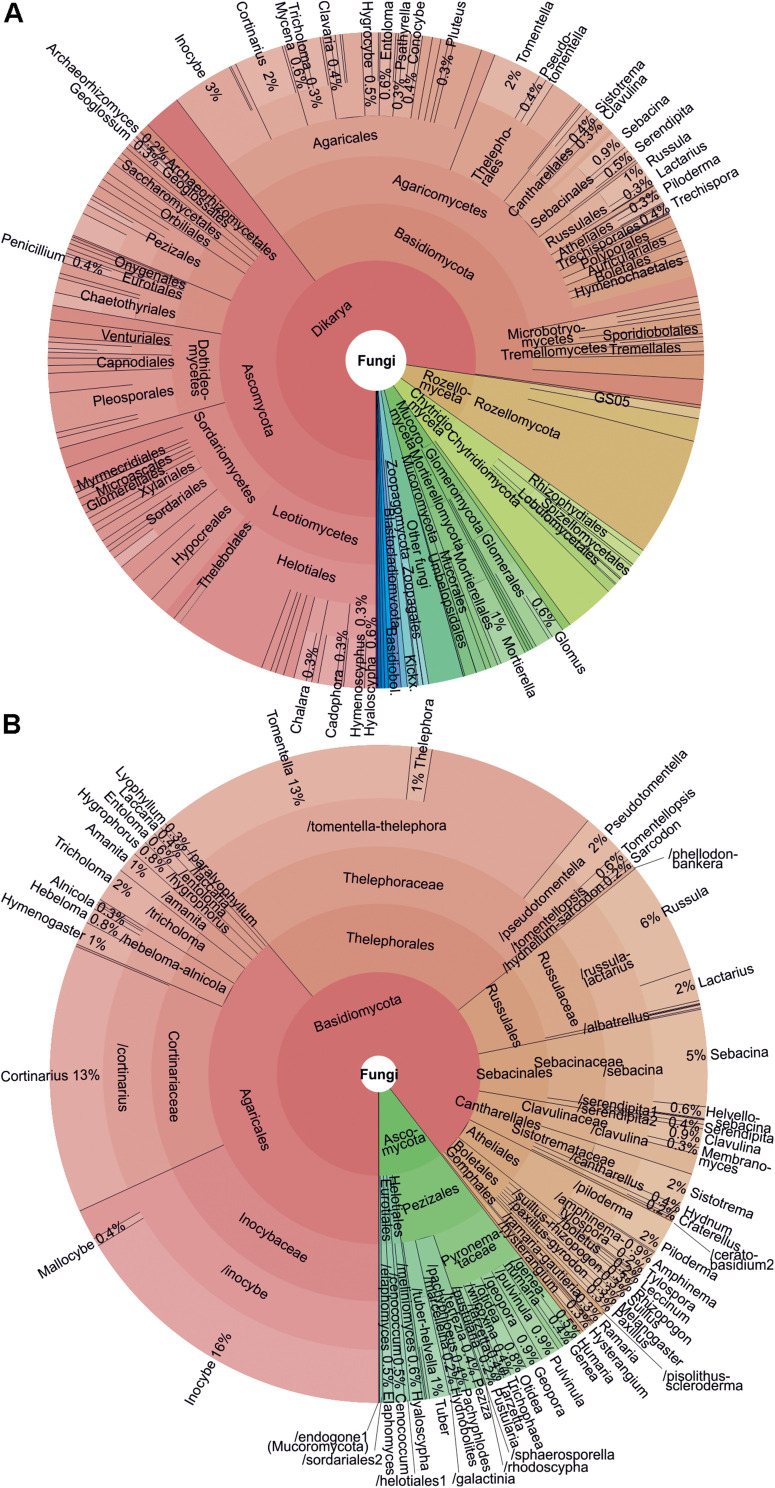
Krona charts showing **(A)** taxonomic distribution of all fungi and **(B)** ectomycorrhizal fungi as based on OTU richness.

After several rounds of data quality-filtering, the main dataset#3 (1175 samples and 2,852,942 sequences) included 23,437 OTUs at 98% sequence similarity threshold. Altogether 14,866 OTUs (63.4% of OTUs; 81.7% of sequences) belonged to fungi, followed by Alveolata (10.3%; 9.3%), Metazoa (8.7%; 3.5%), Rhizaria (5.8%; 1.2%), Plantae (3.3%; 2.7%), and Stramenopila (1.7%; 0.7%). Taxonomically unassigned eukaryotes contributed to 0.8% of sequences and 5.3% of OTUs. Across all organisms, the dominant OTUs included the saprotrophic mold *Mortierella verticillata* (SH196779.07FU; 1.7% sequences; 80.7% samples), saprotrophic yeast *Solicoccozyma terricola* (SH190017.07FU; 1.6%; 87.8%) and previously unsequenced Oxytrichidae ciliate (SH1415986.08FU; 1.4%; 97.5%). In fungi, all recognized phyla and phylum-level groups were detected (Figure 3). The most abundant and diverse fungal phyla included Ascomycota (39.6% fungal OTUs; 25.8% fungal sequences), Basidiomycota (37.6%; 58.1%), Mucoromycota (1.1%; 3.6%), Mortierellomycota (2.1%; 8.7%), Chytridiomycota (5.1%; 1.4%), Rozellomycota (7.5%; 1.4%), Glomeromycota (2.1%; 0.2%) and Zoopagomycota (1.0%; 0.3%). Altogether 258 putative fungal OTUs (1.7%) representing 0.2% of sequences remained unidentified at the phylum level.

**FIGURE 4:**
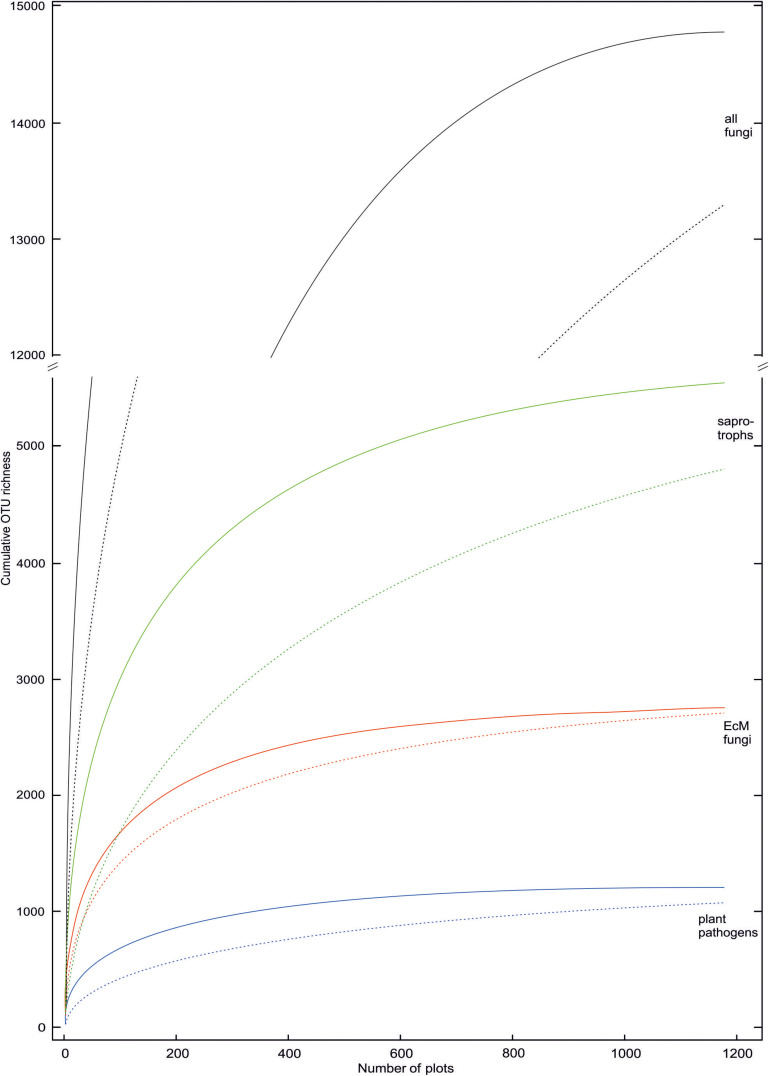
Rarefied OTU accumulation curves for all fungi and ectomycorrhizal, saprotrophic and plant pathogenic groups therein. Solid and dashed lines indicate inclusion and exclusion of sample-wise singletons (one sequence in a sample) in samples, respectively. These singletons are locally rare OTUs that may potentially represent index-switching artifacts.

Taxa with filamentous mycelium prevailed (50.7% of OTUs; 82.3% of sequences); zoosporic groups combined (6.2%; 1.7%), yeasts (2.2%; 4.9%), putative dimorphic yeasts (2.4%; 0.9%) and other unicellular-aflagellate life forms (7.5%; 1.4%) were less common. Ectomycorrhizal mutualists (18.7%; 49.9%); putative root endophytes (1.1%; 0.9%), leaf/seed/fruit pathogens (6.6%; 5.4%), animal parasites (2.8%; 1.8%), mycoparasites (1.5%; 0.7%) and decomposers of litter (11.9%; 8.0%), soil (9.7%; 19.6%, including molds), wood (6.3%; 2.5%), animal material (1.3%; 0.8%) and dung (1.0%; 1.2%) were the most common functional guilds. Putative opportunistic human pathogens accounted for 2.4% OTUs and 2.3% sequences, but these values are probably inflated because of genus-level assignments, as only a few species in these genera are known as potential pathogens. Treating the proportion of sequences as a rough proxy for proportional biomass, filamentous fungi, particularly ectomycorrhizal (EcM) fungi and most saprotroph guilds exhibited relatively low OTU richness to sequence abundance ratio, whereas arbuscular mycorrhizal (AM) fungi, most parasite guilds and unicellular groups (except yeasts) were more diverse compared to their relative abundance. This suggests that broader sampling and particularly deeper sequencing would increase the proportion of fungi with tiny body size in diversity surveys (Figure 4).

The observed taxonomic and functional distribution of fungi corroborates global-scale patterns (Tedersoo et al., 2014; Egidi et al., 2019; Vetrovsky et al., 2019), with somewhat higher proportions of EcM fungi and early diverging lineages. EcM fungal dominance is attributable to prevalence of EcM tree-dominated forest habitats in our sampling and in North European temperate and boreal forest biomes in general, compared with most other global biomes, especially drylands (Soudzilovskaia et al., 2019). The relatively greater diversity of early-diverging lineages is at least partly related to the use of pan-eukaryotic primers and a marker comprising full-length ITS and flanking 18S V9 variable region (see section “Methodological Implications”), but it may also be related to their greater beta diversity.

### Diversity of Fungi and Major Fungal Guilds

**FIGURE 5:**
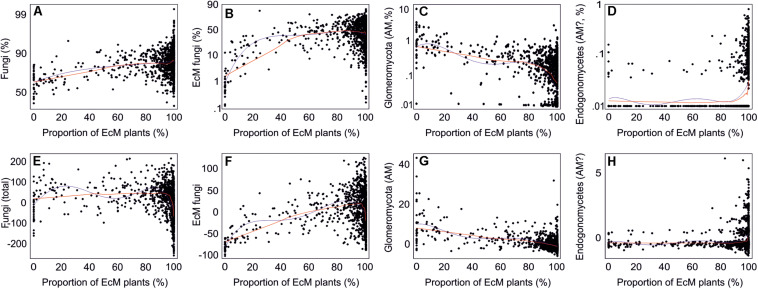
Relationships between the proportion of ectomycorrhizal plants and proportion of **(A–D)** sequences (log-ratio scale) or **(E–H)** OTUs (residuals) of all fungi and mycorrhizal fungi. Blue and red lines indicate best-fitting quadratic polynomial and lowess regression lines, respectively.

**FIGURE 6:**
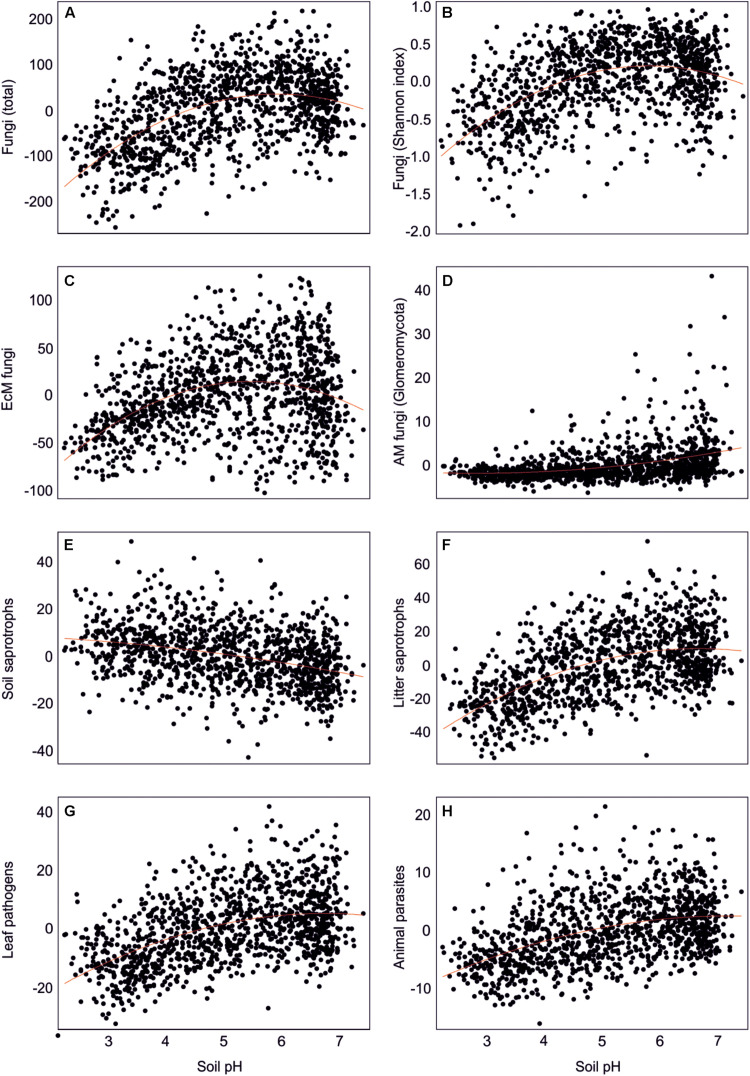
Effect of soil pH on residual richness of fungi: **(A)** all fungi, **(B)** fungi Shannon index, **(C)** ectomycorrhizal fungi, **(D)** arbuscular mycorrhizal fungi, **(E)** soil saprotrophs, **(F)** litter saprotrophs, **(G)** leaf pathogens, and **(H)** animal parasites.

**FIGURE 7:**
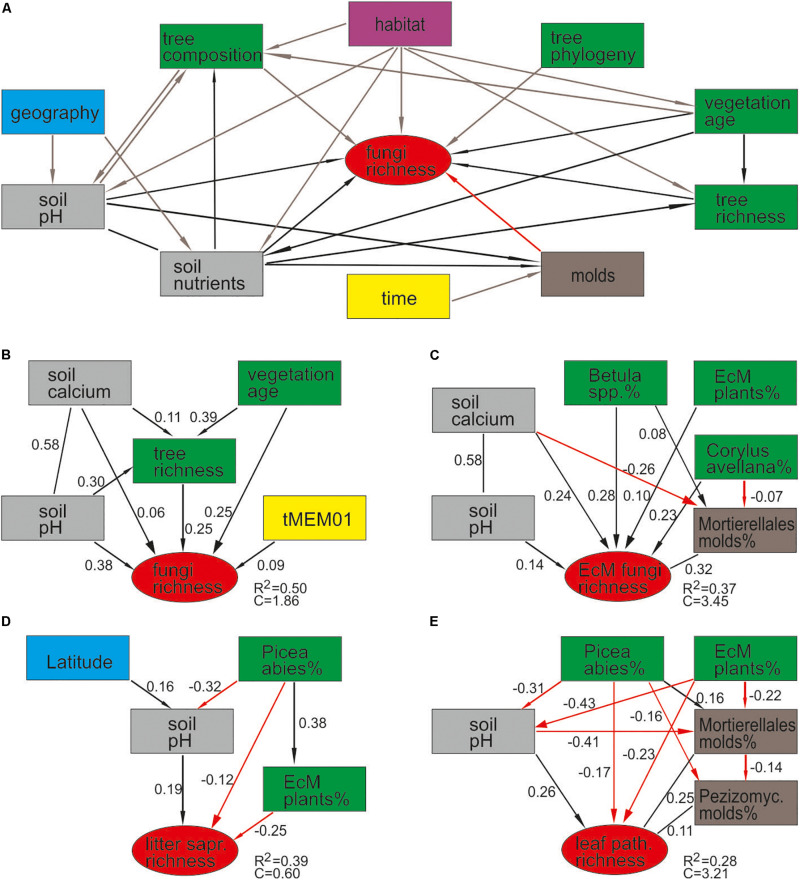
Structural equation models for predicting fungal richness: **(A)** *a priori* model based on expected relationships; AICc-based best models for **(B)** all fungi, **(C)** ectomycorrhizal fungi, **(D)** litter saprotrophs, and **(E)** leaf pathogens.

By combining the random forest machine learning and general linear modeling (dataset#3), we found that the overall proportion of fungi among eukaryote sequences responded positively to EcM plant proportion (*R*^2^ = 0.098; Figure 5) and negatively to soil P concentration (*R*^2^ = 0.096). Soil pH was the main predictor for richness of most fungal functional and taxonomic groups (Table 2, Figure 6, and Supplementary Figure S3). Soil pH (unimodal relationship) and plant richness (positive effect) had strongest effects on fungal OTU richness and Shannon index (Figures 5, 6), with an additional effect of community age (sigmoid relationship). Structural equation models (SEMs) confirmed these patterns and suggested that vegetation age has positive effects on fungal richness directly and through increasing plant diversity (Figure 7).

**TABLE 2:** Best models explaining OTU richness of fungi, major taxonomic groups and guilds therein (dataset#3).

Dependent variable	MSE_*total*_ (random forest)	Predictors (directionality^1^, MSE of random forest; variation explained based on GLM,%)
Fungi (all)	64.7	Plant richness (↗35.0;22.5), pH (∩31.5;**43.0**), age (*∫*24.0;12.8), tMEM01 (22.5;1.5), Ca (*Γ* 19.2;1.4)
Fungi (Shannon index)	56.1	pH (∩29.3;**34.2**), plant richness (↗27.6;11.4), Mortierellales mold% (∩25.9; 8.9)
**Functional guilds of fungi**		
Arbuscular mycorrhizal fungi	40.4	EcM plant% (↘28.3;**23.7**), C/N (↘16.8;2.4)
Ectomycorrhizal fungi	64.5	Mortierellales mold% (↘38.1;3.6), EcM plant% (↗35.1;**19.0**), Ca (↗29.4;3.7), pH (∩28.4;10.2), *Corylus*% (*Γ* 27.4;5.2), *Betula*% (*Γ* 25.2;8.4)
Root endophytes	68.0	pH (↘39.8;**50.7**), C/N (↗23.2;18.2), *Pinus*% (↗18.8;9.0)
Dung saprotrophs	57.5	pH (↗38.3;**41.9**), C/N (↘27.8;2.0), δ^15^N (↗20.7;7.2)
Litter saprotrophs	61.3	pH (∩34.1;**35.4**), EcM plant% (↘26.8;8.8), *Picea*% (↘24.0;3.1)
Soil saprotrophs	34.8	pH (↘36.0;**13.2**), age (*Γ* 22.9;7.4), time (20.9;4.8), Ca (∩15.2;1.2)
Wood saprotrophs	44.3	Mg (∩22.6;**19.5**), *Picea*% (↘22.3;4.4), EcM plant% (∩18.7;7.1), tMEM02 (16.7;2.9)
White-rot decomposers	34.8	δ^15^N (↘24.7;**8.4**), *Alnus*% (↗20.4;7.3), tMEM02 (19.6;2.6), age (*Γ* 15.6;4.7), pH (∩12.9;1.7)
Leaf pathogens	50.8	pH (↗32.0;**24.1**), *Picea*% (↘25.4;4.4), Mortierellales mold% (↘22.9;6.1), Pezizomycotina mold% (↘19.4;2.2), EcM plant% (↘17.6;5.4)
Wood pathogens	33.4	pH (∩27.2;5.7), plant richness (↗25.1;**14.1**),
Animal parasites	47.9	pH (*Γ* 29.9;**21.9**), time (25.3;4.0), P (↗19.8;6.3), EcM plant% (↘18.7;5.4)
Opportunistic human pathogens	32.8	time (25.9;9.4), pH (↗20.6;**13.2**), tMEM03 (20.2;0.4), *Picea*% (↘16.8;2.8), year 2017 (13.4;3.0), EcM plant% (↘11.3;2.8)
Mycoparasites	25.8	time (22.4;**10.4**), pH (∩21.8;3.2), tMEM03 (17.3;-), Ca (∩15.4;2.6), tMEM01 (15.0;1.8)
Yeasts	20.6	age (↗22.2;**8.1**), *Picea*% (↘15.4;2.0), tMEM03 (13.8;1.0)
Dimorphic yeasts	16.2	*Picea*% (↘22.1;**6.1**), tMEM03 (14.6;0.4)
**Taxonomic groups of fungi**		
Aphelidiomycota	30.6	pH (↗19.4;10.5), *Populus x wettsteinii*% (↗17.2;**11.8**), C/N (↘16.3;−), δ^15^N (↗14.3;1.8), Mortierellales mold% (↘13.4;1.3)
Basidiobolomycota	4.3	*Populus x wettsteinii*% (↗14.1;**4.0**), C/N (↘12.5;0.9), pH (∩10.0;0.4)
Blastocladiomycota	6.7	P (↗13.5;**1.1**)
clade GS01	2.0	pH (↘10.8;**1.3**)
Monoblepharomycota	19.0	*Populus x wettsteinii*% (18.1;**8.0**), δ^13^C (↘12.2;1.1)
Neocallimastigomycota	7.6	*Populus x wettsteinii*% (↗15.2;**11.1**)
Olpidiomycota	23.0	tMEM01 (19.5;2.8), pH (∩17.3;**5.5**), Ca (∩15.9;1.8), age (↗12.9;5.3)
Rozellomycota	30.9	Mg (↗28.5;**7.2**), *Alnus*% (↗16.3;3.6)
Zoopagomycota	22.9	Mg (↗14.5;**1.6**)
Chytridiomycetes	13.8	pH (↘24.5;**4.1**), C/N (↗13.0;0.4)
Lobulomycetes	26.1	C/N (↘1.0;-), pH (*Γ* 21.5;**10.1**)
Rhizophlyctidomycetes	58.7	pH (*Γ* 38.0;**30.6**), C/N (U 30.5;4.9), EcM plant% (↘29.7;9.6), *Picea*% (↘23.4;4.7), tMEM01 (22.4;3.2)
Spizellomycetes	19.8	tMEM05 (16.1;1.0), tMEM01 (13.1;1.0), tMEM10 (11.7;3.2), EcM plant% (↘11.2;**6.9**), tMEM04 (9.9;0.7)
Endogonomycetes	36.7	pH (↘25.8;**23.9**), Ca (↘16.6;0.9)
Mortierellomycetes	32.8	pH (∩21.3;**8.1**), *Pinus*% (∩17.5; 3.7), *Padus*% (↗16.8;5.3), time (16.1;1.3)
Mucoromycetes	19.4	time (16.8;**4.0**), tMEM06 (15.6;3.7)
Umbelopsidomycetes	66.3	pH (↘60.0;**51.4**), δ^15^N (↘32.8;5.3), C/N (∩28.3;5.0)
Diversisporales	9.2	EcM plant% (↘17.5;**3.3**), gMEM009 (13.9;-), C/N (↘12.6;0.4), *Ulmus glabra*% (↗10.0;0.4)
Glomerales	40.8	EcM plant% (↘25.7;**25.7**), *Acer*% (↗16.2;-), C/N (↘16.0;1.2), Umbelopsidales mold% (↘12.3;5.1)
Archaeorhizomycetales	41.9	*Pinus*% (↗21.7;**29.0**), C/N (∩17.9;2.9), pH (↘14.2;1.1)
Capnodiales	26.9	tMEM02 (19.2;**11.1**), tMEM03 (17.7;1.6), time (16.4;0.8), tMEM01 (10.8;2.0), year 2017 (10.3;-)
Chaetosphaeriales	24.9	*Quercus*% (↗23.2;**6.5**), δ^13^C (↘21.7;5.8), pH (∩14.7;3.0), *Alnus*% (∩11.8;3.3), plant richness (↗11.4;0.7)
Chaetothyriales	15.9	Ca (↘16.4;0.7), *Fraxinus*% (↗16.0;-), C/N (∩15.8;**1.9**), tMEM02 (11.3;1.2)
Coniochaetales	21.7	P (↘16.6;**4.2**)
Eurotiales	28.6	time (23.5;6.9), *Quercus*% (↗20.5;**9.5**), tMEM03 (18.0;0.5), year_2017 (12.3;2.0), tMEM02 (10.5;1.1), tMEM01 (10.2;1.7), age (↗8.6;6.7)
Geoglossales	22.3	woodland (↗17.5;**7.6**), EcM plant% (↘14.9;6.2), *Acer*% (↗14.9;-), δ^15^N (↗10.0;-)
Glomerellales	43.1	pH (↗28.1;**29.2**), *Picea*% (↘20.6;5.0), Umbelopsidales mold% (↘19.3;2.7), δ^15^N (↗17.8;2.1), C/N (↘17.5;-), Umb max (↘13.9;-), EcM plant% (↘13.1;1.5)
clade GS27	9.7	C/N (↘15.4;1.5), pH (↘12.8;0.8), δ^15^N (↘10.9;**1.6**), *Picea*% (↗9.8;1.2)
Helotiales	39.0	age (*Γ* 22.7;10.7), plant richness (↗20.9;6.2), pH (∩19.9;**11.6**), δ^13^C (↘18.3;1.2), gMEM004 (15.0;0.4); δ^15^N (∩14.7;1.0), EcM plant% (↗14.2;-)
Hypocreales	59.0	pH (↗36.9;**36.4**), C/N (↘24.1;-), time (23.1;9.9), δ^15^N (↗22.0;3.5), tMEM03 (21.1;0.5)
Microascales	42.1	δ^15^N (↗31.9;**27.5**), C/N (↘17.1;-), Umbelopsidales mold% (↘14.3;2.8)
Myrmecridiales	26.5	pH (↗28.8;5.9), *Padus*% (↗17.6;**8.3**), EcM plant% (∩16.0;1.2), C/N (↘15.9;1.7), δ^13^C (↘14.7;4.2)
Onygenales	31.6	C/N (↘23.0;-), δ^15^N (↗22.2;**21.8**), pH (↗16.9;4.1)
Orbiliales (s.lat)	16.1	*Picea*% (↘12.3;1.1), K (↗10.5;**5.7**), tMEM02 (9.8;3.3)
Pezizales	62.9	pH (↗59.8;**54.7**), Ca (↗24.8;1.0), *Quercus*% (*Γ* 18.2;5.7)
Pleosporales	57.1	pH (↗34.9;**36.2**), *Picea*% (↘23.3;10.0), δ^15^N (↗21.0;5.6), Pez mold% (↘19.2;0.3)
Saccharomycetales	25.8	C/N (↗28.6;2.1), pH (U 14.3;**11.2**)
Sordariales	59.3	pH (↗37.5;**43.4**), *Picea*% (↘26.1;9.4), δ^15^N (∫25.2;6.9)
Thelebolales	38.1	C/N (↘29.1;1.8), pH (∩18.8;**50.0**), age (↗17.2;5.7) time (17.2;4.5)
Venturiales	35.7	δ^13^C (↘27.6;1.7), pH (∩23.1;**7.4**)
Xylariales	36.1	pH (↗31.5;**23.4**), *Picea*% (↘28.4;1.8), *Tilia*% (↗19.7;4.5), EcM plant% (↘18.3;1.7)
Agaricostilbomycetes	25.3	pH (*Γ* 25.7;**15.3**), C/N (↘19.6;-), *Corylus*% (↗18.8;6.3), Ca (↗12.7;0.4), P (↘11.6;3.4)
Geminibasidiomycetes	37.1	*Corylus*% (↗25.8;4.9), P (↘25.4;7.0), Mg (↘21.4;**10.5**),
Microbotryomycetes	12.9	age (↗14.3;**2.5**)
Agaricales (non-EcM)	39.2	plant richness (↗26.7;**20.3**), tMEM02 (19.1;4.8), EcM plant% (∩17.6;3.2), pH (∩16.5;6.2)
Auriculariales	15.7	tMEM02 (19.0;0.8), C (↗15.8;**6.9**), C/N (↗15.6;-), N (↗11.7;−), Ca (*Γ* 10.1;0.7)
Ceratobasidiaceae	28.8	Umbelopsidales mold% (↘17.7;2.4), δ^15^N (↗17.5;**15.4**), tMEM02 (14.6;1.1), EcM plant% (↘13.9;3.0), Umb max (↘12.7;-), forest (↘12.4;3.2), C/N (↘11.5;0.4)
Cystofilobasidiales	26.6	EcM plant% (↘21.5;2.8), pH (↗17.6;**15.6**), C/N (↘15.3;0.6)
Filobasidiales	17.2	time (16.5;3.4), pH (↗15.4;**4.2**), Umbelopsidales mold% (↘13.9;-), Umb max (↘13.8;-), tMEM01 (12.2;-), EcM plant% (↘10.5;0.3)
Hymenochaetales	25.3	*Picea*% (*Γ* 32.3;**12.5**), δ^15^N (↘20.9;6.8), pH (↘19.1;0.6)
Polyporales	19.3	pH (∩14.9;**8.1**), gMEM003 (14.5;-)
Sebacinales (non-EcM)	21.9	C/N (*Γ* 32.1;**6.0**), *Pinus*% (↗14.0;1.4), age (↘13.5;-)
Trechisporales	44.1	pH (↘41.5;**20.2**), δ^15^N (↘22.0;2.1), C (↗18.7;10.7), *Picea*% (↗16.7;1.3)
Tremellales	18.3	*Picea*% (↘28.3;**4.6**), tMEM10 (13.9;1.2), tMEM13 (12.5;2.8)
Tremellodendropsidales	13.5	pH (∩18.9;**6.1**), plant richness (↗12.9;3.0), Ca (*Γ* 12.1;0.4)
Trichosporonales	14.6	pH (∩24.0;**13.3**)
unknown fungi	36.2	*Populus x wettsteinii*% (↗20.9;**16.8**), EcM plant% (↘18.8;10.5); *Picea*% (↘16.9;3.9)

*The best GLM predictors are given in bold. All effects are significant (P < 0.001). ^1^Symbols in continuous predictors: ↗, near-linear increase; ↘, near-linear decline; ∫, sigmoid increase; Γ, cumulative increase; ∩, unimodal. Subgroups of response variables are indicated in bold.*

Ectomycorrhizal fungal richness was most strongly related to soil Ca concentration (positive effect; *R*^2^ = 0.260), followed by relative abundance of *Betula* spp. (*R*^2^ = 0.213), *Corylus avellana* (*R*^2^ = 0.136) and EcM plants taken together (*R*^2^ = 0.135), and soil pH (*R*^2^ = 0.084; all unimodal responses; Figure 7). A more detailed analysis showed that the relative abundance of EcM fungi reached a plateau at around 50% EcM plant proportion (Figure 5). Conversely, richness of the AM Glomeromycota was negatively related to EcM plant basal area (*R*^2^ = 0.237) and soil C/N ratio (*R*^2^ = 0.024). Relative abundance of Glomeromycota declined linearly from 0 to 90% EcM plant basal area, with a sharper drop beyond 90% (Figure 5), whereas Glomeromycota richness decreased linearly throughout the range of EcM plant proportions. By contrast, relative abundance and richness of putatively AM Endogonomycetes (including a single rare EcM fungal OTU) increased at around 98% of EcM plant proportion (Figure 5) that coincided with acidic pine forest habitats. There was a negative correlation between the abundance of Endogonomycetes and Glomeromycota (*R* = −0.205), which we attribute to their contrasting ecological preferences rather than competition. Although several Endogonomycetes OTUs matched closely to the AM fine root endophytes recovered in previous studies (Orchard et al., 2017), our results provide circumstantial evidence that most of these taxa are not obligate AM symbionts. Our results furthermore suggest that AM Glomeromycota are more sensitive to relative abundance of their host plants compared with EcM fungi, which maintain high biomass and richness at lower proportions of their host plants. These differences indicate that soil and ecophysiological processes such as slow nutrient cycling and neutral or positive plant-soil feedback and low bacterial diversity and activity characteristic of EcM habitats (Phillips et al., 2013; Tedersoo and Bahram, 2019; Bahram et al., 2020; Tedersoo et al., 2020), may also prevail in mixed forests.

Richness of all saprotrophs taken together was best predicted by proportion of EcM plants (negative effect, *R*^2^ = 0.261) and soil pH (peak at 5.5–7; *R*^2^ = 0.083), but best predictors differed among guilds (Table 2). Litter decomposers prevailed at near-neutral pH (pH 6–7; *R*^2^ = 0.354) and responded negatively to proportion of EcM plants (*R*^2^ = 0.088), particularly *Picea abies* (*R*^2^ = 0.031), with synergistic effects between pH and tree composition (Figure 7). Soil Mg concentration (*R*^2^ = 0.195) and proportion of EcM plants (*R*^2^ = 0.071) best explained wood saprotroph richness, both with unimodal relationships. Diversity of white-rot decomposers responded negatively to soil δ^15^N (*R*^2^ = 0.084) but positively to proportion of *Alnus* spp. (*R*^2^ = 0.073) and vegetation age (lag at 100 years; *R*^2^ = 0.047). Soil saprotrophs responded negatively to soil pH (*R*^2^ = 0.132) and positively to age (lag at ca 140 years; *R*^2^ = 0.074). Dung saprotrophs had a pH optimum at pH > 6 (*R*^2^ = 0.419) and positive response to soil δ^15^N (*R*^2^ = 0.072). The strong negative effect of EcM plant relative abundance on litter saprotrophs is attributable to reduced soil pH and competition between EcM and saprotrophic fungi for nutrients bound in soil organic material (Sterkenburg et al., 2018).

Leaf pathogens prevailed at weakly acidic to neutral soils (*R*^2^ = 0.241) and low relative abundance of EcM plants (*R*^2^ = 0.054), in particular *Picea abies* (*R*^2^ = 0.044). Wood pathogens were positively related to woody plant richness (*R*^2^ = 0.141) and soil pH (peak at 4.5–6.5; *R*^2^ = 0.057). Animal parasites were most diverse in neutral soils (pH > 6; *R*^2^ = 0.219) with high P concentration (*R*^2^ = 0.063). Richness of OTUs classified as opportunistic human pathogens similarly prevailed at high pH (pH > 6; *R*^2^ = 0.132) but exhibited strong temporal shifts (*R*^2^_*total*_ = 0.128). Similarly, mycoparasite richness was mostly explained by various temporal vectors (*R*^2^_*total*_ = 0.122), with weak unimodal responses to soil pH (*R*^2^ = 0.032) and Ca concentration (*R*^2^ = 0.026). Similarly to molds, mycoparasites and fungal decomposers take advantage of nutrients released from dying mycelium, which is related to annual and intra-annual cycles, confirming the temporal patterns in various biomes (He et al., 2017; Castano et al., 2018b; Burke et al., 2019). The positive effects of soil pH and negative effects of EcM vegetation on plant pathogens support the observations that pathogens drive plant community dynamics in AM-dominated forests (Bennett et al., 2017; Chen J. et al., 2019).

Yeasts were dominated by members of Basidiomycota (Tremellomycetes and Pucciniomycotina). The richness of this entire life form increased with vegetation age (*R*^2^ = 0.081). Dimorphic yeasts, mostly represented by members of Herpotrichiellaceae, were negatively affected by relative abundance of *Picea abies* (*R*^2^ = 0.061).

### Richness of Fungal Taxonomic Groups

Fungal taxonomic groups differed strongly in the best richness predictors, with soil pH and individual tree effects prevailing (Table 2 and Supplementary Figures S3, S4). Early-diverging fungal lineages (Aphelidiomycota, Basidiobolomycota, Monoblepharomycota, Neocallimastigomycota) as well as ‘unknown’ fungi, most of which probably represent divergent groups within unicellular fungi, were relatively common and diverse in plantations of *Populus* × *wettsteinii* (hybrid aspen). Blastocladiomycota richness responded positively to soil P concentration (*R*^2^ = 0.011). Soil pH was the strongest predictor for clade GS01 (negative effect; *R*^2^ = 0.013) and Olpidiomycota (unimodal relationship; *R*^2^ = 0.055), whereas soil Mg concentration positively affected the richness of Rozellomycota (*R*^2^ = 0.072) and Zoopagomycota (*R*^2^ = 0.016). Of Chytridiomycota classes, Chytridiomycetes (negative effect; *R*^2^ = 0.041), Lobulomycetes (positive effect; *R*^2^ = 0.101) and Rhizophlyctidomycetes (positive effect: *R*^2^ = 0.306) responded most strongly to soil pH, whereas proportion of EcM plants affected Spizellomycetes (negative effect; *R*^2^ = 0.069). Apart from Mucoromycetes (mostly Mucorales molds), OTU richness of Mucoromyceta classes was driven by soil pH. In particular, Umbelopsidomycetes (molds) and Endogonomycetes (non-molds) preferred highly acidic soil, whereas Mortierellomycetes (molds) prevailed in moderately acidic soils (pH 4–5). The two most common groups of Glomeromycota, Diversisporales and Glomerales were both negatively affected by EcM tree basal area, with other biological effects of negligible importance (Table 2).

In Ascomycota, the orders Glomerellales, Hypocreales, Sordariales, Xylariales (all Sordariomycetes), Pezizales and Pleosporales were more diverse in soils with neutral pH, whereas Helotiales, Thelebolales (both Leotiomycetes) and Venturiales had pH optima at moderately or weakly acidic soils (Table 2 and Supplementary Figure S3). Archaeorhizomycetales richness was associated with strongly acidic soils in pine-dominated forests. Interestingly, Saccharomycetales yeasts were most diverse at both ends of the pH gradient, confirming their realized niche in extreme habitats (Kurtzman et al., 2011). Richness of Chaetosphaeriales and Eurotiales was positively related to the relative abundance of *Quercus robur* (Supplementary Figure S4). In Basidiomycota, the Tremellomycetes orders Cystofilobasidiales and Filobasidiales were relatively more diverse at neutral pH, whereas Trichosporonales prevailed at moderately acidic habitats and Tremellales had no particular pH preference (Table 2). Of Agaricomycetes, Trechisporales, Hymenochaetales, Polyporales and Tremellodendropsidales were associated with highly or moderately acidic soils, but non-EcM OTUs of Agaricales prevailed in weakly acidic soils (Supplementary Figure S3). The saprotroph/necrotrophic pathogen family Ceratobasidiaceae was most diverse at high δ^15^N in non-forest habitats.

Although plant diversity was one of the strongest predictors of overall fungal diversity, it contributed *directly* to OTU richness of non-EcM Agaricales (*R*^2^ = 0.203) and Helotiales (*R*^2^ = 0.062) only. SEM revealed that tree richness had a direct positive effect on fungal richness, which was further promoted by vegetation age, soil pH and Ca that all exhibited direct residual effects as well (Figure 7). These results suggest that the positive effect of plant richness previously observed on local (Tedersoo et al., 2016) and regional (Yang et al., 2017) scales represents a synergistic interaction among multiple coevolutionary as well as biotic and abiotic niche differentiation processes that favor richness of both groups. On a global scale, historical processes, dispersal limitation and differential tolerance to climatic conditions have effectively blurred the local-scale causal links and correlation between plant and fungal richness (Tedersoo et al., 2014).

### Ectomycorrhizal Fungi

In terms of OTU richness and relative sequence abundance, Basidiomycota clearly dominated among EcM fungi (89.5% OTUs; 90.9% sequences; Supplementary Table S6). Out of 59 EcM fungal lineages and 2781 OTUs recovered (dataset#3), /tomentella-thelephora (22.2% of EcM fungal OTUs; 16.4% of sequences), /inocybe (18.2%; 22.8%), /cortinarius (13.2%; 7.3%), /russula-lactarius (7.4%; 14.5%), and /sebacina (5.4%; 5.8%) prevailed. Of exploration types – the presence, abundance and type of extraradical mycelium and rhizomorphs (Agerer, 2001) – OTUs with putatively short-distance-delicate type dominated (30.1% OTUs; 39.6% sequences), followed by medium-distance smooth (28.0%; 20.3%), medium-distance fringe (19.6%; 15.7%), contact (11.8%; 17.4%), short-distance coarse (6.2%; 5.3%), long-distance (2.5%, 1.2%) and mat (1.8%; 0.2%) types.

On average (dataset#4), each site harbored 93.0 ± 45.5 (mean ± SD) EcM fungal OTUs representing 20.0 ± 5.7 EcM lineages, a proxy for phylogenetic richness. Despite removing mold-infested samples (see methods; Supplementary Table S4), the abundance of Mortierellales (*R*^2^ = 0.240) and Pezizomycotina (*R*^2^ = 0.092) molds had a strong negative effect on relative abundance of EcM fungi. Mold abundance had a weaker effect on richness of EcM fungal OTUs (Mortierellales: *R*^2^ = 0.081) and lineages (Umbelopsidales: *R*^2^ = 0.050). The number of EcM fungal lineages responded strongest to the proportion of *Betula* spp. (positive effect; *R*^2^ = 0.189), *Quercus robur* (unimodal relationship, *R*^2^ = 0.111) and all EcM plants taken together (unimodal relationships, *R*^2^ = 0.064) as well as soil Ca concentration (positive effect; *R*^2^ = 0.111), pH (unimodal relationship; *R*^2^ = 0.062), richness of EcM plants (positive effect; *R*^2^ = 0.023) and vegetation age (lag at 140 years; *R*^2^ = 0.059).

OTU richness of individual 50 most common EcM fungal lineages (dataset#4) was best predicted by soil pH (30% of cases), followed by soil δ^15^N (12%), C/N ratio (10%, all positive effect), vegetation age (6%) or relative abundance of certain tree genus or taxonomic group (36%). Of other predictors, soil Mg concentration, P concentration and Ca concentration affected strongest the richness of/ceratobasidium2, /clavulina and /marcelleina-peziza gerardii lineages, respectively (Supplementary Table S7 and Supplementary Figure S5). The pH effect was positive in four Pezizales lineages and three basidiomycete lineages, whereas unimodal response occurred in six Basidiomycota lineages and negative effects were evident in/amanita and/hydnotrya. Preferences for tree genera had some unexpected patterns not known from previous research. The/geopora (*R*^2^ = 0.423), /pulvinula (*R*^2^ = 0.334), /pustularia (*R*^2^ = 0.137), /serendipita1 (*R*^2^ = 0.128) and /sordariales2 (*R*^2^ = 0.158) lineages were distinctly most diverse in various habitats dominated by *Salix* spp.; the /amphinema-tylospora (*R*^2^ = 0.574), /hygrophorus (*R*^2^ = 0.106), /pseudotomentella (0.132) and /rhodoscypha (*R*^2^ = 0.178) lineages prevailed in *Picea abies* forests; the /genea-humaria (*R*^2^ = 0.175), /otidea (*R*^2^ = 0.095) and/pisolithus-scleroderma (*R*^2^ = 0.239) peaked in diversity in *Quercus robur* habitats. *Pinus sylvestris* had a positive effect on the richness of the /suillus-rhizopogon (*R*^2^ = 0.213) and /hydnellum-sarcodon (*R*^2^ = 0.108) lineages, whereas *Corylus avellana* and *Tilia* spp. promoted diversity of the /pachyphloeus-amylascus (*R*^2^ = 0.064) and /entoloma (*R*^2^ = 0.020) lineages, respectively. In addition, the richness of /hysterangium (*R*^2^ = 0.028) and /tulasnella1 (*R*^2^ = 0.039) lineages responded strongest to plant phylogenetic vectors representing Pinaceae + Fagales and *Tilia* + Fagales, respectively (Supplementary Table S7). Richness of multiple EcM fungal lineages was positively affected by residual effects of relative abundance of any native EcM plant genus except *Alnus*. Composition of EcM fungal lineages was driven by soil pH (near-linear effect; *R*^2^ = 0.230), proportion of *Picea abies* (near-linear; *R*^2^ = 0.047), as well as soil C/N ratio (near-cumulative; *R*^2^ = 0.024) and δ^15^N (exponential rise; *R*^2^ = 0.008; Supplementary Figure S6).

Somewhat unexpectedly, relative abundances of EcM exploration types responded poorly to soil nutrients, with strongest effects of pH instead. Soil pH had a positive effect on short-distance-delicate type (*R*^2^ = 0.447) but a negative effect on contact type (*R*^2^ = 0.299). Soil δ^15^N had a negative effect on medium-distance-fringe type (*R*^2^ = 0.275), whereas medium-distance-smooth type was most abundant at medium values of soil C/N ratio (*R*^2^ = 0.140). Long-distance type increased linearly with vegetation age (*R*^2^ = 0.123), but mat type displayed negligible response to any environmental variables. Distribution of exploration types in NMDS ordination space relative to other variables is given in Supplementary Figure S6.

The lineage-level taxonomic composition of EcM fungi is consistent with previous comprehensive root-tip-based surveys in temperate Eurasia and North America, where forests of Pinaceae or Fagales dominate (Tedersoo et al., 2012a; Miyamoto et al., 2018; van der Linde et al., 2018). The overall commonness of/inocybe lineage and low abundance of/cortinarius, the rhizomorph-forming group and most species-rich EcM fungal genus in the Nordic countries (Knudsen and Vesterhold, 2012), is somewhat unexpected. Sequence-based dominance of/inocybe belowground and its large abundance of OTUs certainly indicate that this genus is more species-rich than expected, perhaps on the account of multiple cryptic species that we observed particularly for *Inocybe maculata* and *I. rimosa* species complexes (Larsson et al., 2009). The richness of/cortinarius but also/hebeloma-alnicola lineages may be severely underestimated by the chosen 98% sequence similarity threshold, as multiple closely related species in these groups differ by only a few bases across the entire ITS region (Schoch et al., 2012; Garnica et al., 2016; Grilli et al., 2016).

Exploration types of EcM have been viewed as ways of obtaining nutrients and plant carbon investments into mineral nutrition by differences in soil exploration, potential enzymatic activities and access to nutrients in different forms (Agerer, 2001; Jones et al., 2010; Tedersoo et al., 2012b). Across various ecosystems, our analyses revealed that relative abundance of only medium-distance-fringe and medium-distance-smooth types are related to ^15^N abundance or C/N ratio, whereas other types were unresponsive or responded to soil pH. Although pH affects nutrient availability, the distribution of exploration types may simply reflect that of dominant EcM fungal genera (e.g., *Russula* for contact type) and entire lineages (e.g., /tomentella-thelephora for medium-distance-smooth type), because the major morphological features of mycelium and rhizomorphs vary little at these taxonomic levels, being phylogenetically conserved. Hence, the confounding effects of phylogeny and exploration type need to be carefully disentangled.

### Structure of Fungal Communities

**FIGURE 8:**
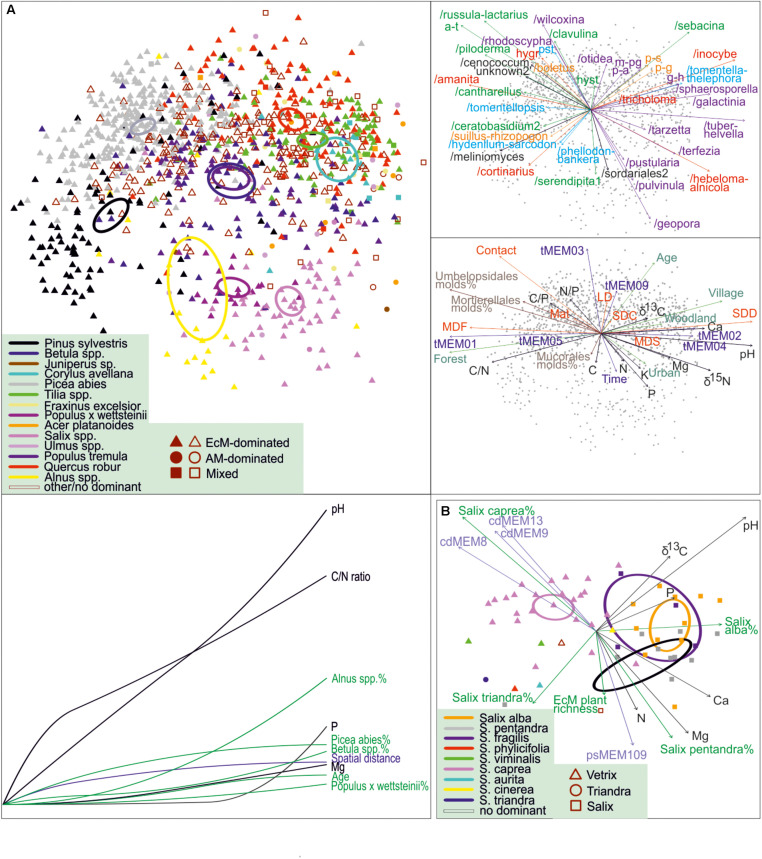
NMDS and GDM community profiles of ectomycorrhizal fungi across **(A)** all sites and **(B)** *Salix*-dominated sites. Circles, 95% ordiellipses for tree taxa; arrows, fit with environmental variables and placement of EcM fungal lineages and exploration types in top-right pane (a-t, /amphinema-tylospora; hygr, g-h, /genea-humaria; /hygrophorus; m-pg, /marcelleina-peziza gerardii; p-a, /pachyphloeus-amylascus; p-g, /paxillus-gyrodon; p-s, /pisolithus-scleroderma; pst, /pseudotomentella; colors represent lineages belonging to different orders or groups of orders) and mid-right pane (LDD, long-distance; MDF, medium-distance fringe; MDS, medium-distance smooth; SDC, short-distance coarse; SDD, short-distance delicate; colors depict different categories of variables), respectively. Bottom-left pane of **(A)** indicates the cumulative effect of environmental variables in the relative scale of minimum (left) to maximum (right) values as revealed from GDM. In **(B)**, symbols and their colors indicate different subgenera and species of *Salix*, respectively; cdMEMs and psMEMs represent phylogenetic eigenvectors indicating phylogeny effects on composition.

Multivariate PERMANOVA modeling revealed that community structure of saprotrophs, pathogens (both dataset#3) and EcM fungi (dataset#4a) was most strongly driven by soil pH, which explained 11.4, 6.4, and 5.3% of variation, respectively (Supplementary Table S8). The proportion of *Picea abies*, soil P and Mg concentrations had consistently significant effects on composition of fungal guilds, but these variables explained only 1.2–2.5% and 0.8–1.6% and 0.4–0.8% of variation, respectively. General dissimilarity modeling (GDM), which accounts for non-linearity in continuous variables, supported these results, but suggested relatively stronger complementary effects of soil C/N ratio, δ^15^N and Mg concentration to the pH effect (Figure 8A and Supplementary Figure S7). While the relative effect strength of soil pH was nearly linear for composition of saprotrophs and EcM fungi, it was cumulative for plant pathogens, indicating lower importance at near-neutral soils. Soil δ^15^N and C/N ratio effects were generally cumulative, suggesting that low N availability and N occurrence in mostly organic form are relatively more influential. Conversely, the effect strength of soil P and cation concentrations increased exponentially, indicating relatively stronger effects of surplus nutrients (cropland and urban habitats). Alternatively, these patterns may reflect an interaction of nutrients with soil pH, which determines their mobility and availability. The effect strength of tree species ranged from cumulative to linear to exponential, with a pronounced exponential effect of *Alnus* spp. on EcM fungal composition (Figure 8A). This is in agreement with specialized communities of *Alnus* spp. relative to other host plants, which become more prominent in monospecific stands (Kennedy et al., 2015). Conversely, cumulative effects can be interpreted as initial positive effects of critical abundance of a tree species or nutrient level until a certain threshold, beyond which further increase has a negligible effect.

As we obtained samples from multiple monospecific and mixed stands of various *Salix* species, we tested the effect of host, their phylogenetic associations and other environmental predictors on EcM fungal composition for *Salix*-dominated (relative abundance > 66%) data subset (*n* = 60). The best model suggested that *Salix* species, their phylogenetic relationships, soil pH, other soil variables and other vegetation variables explain 9.5% (*F*_2,48_ = 8.2), 2.4% (*F*_3,48_ = 3.2), 6.2% (*F*_1,48_ = 6.9), 2.5% (*F*_3,48_ = 3.1), and 2.1% (*F*_2,48_ = 3.8) of variation, respectively. The NMDS ordination indicates that the main differences occur between *Salix* subgenus *Vetrix* (*S. caprea*) and subgenus *Salix*, with no clear differences among *S. alba*, *S. pentandra* and *S. fragilis* belonging to the latter group (Figure 8B). Our analyses suggest that although the overall EcM fungal composition is driven by soil pH, individual tree genera and subgenera (in *Salix*) have a strong residual effect. We confirmed the effect of phylogenetic relatedness within the genus *Salix* but not at higher taxonomic levels (Tedersoo et al., 2013). Ordination graphs show that genera of Pinaceae and Fagales occur in separate positions, with non-overlapping confidence intervals (Figure 8A).

Our analyses indicate that soil pH is the main driver of composition of different fungal guilds, followed by the proportion of *Picea abies*. This uniform residual effect of one tree species across fungal guilds is unexpected, suggesting its effect on soil properties other than pH or a unique biotic environment through direct interactions with EcM fungi, saprotrophs and pathogens. These non-exclusive, perhaps synergistic mechanisms are plausible based on mycological literature (Põlme et al., 2018; Wang et al., 2019).

### Environmental Niche for OTUs

We modeled the realized environmental niche for 50 most frequent OTUs of EcM fungi and 50 non-EcM fungi. Relative abundance of EcM fungal OTUs was mainly driven by soil pH (28 cases), including 15 unimodal and 10 positive relationships (Supplementary Table S9 and Supplementary Figure S8). Proportion of a tree host was the best predictor for 16 OTUs, all with positive effects. Of these, nine OTUs (mostly species of *Amphinema*, *Tylospora*, and *Tricharina*) were strongly associated with *Picea abies*, whereas proportion of *Salix* spp. predicted relative abundance of OTUs corresponding to *Hyaloscypha finlandica*, *Tuber* sp. and *Cortinarius decipiens*. Vegetation age was the strongest predictor for three OTUs, with negative effects on relative abundance of *Cortinarius diasemospermus* and two OTUs corresponding to *Hyaloscypha finlandica* species complex. Relative abundance of non-EcM OTUs was mainly determined by soil pH (36 cases), with positive (17) and unimodal relationships (12) prevailing (Supplementary Table S10 and Supplementary Figure S9). Nine OTUs responded strongest to relative abundances of tree genera, mostly *Picea* (3) and *Pinus* (2). Furthermore, two non-EcM OTUs displayed strong preferences for non-forest or park habitats. Among the dominant OTUs, there were almost no taxa that occurred in all habitats or in all types of forests indicating a certain level of niche differentiation. Congeneric OTUs responded similarly to soil pH but similarly (*Amphinema* spp.) or differently (*Inocybe* spp., *Hyaloscypha* spp.) to host plants, suggesting that abiotic niche is more conserved than biotic niche within genera.

To evaluate the validity of clustering, we assessed whether OTUs of *Hyaloscypha finlandica-H. bicolor* species complex have differentiated niches or represent potentially the same biological/ecological species with high marker sequence variation, by combining phylogenetic analyses, niche modeling and co-occurrence analysis (legend to Supplementary Figure S10). Phylogenetic analyses resolved OTUs belonging to the *H. bicolor-H. finlandica* complex poorly, except that OTU00823 branched at the base of the complex and OTUs corresponding to *H. finlandica* formed a monophyletic group within *H. bicolor* that remained paraphyletic. OTUs of *H. bicolor* featured similar preference to low soil pH, whereas OTUs of *H. finlandica* responded negatively to vegetation age but variably to soil pH and host plants such as *Salix*, *Populus* and *Picea*. OTUs of both groups displayed negative (and neutral in *H. finlandica*) within-group co-occurrence patterns. Surprisingly, several interspecific pairs of OTUs displayed strongly positive co-occurrence values (Supplementary Figure S10).

The niche analysis shows that OTUs of *H. bicolor* have similar ecological preferences to low pH, whereas OTUs of *H. finlandica* are typically pioneer species with broad pH tolerance and unexpected host preference. In spite of some ecological niche overlap, the C-score analysis revealed no positive co-occurrence (Supplementary Figure S10), suggesting that the closely related OTUs avoid each other, possibly by competition (*H. bicolor*) or both competition and niche differentiation (*H. finlandica*). The presence of some strongly positive co-occurrences among more distantly related OTUs suggests that cryptic species of *H. bicolor* and *H. finlandica* are not strongly competing with each other when they co-occur.

Taken together, the OTU-specific niche analysis provides evidence that several closely related OTUs matching the same species can respond differently to environmental predictors and hence be ecologically relevant. However, this may differ by taxonomic groups and our ability to define the niche in a multivariate space. We used 98% ITS sequence similarity threshold for OTU clustering, but the optimal, biologically most relevant thresholds depend on genera and particular species complexes. It is likely that these ecologically distinct, co-occurrence-avoiding sympatric OTUs correspond to biological species or groups of species, but biological species need to be determined based on mating tests or phylogenetic concordance of multiple genes in fungi (Taylor et al., 2006).

### Habitat Type and Land Use

**FIGURE 9:**
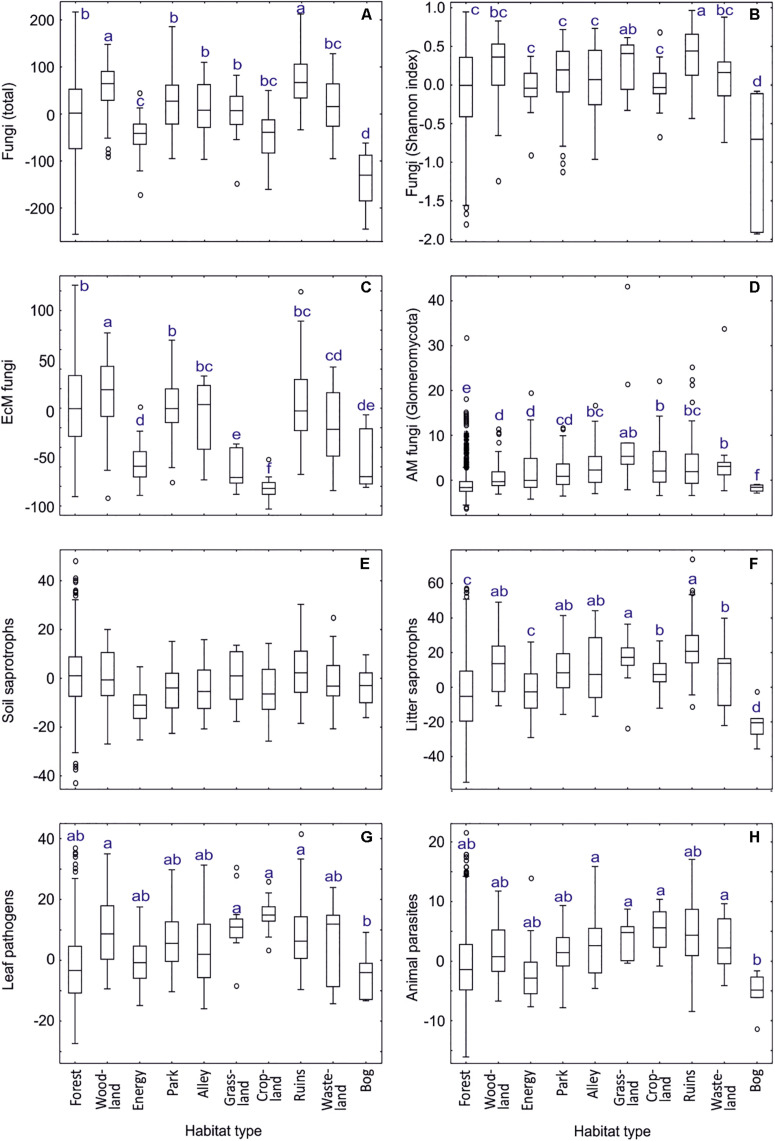
Habitat type effects on residual richness of fungi: **(A)** all fungi, **(B)** fungi Shannon index, **(C)** ectomycorrhizal fungi, **(D)** arbuscular mycorrhizal fungi, **(E)** soil saprotrophs, **(F)** litter saprotrophs, **(G)** leaf pathogens; **(H)** animal parasites. Middle lines, boxes, whiskers and circles represent medians, quartiles, 90% quantiles and outliers, respectively. Different letters above whiskers indicate statistically different groups.

Our analyses of habitat type and land use effects (dataset#3; for definitions, see Supplementary Table S2) revealed that fungal richness and Shannon index were highest around ruins and in woodlands but distinctly lowest in bogs (Supplementary Table S11 and Figure 9). EcM fungi were most diverse in woodlands, followed by forests, parks and ruins, with lowest richness in croplands, grasslands, energy plantations and bogs. Conversely, AM fungi prevailed in grasslands, followed by croplands and wastelands, being almost absent from bogs. Root endophytes were, however, most diverse in bogs, followed by forests. Dung saprotrophs prevailed in grasslands, with distinctly lowest values in bogs and forests. Litter, wood and white-rot decomposers were most diverse around ruins.

Unicellular fungi such as Aphelidiomycota, Monoblepharomycota, Neocallimastigomycota, Rozellomycota but also Zoopagomycota and unclassified fungi displayed greatest richness in energy plantations, whereas Spizellomycetes prevailed in croplands (Supplementary Table S11). Energy plantations were diversity hotspots also for Pleosporales and non-EcM Sebacinales. Croplands and grasslands were among the most preferred habitats for Zoopagomycota, Diversisporales, Glomerales, Pleosporales, Onygenales, Glomerellales, Hypocreales, Microascales, Sordariales, Auriculariales, and Ceratobasidiaceae. Ruins constituted richness hotspots for Helotiales, Thelebolales, Pezizales, Glomerellales, Xylariales and non-EcM Agaricales. Notably, diversity of only Hymenochaetales and Trechisporales peaked in forest habitats. Bogs represented the habitats of lowest richness for most fungal taxonomic groups, with croplands (Helotiales), energy plantations (Auriculariales, Trechisporales), wastelands (Monoblepharomycota, Hymenochaetales) and alleys (Zoopagomycota, non-EcM Sebacinales) also recorded as such for certain groups.

In EcM fungi (dataset#4), the overall phylogenetic diversity (richness of lineages) was comparable across habitat types, with lowest values in energy plantations (Supplementary Table S11). Forests harbored the greatest richness for /amanita, /amphinema-tylospora, /cantharellus, /clavulina, /cortinarius, /hygrophorus, /piloderma, /pseudotomentella, /rhodoscypha, /russula-lactarius, and /wilcoxina lineages. Woodlands were the preferred habitats for/cenococcum, /genea-humaria, /otidea, /sebacina, /tomentella-thelephora, and /tricholoma lineages. Parks or alleys represented richness hotspots for /galactinia, /geopora, /inocybe, /pisolithus-scleroderma, /sphaerosporella, /terfezia-peziza depressa and /tuber-helvella lineages. Surprisingly, energy plantations had greatest richness of the /hebeloma-alnicola, /laccaria, /meliniomyces and /pulvinula lineages, many of which are recognized as pioneer taxa (Vlk et al., 2020).

We attribute the differences in habitat types mostly to substantial differences in soil pH, δ^15^N and C/N ratio but also to tree community, particularly EcM vegetation (Supplementary Table S11). Woodlands and surroundings of ruins typically harbor a variety of microhabitats, which differ in pH and plant growth form, and high plant diversity that all favor high fungal richness. Energy plantations (*P. x wettsteinii* and *Salix viminalis*) were prominent for supporting high diversity of early diverging fungal lineages and pioneer groups of EcM fungi, which can be linked to high productivity and age effects, respectively. Similarly, the low richness of most fungal groups in bogs may be related to both low productivity and anaerobic conditions (Hiiesalu et al., 2017).

### Anthropogenic Impact

**FIGURE 10:**
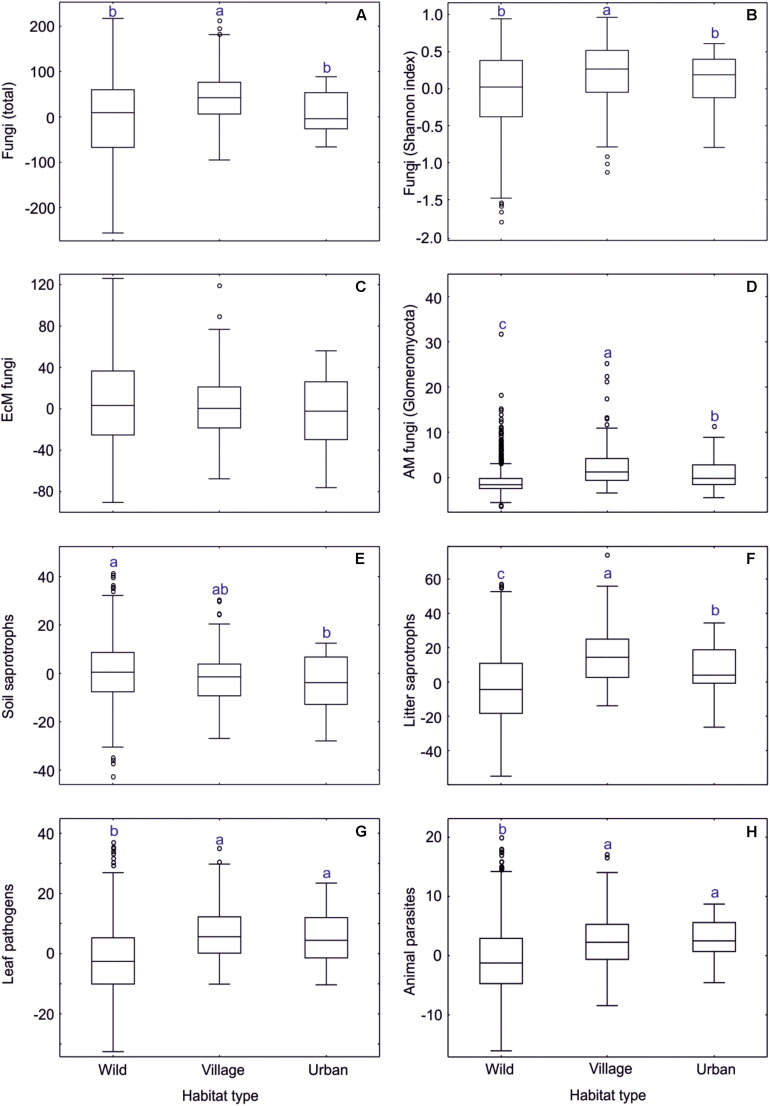
Anthropogenic impact on residual richness of fungi and major functional guilds. In box plots, central lines, boxes, whiskers and circles represent medians, quartiles, 90% quantiles and outliers, respectively; letters above whiskers depict significantly different groups. **(A)** all fungi; **(B)** all fungi (Shannon index); **(C)** EcM fungi; **(D)** AM fungi; **(E)** soil saprotrophs; **(F)** litter saprotrophs; **(G)** leaf pathogens; **(H)** animal parasites.

Based on a data subset of tree-dominated habitats (dataset#5), we evaluated the effect of overall anthropogenic impact on soil microbiome. Fungal richness and Shannon index were highest in the village biome, intermediate in the urban biome and lowest in wild tree-dominated habitats combined (Supplementary Table S12 and Figure 10). This pattern was also evident in AM fungi, leaf pathogens, opportunistic human pathogens as well as dung, litter, and wood saprotrophs. Animal parasites were slightly more common in urban biomes than village biomes, whereas root endophytes prevailed in forest habitats, followed by village and urban biomes. Of taxonomic groups, wild habitats were preferred only by a few groups including Umbelopsidomycetes, Endogonomycetes, Hymenochaetales, and Trechisporales. Other taxonomic groups were most diverse in village (Pezizales, Glomerellales, Hypocreales, Microascales, Sordariales, Xylariales, Ceratobasidiaceae, Rhizophlyctidomycetes, Spizellomycetes, Glomerales) or urban (Pleosporales, Onygenales, Cystofilobasidiales, unclassified fungi) biomes, usually with no strong difference between these (except Glomerales and Rhizophlyctidomycetes). Most EcM fungal lineages peaked in richness in wild habitats (/amanita, /amphinema-tylospora, /cantharellus, /cenococcum, /cortinarius, /hygrophorus, /meliniomyces, /piloderma, /russula-lactarius, /wilcoxina), whereas others were more diverse in village (/hebeloma-alnicola, /pisolithus-scleroderma, /sphaerosporella) or urban (/galactinia, /geopora, /terfezia-peziza depressa, /tuber-helvella) habitats, typically with no differences among the latter (except/geopora, and /terfezia-peziza depressa). Other functional and taxonomic groups and EcM fungal lineages displayed no biologically meaningful differences among these habitat types.

Wild, village and urban tree-dominated habitats differed in soil properties but also in tree composition, tree richness and overall EcM plant proportion (Supplementary Table S12). In particular, the village biome was associated with higher pH and δ^15^N as well as greater tree diversity than other biomes, which all have a positive effect on diversity of fungi and most groups therein. The relatively lower fungal diversity in forest habitats compared to village habitats is attributable to the low pH and low tree diversity of coniferous forests. Because the dummy variables of anthropogenic influence did not contribute to the best richness models, we conclude that the direct human impact on fungal diversity is generally low in natural and seminatural habitats in the anthropogenic gradient. Other authors have reported shifts in fungal community composition when including heavily polluted urban sites in this gradient (Timonen and Kauppinen, 2008; Jumpponen et al., 2010).

### Island Ecosystems and Habitat Fragmentation

**FIGURE 11:**
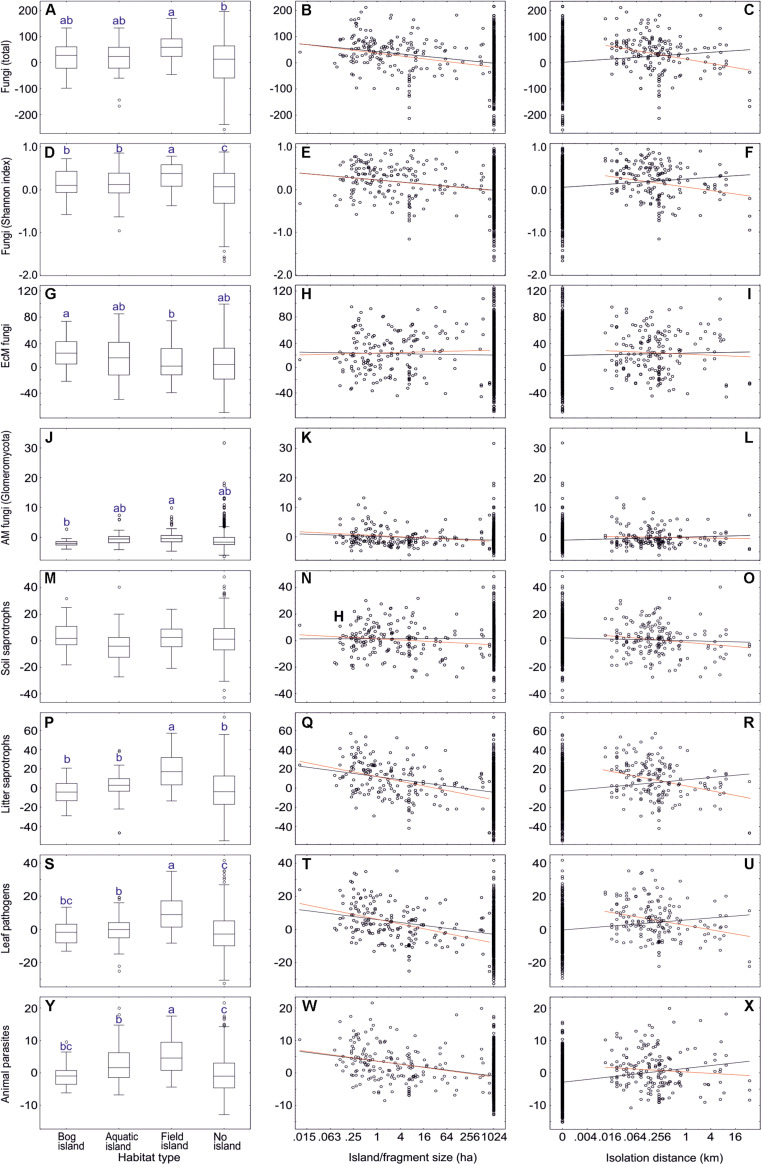
The effect of island habitat types (left panes), fragment size (middle panes) and isolation (right panes) on residual richness of fungi and major functional guilds: **(A–C)** all fungi; **(D–F)** all fungi (Shannon index); **(G–I)** ectomycorrhizal fungi; **(J–L)** AM fungi; **(M–O)** soil saprotrophs; **(P–R)** litter saprotrophs; **(S–U)** plant pathogens; **(V–X)** animal parasites. In box plots, central lines, boxes, whiskers and circles represent medians, quartiles, 90% quantiles and outliers, respectively; letters above whiskers depict significantly different groups. In scatter plots, black and red lines respectively indicate linear regression lines when including and excluding non-fragmented habitats. Note that fragments larger than 1024 ha were not precisely measured and truncated to 1024 ha. Note the base-2 logarithmic scale in x-axis of scatter plots.

To test the effect of forest fragmentation including natural and anthropogenic islands on soil biodiversity, we excluded the following plots: non-forest habitat, vegetation age < 30 years, EcM plant basal area < 30%, selective harvesting < 5 years ago. This dataset#6 comprised 784 samples including 69 forest fragments and 107 islands (<20 km^2^), which were categorized into aquatic islands (*n* = 37), bog islands (27) and field-surrounded islands (43). Island type and island size usually had a weak but significant effect on biodiversity of one third of the tested fungal groups. Contrary to our hypothesis, Shannon index and richness of fungi and most fungal groups were greater in island ecosystems than non-island ecosystems; in a majority of these cases, increasing island or forest fragment size had a negative effect on diversity (Supplementary Table S13 and Figure 11). For many groups, there was also a meaningful island type × patch size effect, indicating that size matters depending on the island type. Richness and Shannon index of fungi and most fungal groups were highest in field islands (64% of cases) but lowest in non-islands or bog islands (for example, AM fungi, various guilds of pathogens and saprotrophs, taxonomic groups of non-ectomycorrhizal fungi). Conversely, root endophytes, Hymenochaetales, Umbelopsidomycetes and Endogonomycetes displayed greater diversity in bog islands and non-islands compared with field islands.

Unlike most other fungal groups, EcM fungal richness did not differ among island types and non-islands, and there was no effect of island size or distance. In spite of this, EcM fungal lineages differed strongly in their preferences for specific island types and island size (Supplementary Table S13). While their preference for island type reflected their pH optima, their response to island size largely coincided with their inferred pioneer vs. competitor strategy. Groups with multiple pioneer species such as pezizalean lineages, /pisolithus-scleroderma and /tomentella-thelephora were relatively more abundant and diverse on smaller islands. Conversely, the /russula-lactarius, /piloderma, /clavulina, /cenococcum, /cantharellus, /amanita, and /amphinema-tylospora lineages were more common in large islands and non-islands (Supplementary Table S13).

Non-mycorrhizal fungi responded relatively more strongly to island size. Dung saprotrophs (*R*^2^ = 0.083), Pleosporales (*R*^2^ = 0.099), Glomerellales (*R*^2^ = 0.081) and Sordariales (*R*^2^ = 0.093) were most diverse on small islands. The only groups that preferred larger islands and non-islands were root endophytes, as well as Hymenochaetales, Umbelopsidomycetes and Endogonomycetes. In both EcM and non-EcM fungi, preference for field islands generally coincided with higher diversity in smaller islands, whereas preference for bog islands tended to reflect greater richness in larger islands and non-islands.

Excluding plots from non-island and non-fragmented habitats, islanddistance and distance × island type affected only a few fungal groups (Supplementary Table S13 and Figure 11). With increasing isolation, richness of total fungi, litter saprotrophs, wood saprotrophs, Capnodiales and Xylariales declined. The EcM fungal lineages /genea-humaria, /sebacina and /tomentella-thelephora were also less diverse in more isolated islands. In other guilds and taxonomic groups, reduction in diversity was statistically marginally significant or the effect was absent; no significant increases occurred.

In forest fragments of non-island ecosystems (dataset#7), isolation distance had somewhat weaker trends on richness of isolation-sensitive fungal groups. The strongest negative effects of forest fragment distance occurred in the EcM fungal lineages /russula-lactarius (*R*^2^ = 0.116; *P* = 0.003) and /tricholoma (*R*^2^ = 0.067; *P* = 0.047).

The tendency for greater fungal richness in islands, particularly small islands, is in disagreement with previous work that demonstrated positive effect (Peay et al., 2010) or no effect (Li et al., 2020) of island size. The high diversity in small islands can be attributable to edge effect – a non-linear gradient of environmental conditions that extends up to 50 m in the habitat interior – which promotes overall habitat heterogeneity and niches (Crockatt, 2012). However, previous studies revealed decline in mycelium biomass, activity, reproductive effort and aboveground diversity toward the ecotone (Crockatt, 2012; Grilli et al., 2017). Our large plots cover various habitat conditions in the ecotone and interior of islands, increasing the number of sampled niches. We attribute the relatively low diversity of fungi in bog islands to low pH in these habitats, because the fungal groups that responded to bog island habitat coincided with groups most strongly driven by soil pH. Besides pH, island types and islands of varying size differed most strongly in soil C/P ratio, δ^15^N and relative basal area of *Picea abies* and *Salix* spp. (Supplementary Table S13) that are important predictors for many groups of fungi.

Fungal richness declined with increasing island distance, corroborating the results of Peay et al. (2010) in small tree islands separated by shrubs but not in a system of larger aquatic islands in China (Li et al., 2020). Of individual groups, representatives of both asexual microfungi and truffle-like and corticioid fruit-body formers had significant dispersal limitation, indicating no striking overall differences among dispersal strategies. Forest fragments displayed somewhat weaker island biogeography patterns compared with islands, but this is probably related to more recent history of isolation and short evolution as an island ecosystem (decades vs. centuries or millennia). Our results confirm that forest fragmentation reduces richness of certain poorly dispersed and old forest specialist groups (Grilli et al., 2017).

### Old and Virgin Forests

Although vegetation age was an important predictor for several fungal groups, the effect usually leveled off at 80–140 years. Therefore, we tested if tree age *per se* and virgin conditions in particular affect diversity patterns in mature forest ecosystems. We separated forest plots aged > 100 years into virgin (*n* = 34) and non-virgin (*n* = 421) habitats (dataset#8) based on literature and expert evaluation. Virgin conditions and increasing age in >100-years old stands had weak but positive effects on richness of most fungal taxonomic and functional groups (including total fungal richness and Shannon index and EcM fungal richness), with or without accounting for other predictors (Supplementary Table S14). Both old-growth conditions (*R*^2^ = 0.029) and age (*R*^2^ = 0.016) promoted richness of white-rot decomposers. Similarly, richness of Capnodiales was greater in virgin forests (*R*^2^ = 0.056), increasing with age (*R*^2^ = 0.018). Virgin forests harbored greater richness of three major agaricomycete decomposers – Hymenochaetales (*R*^2^ = 0.027), Polyporales (*R*^2^ = 0.029) and Trechisporales (*R*^2^ = 0.032), without a residual age effect. In EcM fungal lineages, we observed greater richness for /amphinema-tylospora (*R*^2^ = 0.065), /cortinarius (*R*^2^ = 0.026) and /piloderma (*R*^2^ = 0.043) lineages in virgin forests. Considering habitat conditions, virgin forests exhibited greater amounts of topsoil C (*R*^2^ = 0.047) and K (*R*^2^ = 0.059) and proportion of *Picea abies* (*R*^2^ = 0.043) but lower δ^15^N values (*R*^2^ = 0.049). With age increase from 100 to 300 years, there was a slight increase in topsoil C (*R*^2^ = 0.023) and K (*R*^2^ = 0.023) concentrations and proportion of *Quercus robur* (*R*^2^ = 0.063; Supplementary Table S14).

Virgin forests harbor large amounts of decaying wood in various stages of decomposition and develop gaps that are suitable for establishment of pioneer species (Harmon et al., 1986). Richness increase in white-rot decomposers and corresponding Hymenochaetales, Trechisporales and Polyporales (partly brown-rotters) orders probably benefit from greater amounts of woody residues that are buried in soil. Many wood-inhabiting species of Hymenochaetales and Polyporales are red-listed and considered indicators of primeval habitats (Lõhmus et al., 2018). As described above, diversity of Hymenochaetales and Trechisporales declined in island habitats, suggesting that a combination of limited dispersal capacity and preference for virgin forests renders these taxa particularly vulnerable to habitat loss and fragmentation.

### Forest Management

**FIGURE 12:**
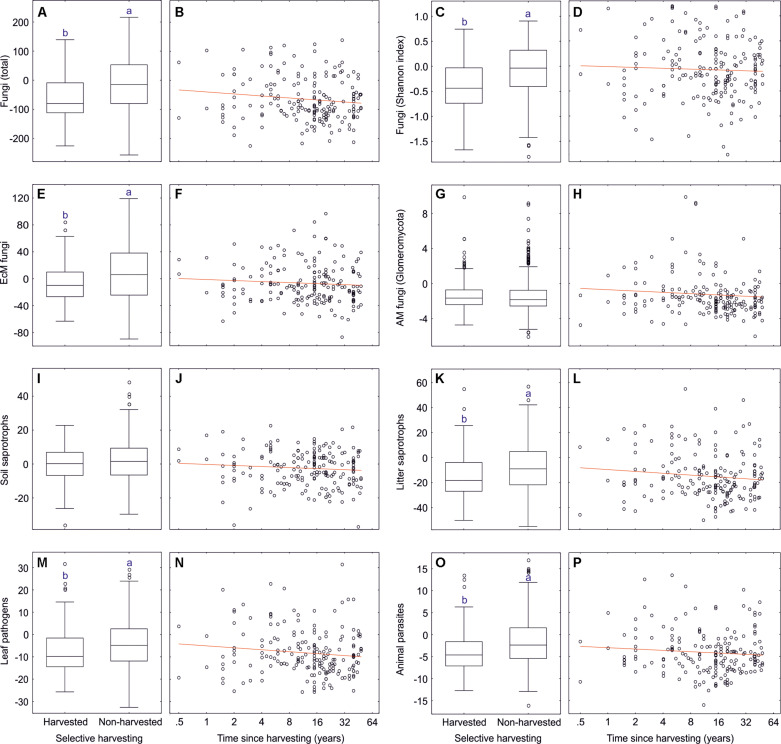
Effects of selective harvesting or thinning and time since cutting on richness of fungi and major fungal guilds: **(A,B)** all fungi; **(C,D)** all fungi (Shannon index); **(E,F)** ectomycorrhizal fungi; **(G,H)** AM fungi; **(I,J)** soil saprotrophs; **(K,L)** litter saprotrophs; **(M,N)** plant pathogens; **(O,P)** animal parasites. Middle lines, boxes, whiskers and circles represent medians, quartiles, 90% quantiles and outliers, respectively. Different letters above whiskers indicate statistically different groups. In scatter plots, red lines indicate linear regression lines.

We addressed the effects of selective harvesting (thinning or selective removal of <50% basal area, excluding clear-cuts) and time since harvesting on soil microbiome by subsampling forest plots dominated by *Picea abies*, *Pinus sylvestris*, *Betula pendula* and *Quercus robur* aged 9–100 years (dataset#9: 111 harvested plots, 646 unharvested plots). The overall selective harvesting effect was neutral to slightly negative on most fungal taxonomic and functional groups (Supplementary Table S15 and Figure 12), including fungal richness (*R*^2^ = 0.040) and Shannon index (*R*^2^ = 0.045) and EcM fungal richness (*R*^2^ = 0.017). Strongest negative responses to harvesting were observed for opportunistic human pathogens (*R*^2^ = 0.051), dimorphic yeasts (*R*^2^ = 0.049), yeasts (*R*^2^ = 0.042), and animal parasites (*R*^2^ = 0.029). These effects were generally weaker in fungal taxonomic groups and EcM fungal lineages. In contrast to most other lineages, richness of/wilcoxina (*R*^2^ = 0.056), /russula-lactarius (*R*^2^ = 0.035), /pseudotomentella (*R*^2^ = 0.027) and/amphinema-tylospora (*R*^2^ = 0.024) responded positively to selective harvesting. The strongest negative effects were observed for/cortinarius (*R*^2^ = 0.050). Of EcM exploration types, only contact type responded to harvesting (*R*^2^ = 0.039; positive effect), which could at least partly be ascribed to the increase in *Russula* spp.

Time since selective harvesting (*n* = 111 harvested plots) had no effect on fungal diversity and EcM fungal richness, but it had contrasting effects on selected functional and taxonomic groups and EcM fungal lineages, often exceeding the magnitude of harvesting effect *per se* (Supplementary Table S15). Richness of opportunistic human pathogens (*R*^2^ = 0.095), animal parasites (*R*^2^ = 0.057), white-rot decomposers (*R*^2^ = 0.056), wood saprotrophs (*R*^2^ = 0.047) and mycoparasites (*R*^2^ = 0.043) all declined with time since harvesting. This pattern was also characteristic to Hypocreales (*R*^2^ = 0.075), Eurotiales (*R*^2^ = 0.066), Glomerellales (*R*^2^ = 0.043) and several other groups. Conversely, Umbelopsidomycetes (*R*^2^ = 0.094) and non-EcM Sebacinales (*R*^2^ = 0.042) exhibited greater richness with increasing time after recovery. Richness of the EcM fungal lineages/amanita (*R*^2^ = 0.139), /russula-lactarius (*R*^2^ = 0.106), /suillus-rhizopogon (*R*^2^ = 0.059) and/piloderma (*R*^2^ = 0.044) increased with time since harvesting, whereas diversity of paxillus-gyrodon (*R*^2^ = 0.071), /hebeloma-alnicola (*R*^2^ = 0.064) and /tuber-helvella (*R*^2^ = 0.055) was greatest in recently harvested plots.

In selectively harvested plots, the decline in overall fungal richness was related to decrease in EcM fungi and yeasts. Our results are thus partly consistent with a study in Northern Sweden that found 30–40% decline in EcM fungal diversity in a few years after partial harvesting (Sterkenburg et al., 2019) but in a disagreement with studies in Spanish Mediterranean forests revealing no effect (Castano et al., 2018a; Parlade et al., 2019). However, our study corroborates the long-term selective harvesting effect on composition of fungi and specifically EcM fungi in Mediterranean forests, which was not evident in Sweden. Compared to 30–40% basal area harvesting in these studies 1–5 years ago, our plots were harvested on average at 23.8% intensity 10.2 years previously. These harvesting effects can be attributed to three main factors – (i) mechanical disturbance and disruption of EcM fungal networks; (ii) increase in fresh stumps and dying root systems; and (iii) short-term rise in soil pH – that all favor proliferation of opportunistic groups at the expense of EcM fungi (Lindahl et al., 2010). The observed shifts in EcM fungal composition may be related to altered competitive balance due to losses of the carbohydrate source and massive disturbance that favor establishment of species from genera with pioneer strategy such as *Hebeloma*, *Paxillus*, and *Tuber*. Relative abundance of contact exploration type, mostly representing *Russula* and *Tuber* spp., was positively related to harvesting, which points to an advantage of low C demanding groups after declining carbohydrate sources (Leake et al., 2004).

With increasing time since selective harvesting, shade-tolerant pioneer species such as *Sorbus aucuparia* and *Corylus avellana* and many other small bushes (Supplementary Table S15) establish from seed or stump sprouts, potentially contributing to recovery of fungal richness besides the effect of aging *per se*. However, full recovery of selectively harvested plots may take several decades or centuries if long-lived EcM tree species are replaced by long-lived AM trees (Chen J. et al., 2019). Even without major vegetation shifts, recovery of EcM fungi from a clear-cut state may take 90 years based on model averages (Spake et al., 2015). Correspondingly, our results from this and the virgin habitat analysis extend these findings, indicating that selective harvesting and thinning may affect fungal composition for multiple decades.

### Management of Parks and Woodlands

To maintain the semiopen structure, European woodlands and parks require consistent management by grazing or mowing and removal of coppicing sprouts. We tested whether management of seminatural woodlands and parks affects diversity of soil organisms (dataset#10: 40 wooded meadows, 17 wooded pastures, 100 parks). Wooded meadows showed higher fungal richness (*R*^2^ = 0.113) and Shannon index (*R*^2^ = 0.061) than pastures (Table 3). This pattern also occurred in soil saprotrophs (*R*^2^ = 0.101), especially Mortierellomycetes (*R*^2^ = 0.166) and Umbelopsidomycetes (*R*^2^ = 0.080) and Rhizophlyctidomycetes (*R*^2^ = 0.141), and non-significantly so for most fungal taxonomic and functional groups. Of EcM fungal lineages, /cortinarius (*R*^2^ = 0.213) and /russula-lactarius (*R*^2^ = 0.050) had substantially greater richness in wooded meadows than pastures, but this pattern was non-significant for the overall EcM fungal and lineage richness.

**TABLE 3:** Differences in parks, wooded pastures and wooded meadows and their management on fungal diversity and environmental parameters.

Dependent variables	Habitat type (*R*^2^,%)^1^	Habitat type effect (ranked)^2^	Management effect (*R*^2^,%)	Management effect (ranked)^3^	Habitat × management (*R*^2^,%)
Fungi richness	**11.3**	WM > park = WP	ns	ns	ns
Fungi Shannon index	**6.1**	WM > park = WP	ns	ns	ns
**Fungal functional groups**					
Ectomycorrhizal fungi	**7.0**	WM > WP > park	ns	ns	ns
Root endophytes	**6.0**	WM > park = WP	ns	ns	ns
Dung saprotrophs	ns	ns	**9.9**	M = UM > C	ns
Soil saprotrophs	**10.1**	WM > park = WP	ns	ns	ns
Leaf pathogens	ns	ns	**8.9**	M > UM > C	ns
**Fungal taxonomic groups**					
Pleosporales	ns	ns	**19.4**	M > UM = C	ns
Geoglossales	**10.8**	WP = EM > park	ns	ns	ns
Helotiales	ns	ns	**8.2**	C > M = UM	ns
Thelebolales	**9.1**	park = WM > WP	ns	ns	ns
Microascales	ns	ns	**11.3**	M = UM > C	ns
Sordariales	ns	ns	**11.1**	M = UM > C	ns
Agaricales (NM)	**19.4**	WM > WP > park	ns	ns	**8.5**
Auriculariales	**7.9**	WM = WP > park	ns	ns	ns
Ceratobasidiaceae	ns	ns	**5.6**	M > UM = C	ns
Geminibasidiomycetes	9.9	WP = WM > park	ns	ns	**9.3**
Cystofilobasidiales	6.7	park = WM > WP	ns	ns	ns
Rhizophlyctidomycetes	14.1	park > WM > WP	ns	ns	ns
Mortierellomycetes	16.6	park > WM > WP	ns	ns	ns
Mucoromycetes	ns	ns	**5.1**	M > UM = C	ns
Umbelopsidomycetes	**8.0**	WM > park = WP	3.6	C > M = UM	ns
Rozellomycota	ns	ns	**6.7**	C > UM = M	ns
**Lineages of EcM fungi**		ns	ns	ns	ns
/amphinema-tylospora	ns	ns	**12.1**	C > UM = M	ns
/cenococcum	**8.8**	WP > WM > park	ns	ns	ns
/cortinarius	**21.3**	WM > WP = park	3.5	C > UM = M	ns
/meliniomyces	**7.2**	WM > WP = park	**7.2**	C > M = UM	ns
/pisolithus-scleroderma	**6.3**	park > WP = WM	ns	ns	ns
/sebacina	**9.9**	WP > WM > park	ns	ns	ns
/tomentella-thelephora	16.0	WP = WM > park	ns	ns	ns
/tricholoma	18.2	WM = WP > park	ns	ns	ns
/tuber-helvella	12.5	park > WP > WM	ns	ns	ns
/wilcoxina	ns	ns	**8.6**	C > UM > M	ns
**Edaphic and floristic parameters**					
Soil carbon	**17.7**	WP > WM > park	ns	ns	ns
Soil phosphorus	**34.0**	park > WP = WM	ns	ns	ns
Soil N/P ratio	**47.0**	WM = WP > park	ns	ns	ns
Soil δ^15^N	**14.1**	park > WP = WM	ns	ns	ns
Plant richness	ns	ns	**20.6**	C > UM > M	ns
*Picea abies*%	ns	ns	**15.4**	C > UM > M	ns
*Tilia* spp.%	**25.2**	park > WM = WP	ns	ns	ns
*Ulmus glabra*%	5.6	park > WM = WP	**13.6**	C = UM > M	ns
*Prunus padus%*	ns	ns	**11.6**	C > UM = M	ns
*Sorbus aucuparia%*	**10.9**	WP > WM = park	**13.3**	C > UM = M	ns
*Acer platanoides%*	**24.1**	park > WM = WP	ns	ns	ns

*Values in bold are significant (P < 0.001). ^1^ns, not suggestive at P > 0.05; ^2^WM, wooded meadow; WP, wooded pasture; > / = / < indicate significant differences among groups; ^3^C, coppiced; M, managed; UM, unmanaged; > / = / < indicate significant differences among groups.*

Management of parks and woodlands had a biologically meaningful effect on some of the taxonomic and functional groups. Diversity of fungi including EcM fungi tended to be higher in coppiced habitats compared to unmanaged and managed habitats (Table 3). In particular, richness of wood pathogens (*R*^2^ = 0.044), Helotiales (*R*^2^ = 0.082), Rozellomycota (*R*^2^ = 0.067) and Umbelopsidomycetes (*R*^2^ = 0.036) was greatest in coppiced habitats. Conversely, richness of dung saprotrophs (*R*^2^ = 0.099), leaf pathogens (*R*^2^ = 0.089), Pleosporales (*R*^2^ = 0.194), Microascales (*R*^2^ = 0.113), Sordariales (*R*^2^ = 0.111), Ceratobasidiaceae (*R*^2^ = 0.056) and Mucoromycetes (*R*^2^ = 0.051) was greatest in managed habitats. Of EcM fungi, the lineages/amphinema-tylospora (*R*^2^ = 0.121), /wilcoxina (*R*^2^ = 0.087), /meliniomyces (*R*^2^ = 0.072), /piloderma (*R*^2^ = 0.043) and /cortinarius (*R*^2^ = 0.035) were more diverse in coppiced habitats, while none of the groups preferred managed or unmanaged sites. Unmanaged habitats displayed nearly always intermediate diversity between managed and coppiced habitats. The interaction term of habitat type and management was significant in only a few cases, indicating that coppicing has similar effects on fungi in parks and woodlands. Coppiced habitats had greater richness of woody plants (*R*^2^ = 0.206) including EcM hosts (*R*^2^ = 0.191) such as *Picea abies* (*R*^2^ = 0.154) but lower total proportion of EcM plants (*R*^2^ = 0.073; Table 3).

Greater woody plant richness, establishment of conifers along with several other tree species and lower mechanical disturbance probably favor richness of most fungal groups in coppiced habitats. Because of contrasting management effects on different taxonomic and functional groups, patch-wise management and restoration of parks and woodlands probably promotes greatest fungal richness in these ecosystems. Retention of as many tree species as possible will probably benefit fungal biodiversity most.

### The Prevailing pH Effect

One of the most remarkable findings of this study is the overwhelming effect of soil pH or its closely related variables (C/N ratio, δ^15^N, cation concentration) on the distribution of individual fungal OTUs, which collectively drive the observed patterns in diversity and composition of most guilds and higher taxa of fungi. While most groups had a distinct unimodal relationship with soil pH, many order-level taxa displayed preference for extreme pH values, suggesting phylogenetically conserved adaptation to such conditions (Rousk et al., 2010a) or the realized bimodal niche in yeasts, for example. The effect of pH is related to physiological capacity of organisms to tolerate high H^+^ or OH^–^ ions and these differ strongly across broad taxonomic groups (Rousk et al., 2010b). Overall, the strong effect of pH is characteristic to much of the soil microbiome, especially bacteria (Rousk et al., 2010a; Bahram et al., 2018). The average pH effect on non-EcM fungal OTUs (*R*^2^_*average*_ = 0.195), EcM fungal OTUs (*R*^2^_*average*_ = 0.081) and orders (*R*^2^_*average*_ = 0.120) somewhat exceeds the regional-scale pH effect on bacterial genera (*R*^2^_*average*_ = 0.144) and phyla (*R*^2^_*average*_ = 0.072) across France (pH range 3.7–9.2; Karimi et al., 2018). Our study therefore extends the high impact of soil pH on diversity of fungi (Rousk et al., 2010a; Glassman et al., 2017) and its functional and taxonomic groups by removing the confounding effects of latitude, climate and historical effects that prevail in global-scale studies (Tedersoo et al., 2014; Egidi et al., 2019; Vetrovsky et al., 2019; Oliverio et al., 2020). Based on extrapolated soil chemical data, Vetrovsky et al. (2019) found a relatively low soil pH effect on fungal diversity, indicating the importance of using empirically determined values.

Furthermore, our correlation analyses and SEMs suggest that soil pH affects fungal composition also indirectly via plant diversity and composition as well as proliferation of molds (Figure 7 and Supplementary Figure S11). EcM fungi and evergreen plants acidify soil by exuding organic acids and shedding recalcitrant litter (Cornelissen et al., 2001; Tedersoo and Bahram, 2019). Therefore, combination of certain tree species and bedrock type may result in extreme pH values that magnify the pH effect on soil microbiome. The extrapolated maps of soil mycobiome mostly follow habitat type and soil pH (Figure 2 and Supplementary Figures S12–S18). Soil pH also determines the solubility of minerals containing P and cations, and hence the availability of these nutrients to both microorganisms and plants. Apart from Ca and Mg concentration, we found only negligible effects of soil nutrient concentration on fungal richness, but the effects of interaction between nutrient concentration, δ^15^N and soil pH are difficult to test non-experimentally. Although soil Mg availability is important for plant chlorophyll production, stress resistance and intercellular transportation (Senbayram et al., 2016), little is known about its role in soil microbiome, providing no clear explanation for the prominent effect of soil Mg on richness of certain fungal taxa and ciliates (L. Tedersoo et al., unpublished).

### Tree Species Effects

Besides the indirect effects of soil pH, tree species and genera had strong additional effects on fungal diversity and composition, which sometimes prevailed in niche analysis of individual OTUs, particularly EcM taxa. Consistent with our hypothesis that biotrophic groups respond more strongly to host plants than saprotrophs, tree species had 27 and 52% stronger effects on composition of plant pathogens and EcM fungi compared with saprotrophs. In terms of richness, plant species did not affect the diversity of soil and dung saprotrophs or mycoparasites and animal parasites, but had a weak effect on litter (3.1% variation) and wood (4.4% variation) saprotrophs and leaf pathogens (4.4% variation) - guilds that are associated with decomposing or living plant material. Yet, individual host trees collectively explained 35.0% variation in EcM fungal diversity. Any specific host effects on AM fungi were probably masked by the dominant effect of EcM plant proportion and our focus on woody plants exclusively. Although AM fungi lack host specificity, many AM plants associate with greater diversity of fungi than others and display differences in community composition (Põlme et al., 2018; Sepp et al., 2019).

The importance of individual tree genera on fungal composition was unrelated to the frequency of these taxa and differed remarkably across both plant and fungal groups. *Pinus sylvestris* was always associated with the most divergent composition stretched out along the primary axis in NMDS plots, with *Picea abies* also in a separate position (Figure 8 and Supplementary Figure S7). The high diversity of several unicellular fungal groups and protists (L. Tedersoo et al., unpublished) in hybrid poplar energy plantations is unexpected, but it may be related to high primary productivity and perhaps well-developed herbaceous understorey. The effects of *Betula* spp. and *Populus tremula* on any fungal functional group were highly similar, although both host trees associate with a number of specific EcM, saprotrophic and pathogenic fungi. Perhaps due to the mull soils, *Corylus avellana* and *Tilia* spp. also shared much of the fungal composition and plots dominated by AM angiosperms (*Acer*, *Ulmus* and *Fraxinus* species) overlapped in fungal composition. However, the AM *Juniperus* was associated with highly distinct saprotroph communities, which was not observed for plant pathogens. Based on PERMANOVA analysis, other tree taxa harbored distinct EcM fungal communities, but similar communities of saprotrophs and pathogens. Although several studies have indicated strong grouping of closely related plant species in fungal composition (Põlme et al., 2013; Tedersoo et al., 2013; Wu et al., 2018; Wang et al., 2019; Yang et al., 2019), the lack of grouping of closely related tree genera reflects the overall low importance of tree phylogeny above genus level.

Taken together, most overstory tree species affect community composition of EcM fungi, but only a few have significant influence on other fungal guilds. At the plot scale, greater overall tree diversity promotes fungal richness either directly or indirectly through positive and unimodal effects of individual tree taxa. In particular, the EcM conifers *Picea abies* and *Pinus sylvestris* are associated with strongest effects on diversity and composition, with part of these effects attributable to soil acidification. Therefore, both of these conifers can be regarded as non-redundant keystone species in the temperate and boreal forest belt of North and Central Europe by generating unique habitats suitable for the specialist host-associated and acidophilic fungal communities. Although pure stands of conifers were associated with reduced diversity of several taxonomic and functional groups, their presence adds much to the landscape-scale soil microbial diversity.

### Methodological Implications

The universal eukaryotic primers enabled us to capture the full diversity of kingdom Fungi and other eukaryotes as well, but the relative sequence abundance and identification power for protists and animals was much lower because of high fungal biomass in soil and poorly managed reference data of non-fungal ITS sequences. For these groups, several-fold deeper sequencing or use of group-specific primers and rigorous development of reference databases would certainly benefit species-level biodiversity analyses using environmental DNA.

The 18S-ITS metabarcoding by third-generation sequencing allowed us to use the entire variable ITS region for identification, which represents a significant improvement compared to short ITS1 or ITS2 barcodes in terms of taxonomic resolution (Garnica et al., 2016) and precision of identification (Tedersoo et al., 2018b), reducing the proportion of unidentified taxa by an order of magnitude compared to studies using short amplicons (Tedersoo et al., 2016; Chen L. et al., 2019; Sterkenburg et al., 2019). The vast majority of the well-delimited, abundant fungal species were represented by a single OTU, which contrasts to the occurrence of multiple co-occurring sister OTUs as revealed by short-read metabarcoding or metagenomics using 454 or Illumina platforms (Tedersoo et al., 2015, 2016). The long barcode enables more accurate clustering, because random PCR and sequencing errors are less likely to accumulate over longer marker regions and sequences with a few errors are more easily incorporated into the main OTUs (Tedersoo et al., 2018b). Furthermore, the PacBio platform provides better quantitative output compared with the Illumina platform (Castano et al., 2020).

Our study represents perhaps the only oversampled microbiome investigation on a regional geographic scale, which is reflected by virtually stabilizing OTU accumulation curves (Figure 4). The high sample coverage enabled us to test for meager effects of variables (*R*^2^ = 0.006 and *R*^2^ = 0.002 correspond to *P* < 0.001 in GLM and PERMANOVA analyses, respectively), far beyond their potential biological importance. However, the leveling off rarefaction curves need to be interpreted in the context of sequence data filtering and similarity thresholds. By incorporating sequences into UNITE Species Hypotheses (Kõljalg et al., 2013), we observed that many global singletons, otherwise excluded from analyses, fell into existing UNITE SHs, representing truly rare species in the context of this study. For example, singletons included the only representative of the/acephala macrosclerotiorum EcM lineage and several species of *Tuber* and *Helvella*. However, most singletons were chimeric, of low-quality, or possessed > 5mer indels rarely observed in Sanger sequences. Based on the data set of Schoch et al. (2012), it is also clear that multiple biological species of fungi cannot be distinguished based on the 98% or even 100% ITS sequence similarity threshold, for example many groups in *Penicillium* and *Fusarium* (Seifert, 2009). Based on ITS metabarcoding alone, we cannot predict the diversity of these species-rich genera and the proportion of taxa that are missed or lumped with this approach. For the morphological species of *Hyaloscypha* with around 3% ITS sequence variation, we showed that OTUs at 98% similarity level are ecologically differentiated, but whether this is an optimal threshold or lumps together multiple other ecologically distinct OTUs, remains to be evaluated more broadly.

With respect to statistical analyses, PERMANOVA and GDM algorithms strongly complemented each other in multivariate analyses by recovering the major predictors and determining their value-dependent importance (Landesman et al., 2014; Glassman et al., 2017), respectively. Combining a machine learning algorithm for initial variable selection with quadratic GLMs proved useful for determining best models amongst hundreds of variables with linear or non-linear effects within minutes. SEMs, however, did not add as much information as in global-scale studies because of the exclusion of latitude and climatic predictors (as non-significant), which have strong direct effects and indirect effects via floristic and edaphic variables on soil microbiome over larger geographic scales (Tedersoo et al., 2014; Bahram et al., 2018). As a downside, our results can be directly extrapolated to a relatively narrow climate belt in North Europe, with more caution required when interpreting to Central European, North American or Asian conditions. Although comparable climatic regions in these continents harbor similar soil conditions and share many tree genera, many other genera differ and may have strong influence on soil biota similarly to *P. abies* and *P. sylvestris* in our study (Wu et al., 2018). However, we believe that many of the general biodiversity patterns recovered in our study apply to other temperate habitats with comparable gradients in soil properties.

The main technical issues emerging from this study are the temporal sampling effect, investigator bias and sample spoilage by molds. While these could be modeled and accounted for in our dataset, this is far more complicated for smaller datasets and especially global datasets, where samples from different regions are typically collected by distinct persons on a specific time point, generating a set of confounding geographic, temporal and investigator effects that cannot be disentangled. In our samples, both the temporal effect and investigator bias were mostly related to the relative abundance of molds, suggesting that molds may become problematic after certain weather events such as drying-rewetting or freeze-thaw (Blazewicz et al., 2014; Castano et al., 2018a; Burke et al., 2019) and depend on individual-specific care during sample pre-treatment. Therefore, we recommend that the abundance of molds, spatial and temporal sampling effects and investigator biases be at least considered in diversity models whenever such information is available.

## Conclusion

Our multi-year, regional-scale investigation demonstrates that taxonomic and functional distribution of much of the fungal kingdom follows soil pH more than previously expected. The pH effect also prevailed in comparisons of habitat types and anthropogenic impact. SEMs and correlation analyses indicated that soil pH is related to soil Ca concentration and the abundance of most tree species, which also affected both richness and composition of fungal groups. Accounting for pH, woody plant species richness has an independent positive effect on overall fungal diversity, which integrates differential biotic and abiotic tree species effects on different fungal guilds and taxonomic groups. Partly through altering soil pH, the conifers *Picea abies* and *Pinus sylvestris* serve as important tree species with strongest influence on diversity and composition of most fungal groups in North European forest ecosystems, which renders these tree species as keystone components in the soil microbiome perspective.

The analyses revealed mixed support to our hypotheses. In line with the first hypothesis, biotrophic groups responded more strongly to dominant vegetation than free-living groups, but both were generally more affected by soil pH. In contrast to second hypothesis, diversity of soil fungi tended to be higher in island habitats due to the edge effect. However, as predicted, fungal richness declined with island distance and in response to forest fragmentation. In agreement with the third hypothesis, pristine habitats supported somewhat higher fungal diversity, but there were no differences between natural and anthropogenic habitats focusing mostly on parks and coppiced gardens. In support to the fourth hypothesis, diversity of most fungal groups suffered from management of seminatural woodlands and parks (coppice cutting and mowing) and forests (selective harvesting or thinning of trees), but especially for forests the results depended on fungal group and time since partial harvesting.

With no effect of microclimatic variables, the North European soil mycobiome is not vulnerable to *direct* effects of climate change. However, increasing drought periods and pest or pathogen outbreaks may alter tree composition with further effects on soil microbes. Substantial differences in richness of fungi and several functional groups across habitat types and in response to management suggest that shifts in land use may greatly affect fungal diversity and functional guild composition.

## Data Availability Statement

The datasets presented in this study can be found in online repositories. The names of the repository/repositories and accession number(s) can be found in the article/Supplementary Material.

## Author Contributions

LT designed the study. LT, SA, RD, IH, RL, RR, ER, KA, TD, HT, KJ, IS, EO, SP, MM, KL, and AA collected the samples. SA, MB, KP, FB, AP, NH-D, VM, DG, RA, RP, IV, UK, and KA performed the analyses. LT wrote the manuscript with input from other authors. All authors contributed to the article and approved the submitted version.

## Conflict of Interest

The authors declare that the research was conducted in the absence of any commercial or financial relationships that could be construed as a potential conflict of interest.

## Footnotes

1 https://register.metsad.ee/#/

2 https://geoportaal.maaamet.ee/est/Ruumiandmed/Mullastiku-kaart-p33.html

3 https://geoportaal.maaamet.ee/est/Ruumiandmed/Eesti-topograafia-andmekogu-p79.html
