# Supplementary material for: Regional-Scale In-Depth Analysis of Soil Fungal Diversity Reveals Strong pH and Plant Species Effects in Northern Europe
Source: Front Microbiol. 2020 Sep 4;11:1953. doi: 10.3389/fmicb.2020.01953 (PMC7510051; doi:10.3389/fmicb.2020.01953)
Supplement: Supplementary file 2 [file Data_Sheet_1.PDF]

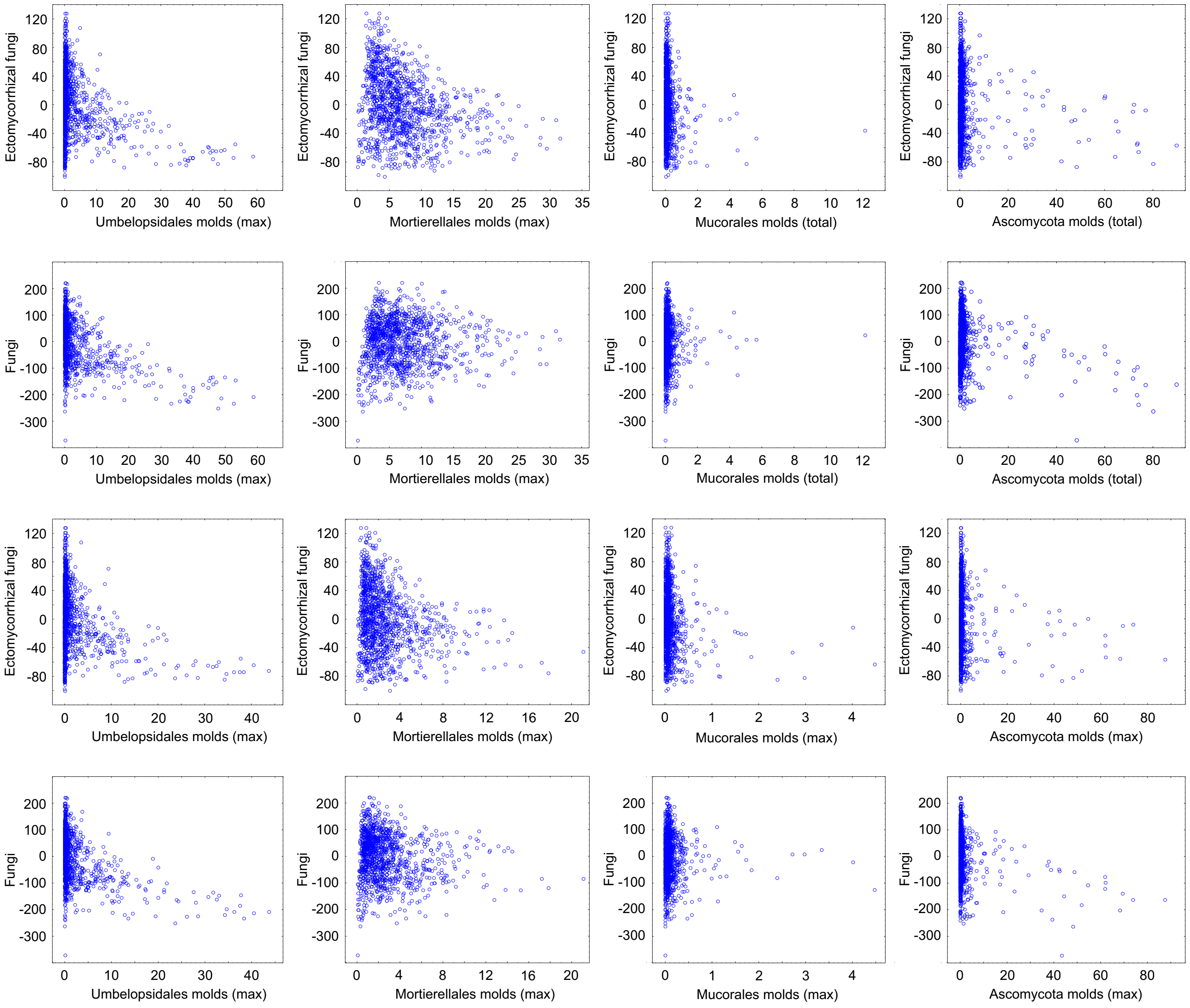

Figure S1. Correlation of total abundance of mold taxa and maximum abundance of mold OTUs per sample with residual richness of ectomycorrhizal fungi and all fungi. Relative abundances are back-transformed from log-ratio scale and expressed as percentages.

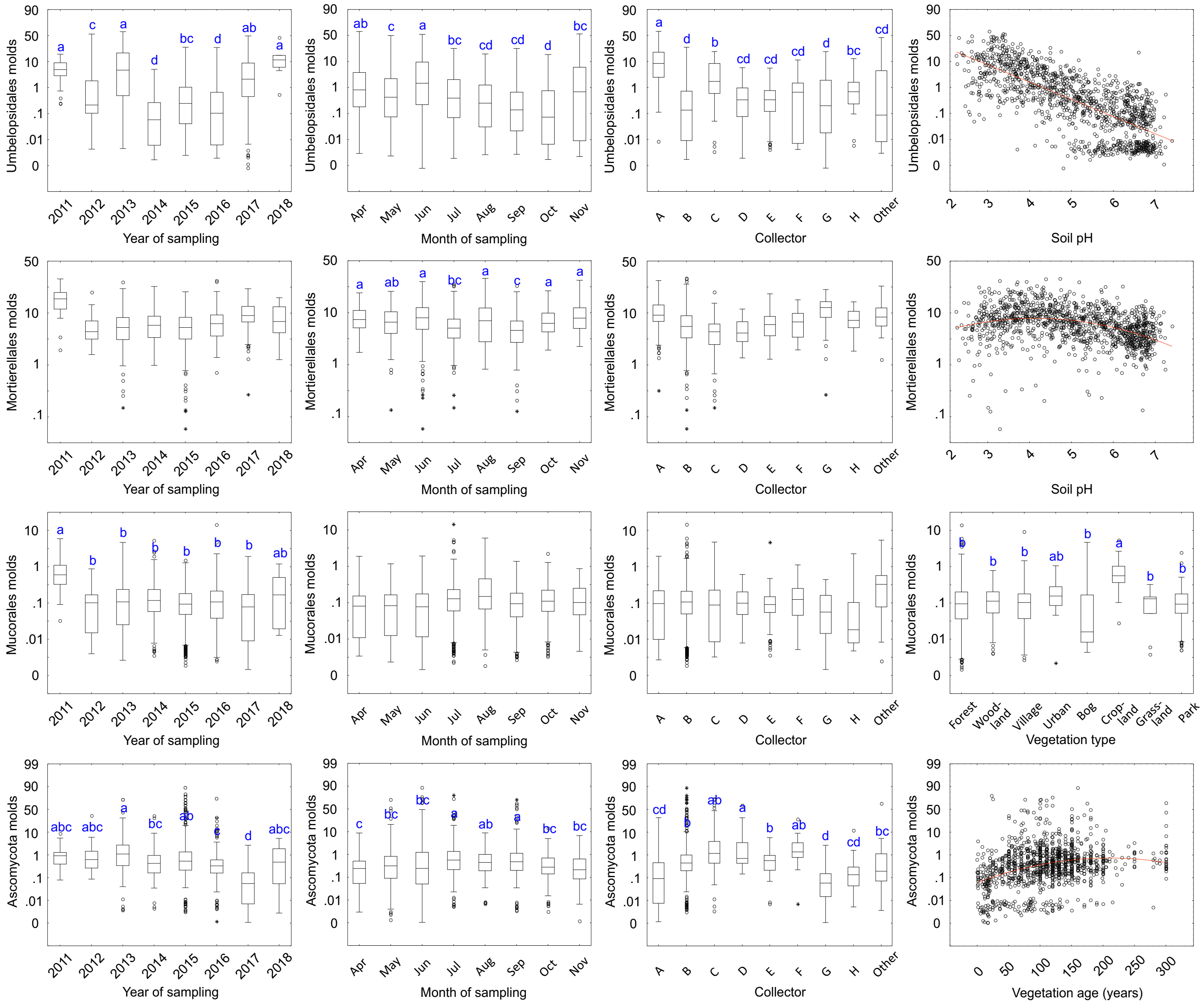

Figure S2. The effects of sampling year, sampling month and collector and other most important factors (right panes) on relative abundance of molds belonging to various taxonomic groups - Umbelopsidales, Mortierellales, Mucorales and Pezizomycotina (mostly Aspergillaceae). Relative abundances are expressed as percentages on a log-ratio scale. In box plots, the middle line, boxes, whiskers and circles represent medians, quartiles and 90% quantiles and outliers, respectively. In scatter plots, linear or quadratic regressions were fitted as in the best GLMs. Different letters above whiskers indicate statistically different groups.

Random forest and general linear modelling revealed that soil pH had the strongest effect on relative abundances of Umbelopsidales (R^2=0.215) and its OTU maxima (R^2=0.072). Mortierellales total abundance (R^2=0.113) and maximum abundance (R^2=0.068) were also related to soil pH. Mucorales abundance (R^2=0.024) and maximum OTU abundance (R^2=0.048) were greatest in croplands (Table S5; Figure 2). Pezizomycotina mold relative abundance was only weakly related to any environmental variables, but maximum OTU abundance was strongly negatively related to Mortierellales mold relative abundance (R^2=0.171). Overall, zygomycete molds were negatively correlated to Pezizomycotina molds, but abundances of zygomycete mold groups were all weakly positively correlated (0.101<R<0.366). Temporal variables were important for explaining the relative abundance of all groups of molds (Table S5; Figure S2). Year and month explained the relative abundance of Umbelopsidales (R^2=0.112 and R^2=0.012, respectively) and its OTU maximum abundance (R^2=0.135 and R^2=0.008, respectively). Four temporal variables (including month; R^2_{tot}=0.115) accounted for Mortierellales relative abundance, whereas the year of sampling (R^2=0.238) best explained the Mortierellales maximum OTU abundance. Mucorales relative abundance and maximum OTU abundance were best explained by a combination of three temporal variables (R^2_{tot}=0.072) and year (R^2=0.072), respectively. Pezizomycotina mold relative abundance and maximum OTU abundance were related to year (R^2=0.248, R^2=0.018, respectively) and month (R^2=0.027, R^2=0.020, respectively). The collector best explained the OTU maximum abundance of Mortierellales (R^2=0.272), Umbelopsidales (R^2=0.274) and Pezizomycotina (R^2=0.042) as well as relative abundances of these groups (R^2=0.021, R^2=0.302, R^2=0.020, respectively).

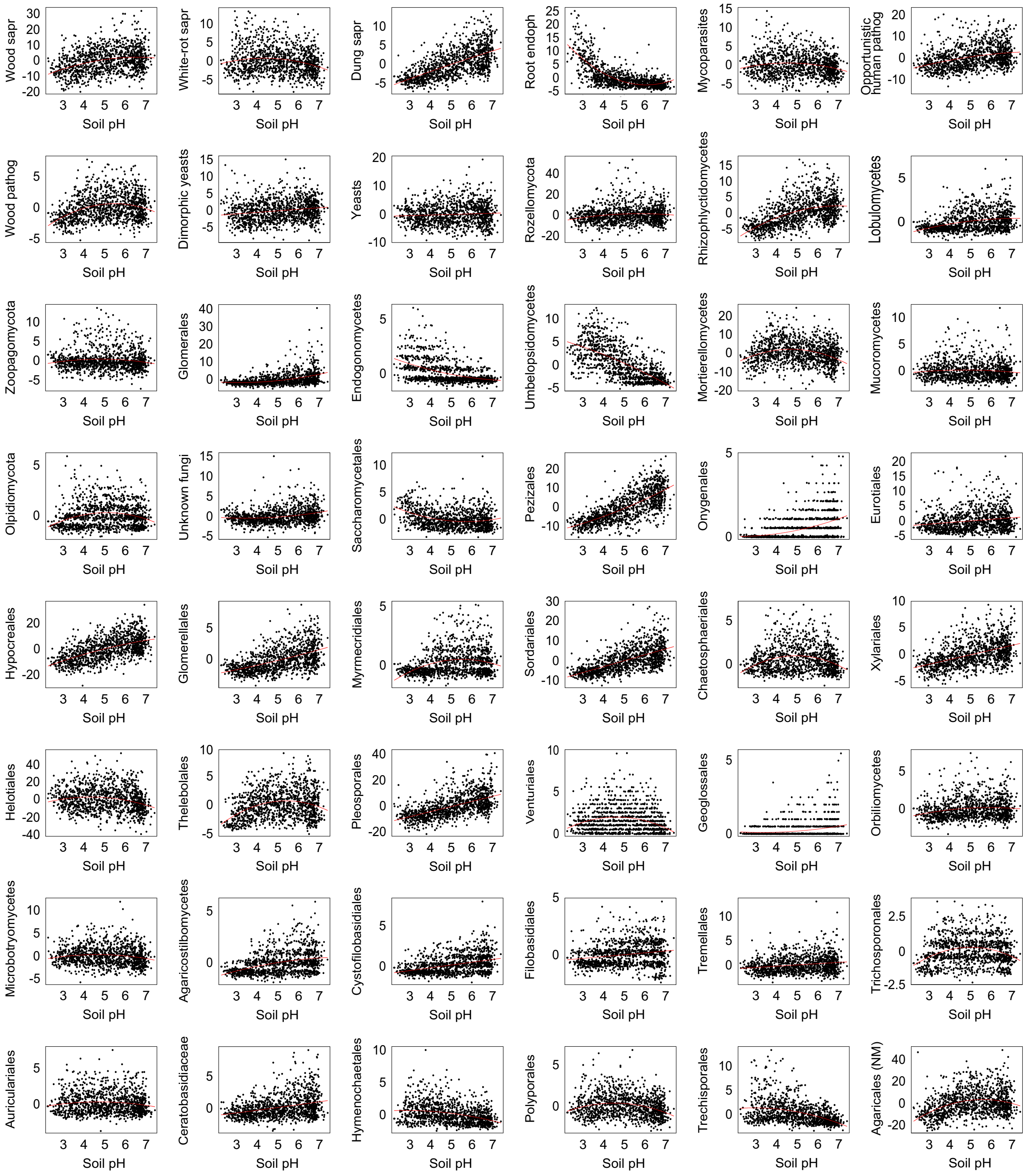

Figure S3. Effect of soil pH on residual richness of fungal functional and taxonomic groups. Red lines reflect best-fitting linear or quadratic regressions.

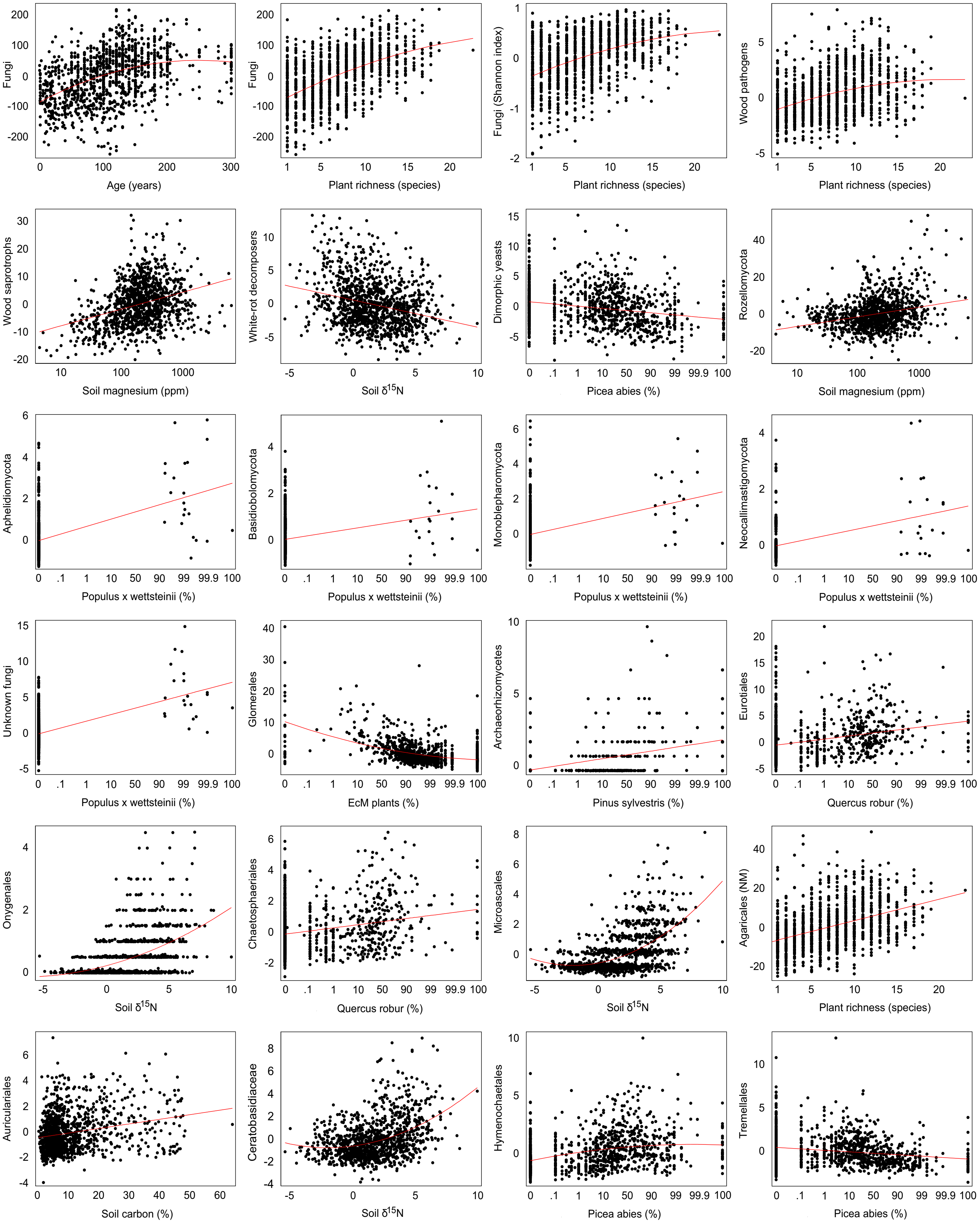

Figure S4. Effect of most important predictors other than pH on residual richness of fungal functional and taxonomic groups. Red lines reflect best-fitting linear or quadratic regressions.

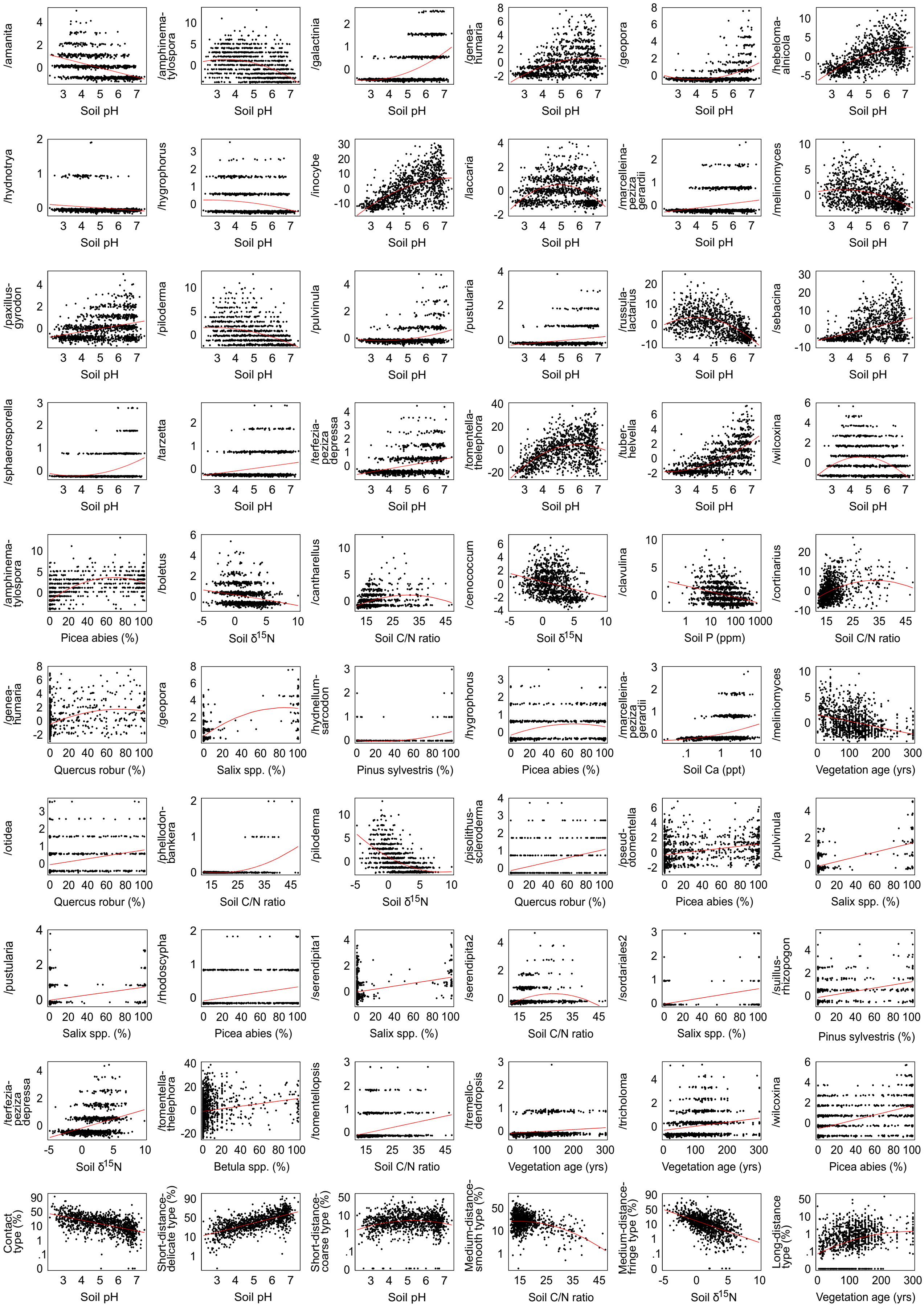

Figure S5. Response of OTU richness of ectomycorrhizal fungal lineages and relative abundance of exploration types (bottom pane) to soil pH and other environmental predictors (if most important according to general linear modelling).

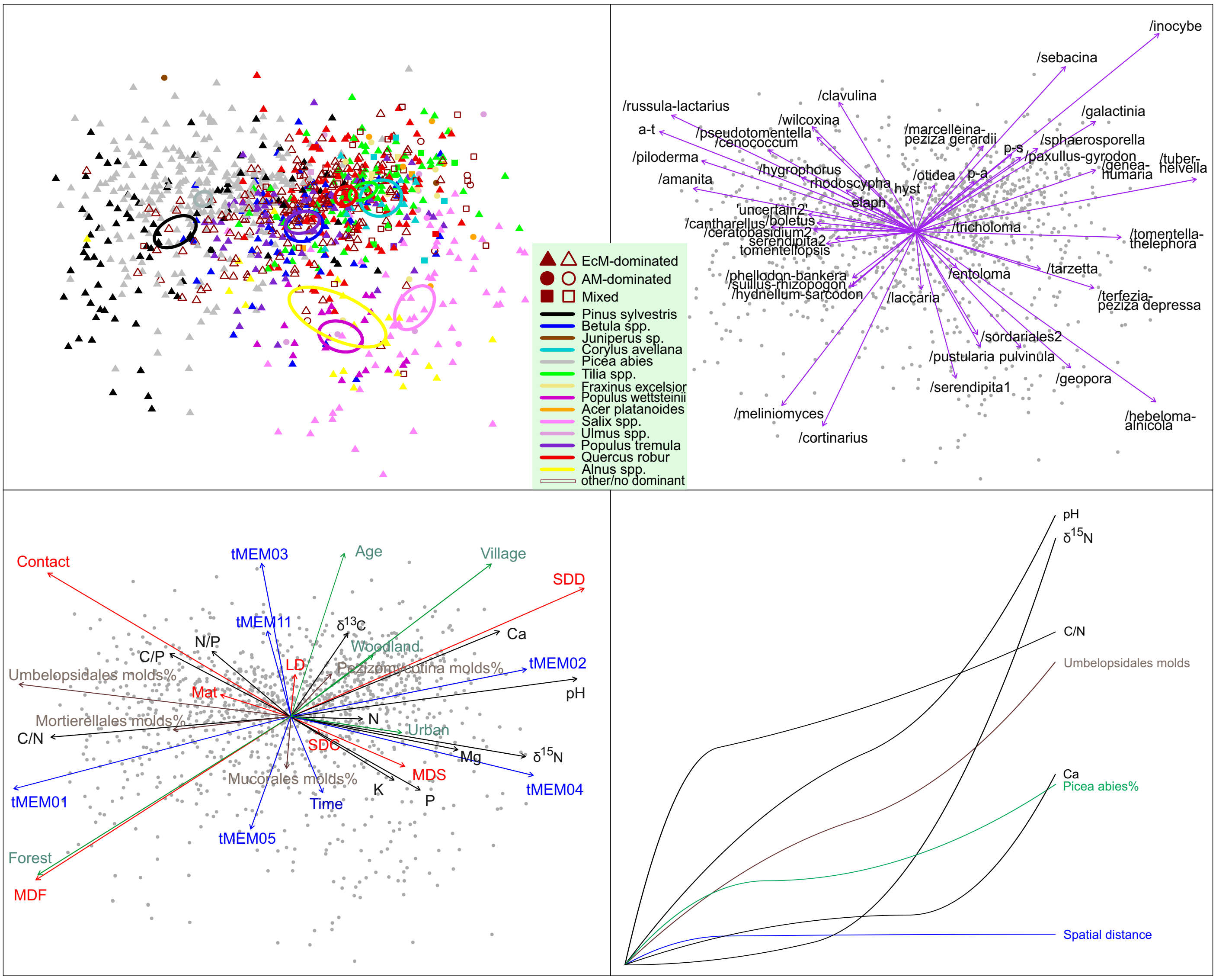

Figure S6. NMDS and GDM community profiles of ectomycorrhizal fungi across all sites based on richness of lineages. Circles, 95% ordi-ellipses for tree taxa; arrows, fit with environmental variables and placement of ectomycorrhizal fungal lineages and exploration types in top-right pane (abbreviations: a-t, /amphinema-tylospora; elaph, /elaphomyces; hyst, /hysterangium; p-a, /pachyphloeus-amylascus; p-s, /pisolithus-scleroderma) and bottom-left pane (abbreviations: LD, long-distance; MDF, medium-distance fringe; MDS, medium-distance smooth; SDC, short-distance coarse; SDD, short-distance delicate; colours depict different categories of variables), respectively. Bottom-right pane of indicates the cumulative effect of environmental variables in the relative scale of minimum (left) to maximum (right) values as revealed from GDM.

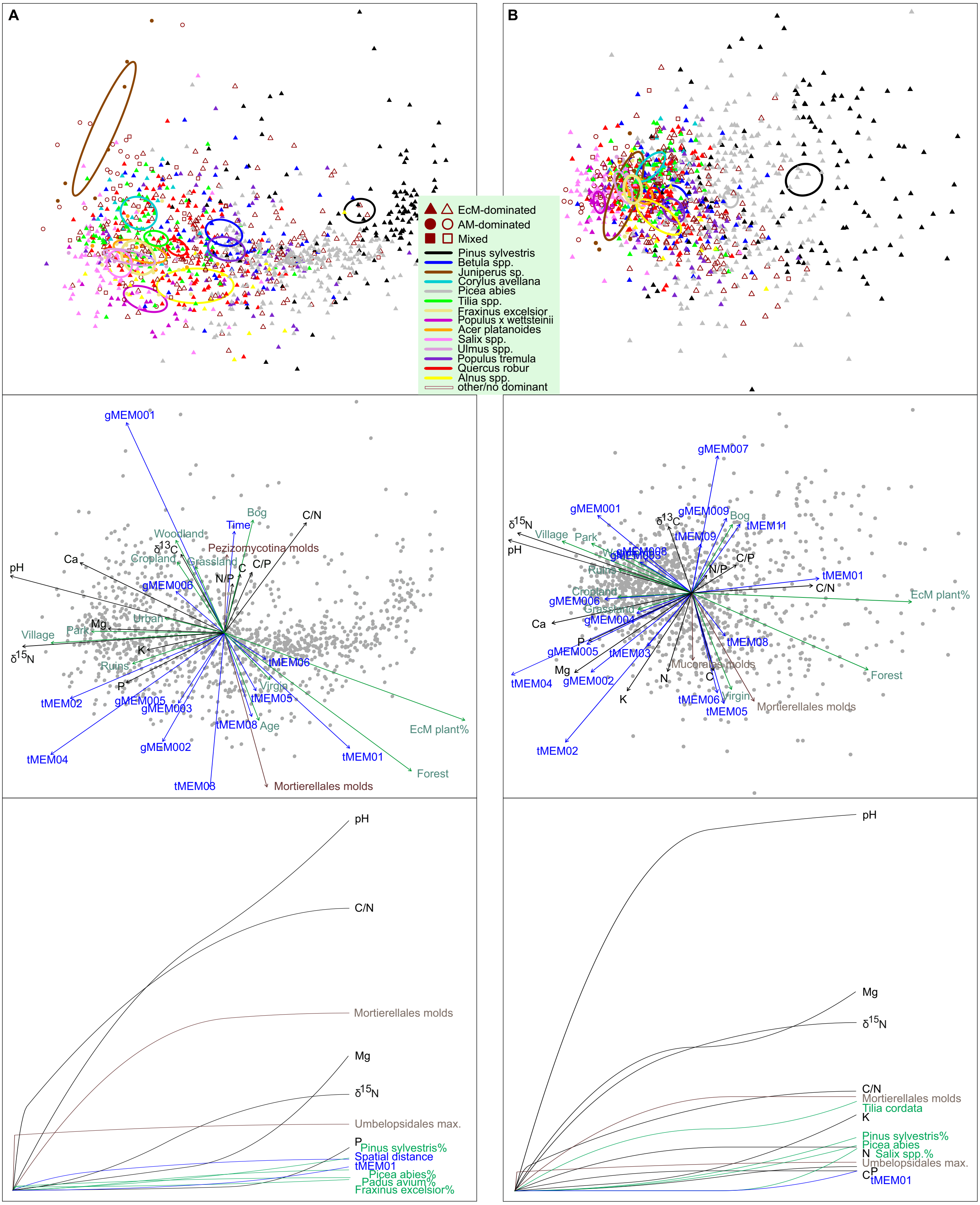

Figure S7. NMDS and GDM community profiles of A) saprotrophic fungi and B) plant pathogens. Circles, 95% ordiellipses for tree taxa; in central panes, arrows represent fit with environmental variables; in bottom panes, lines indicate the cumulative effect of environmental variables in the relative scale of minimum (left) to maximum (right) values as revealed from GDM.

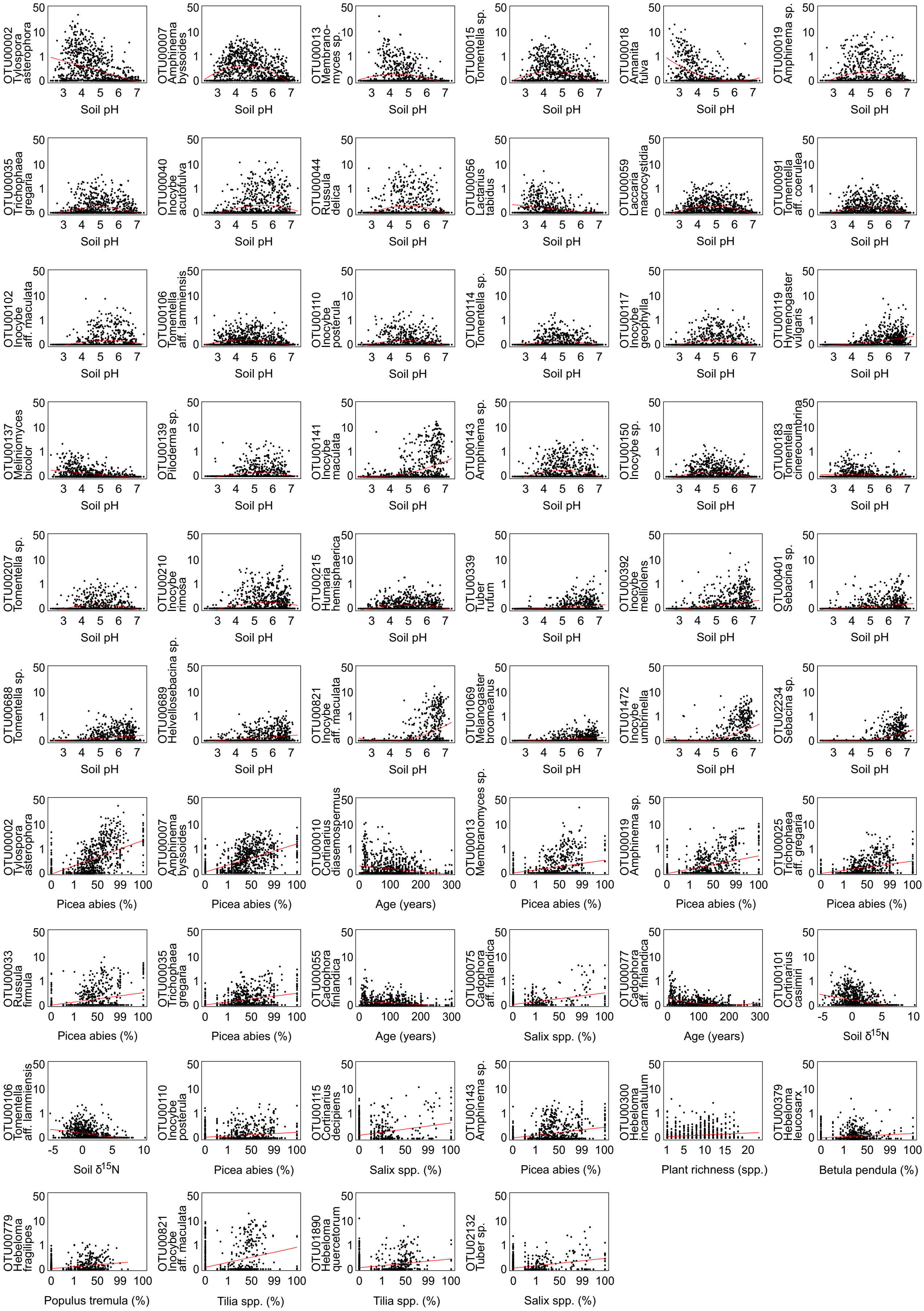

Figure S8. Best predictors of most frequent individual OTUs of EcM fungi. Note the log-ratio scale for OTU relative abundances.

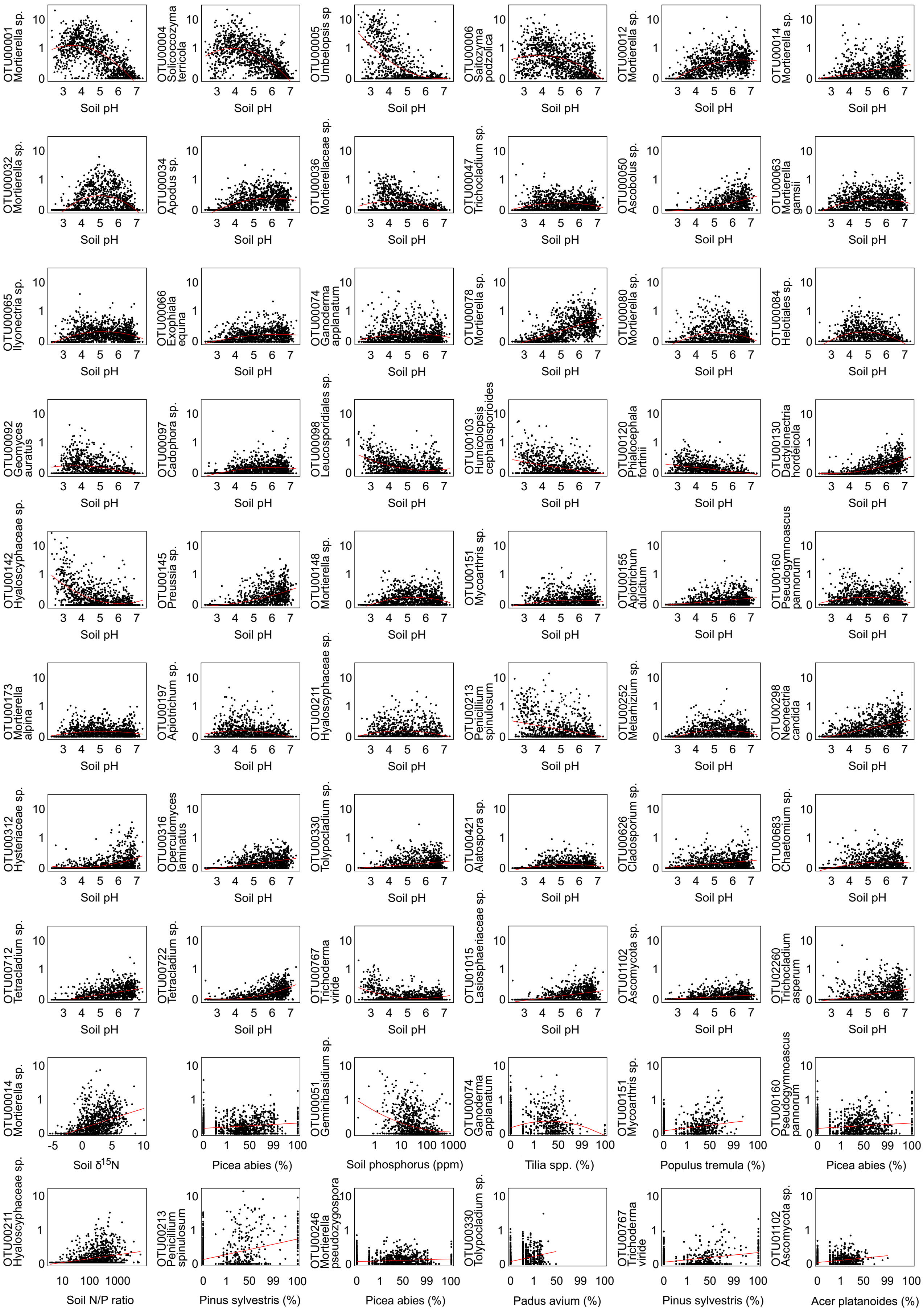

Figure S9. Best predictors of most frequent individual OTUs of non-EcM fungi. Note the log-ratio scale for OTU relative abundances.

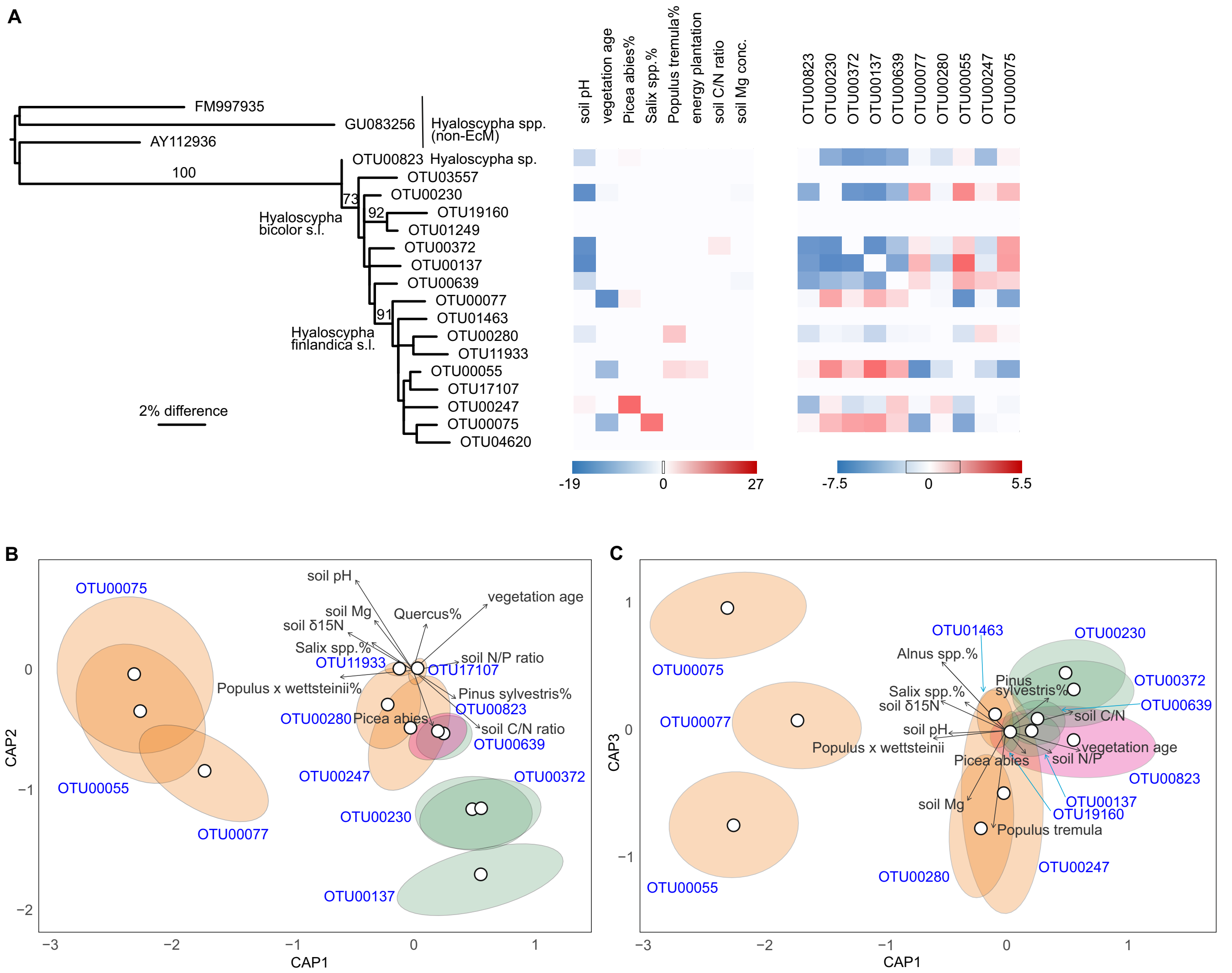

Figure S10. Niche analysis of OTUs belonging to the *Hyaloscypha finlandica-H. bicolor* species complex. A) Maximum likelihood phylogram (numbers above branches indicate bootstrap support) with heatmap-presentation of best predictors and co-occurrence analysis (box corresponds to non-significant effects at α=0.05). B-C) Placement of OTUs in Cap ordination space (open circles, OTU centroids; ellipses, their 1.96-fold standard deviation; purple, green and orange colours represent *Hyaloscypha* sp., *H. bicolor* and *H. finlandica*, respectively; arrows, environmental effects).

Methods: Representative sequences of all OTUs identified as *Hyaloscypha finlandica* (syn. *Cadophora finlandica*, *Meliniomyces finlandicus*) and *H. bicolor* (syn. *M. bicolor*) were aligned with the closest outgroup sequences obtained from UNITE using MAFFT 7 (https://mafft.cbrc.jp/alignment/server/), followed by manual editing and trimming. The alignment (all ingroup sequences 96-98% similar) was subjected to Maximum Likelihood phylogenetic analysis using RAxML 8.2.12 with 100 rapid bootstrap replicates as implemented in the CIPRES online workflow (https://www.phylo.org/; panel A). The initial steps of niche modelling included random forest and GLM as described in the main text. The explained variance of GLM model is illustrated as a heat map (panel B). To analyze OTU-environment relationship for the OTUs belonging to Hyaloscypha species, we performed a redundancy analysis (RDA; capscale function in vegan package) based on the centered log-ratio (CLR) transformed sequence counts corresponding to Aitchison distance (Gloor et al. 2017; Front. Microbiol. 8:2224). Prior to the ordination, predictors were Z-score-normalized. The uncertainty in OTUs position in the ordination space was estimated using 10,000 bootstrap iterations and visualized as 95% confidence ellipses, which represent the areas of variability between bootstrap replicates. We also performed C-score analysis to determine the number of checkerboard units between all pairs of Hyaloscypha OTUs, compared to a null model that maintains the number of presences (r00), as implemented in the vegan package.

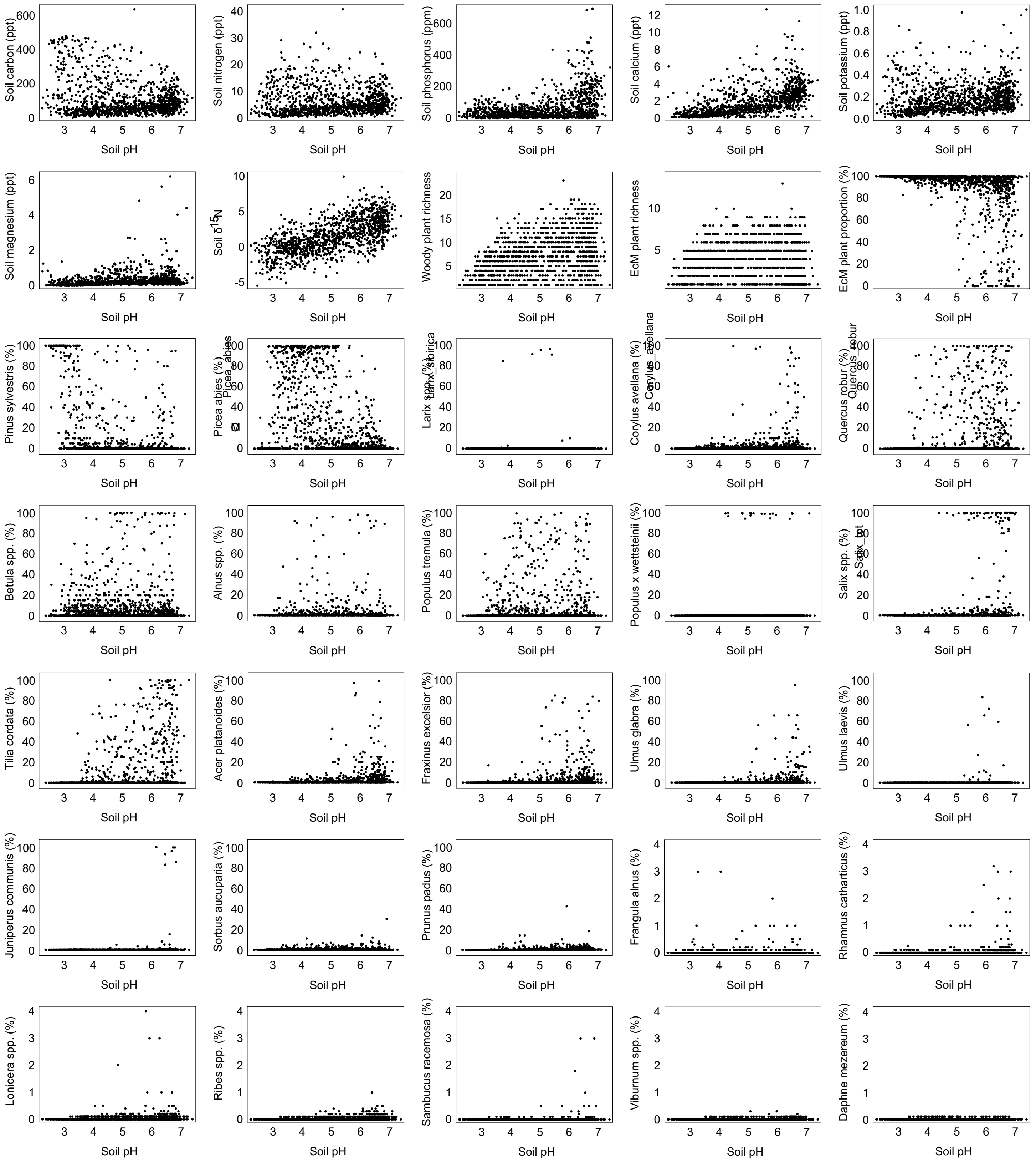

Figure S11. Correlation of soil pH with other soil properties and floristic variables.

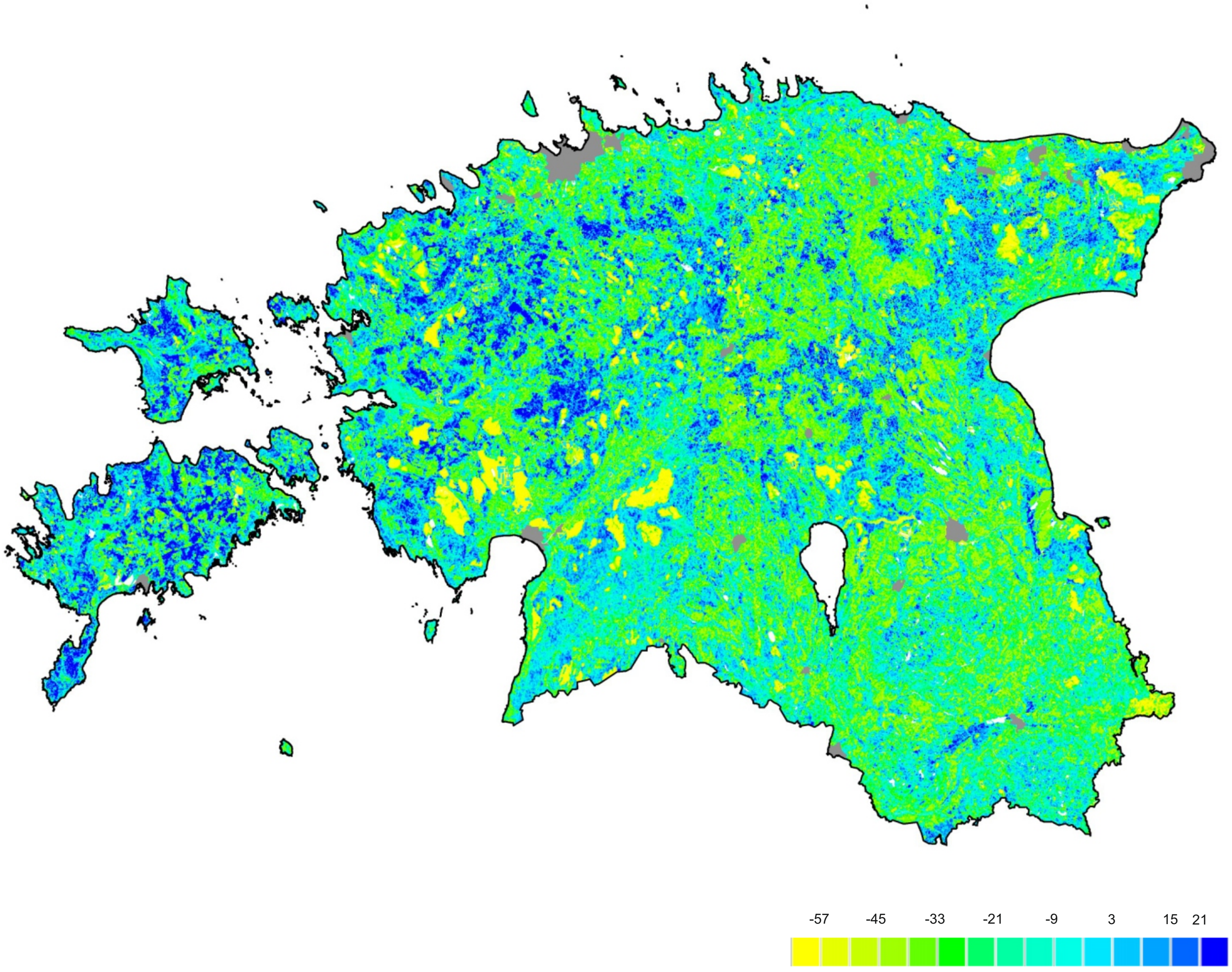

Figure S12. Extrapolated ectomycorrhizal fungal richness map of Estonia. Colors represent relative richness of fungi accounting for land use type and estimated soil litho-genetical scalar (acidity), estimated soil moisture scalar (humidity) and vegetation data as based on the best GLM model. Grey represents towns, roads and buildings.

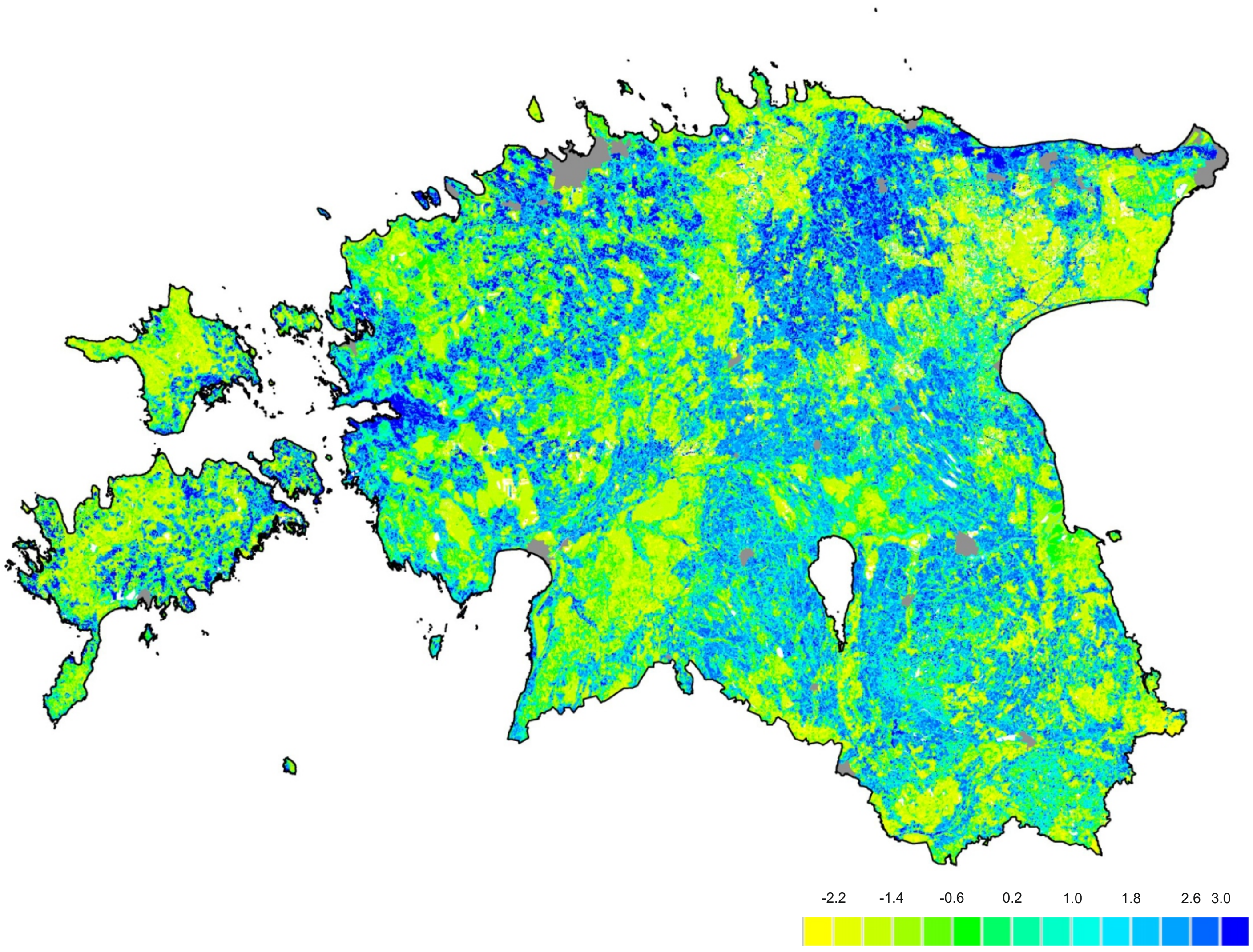

Figure S13. Extrapolated AM fungal richness map of Estonia. Colors represent relative richness of fungi accounting for land use type and estimated soil litho-genetical scalar (acidity), estimated soil moisture scalar (humidity) and vegetation data as based on the best GLM model. Grey represents towns, roads and buildings.

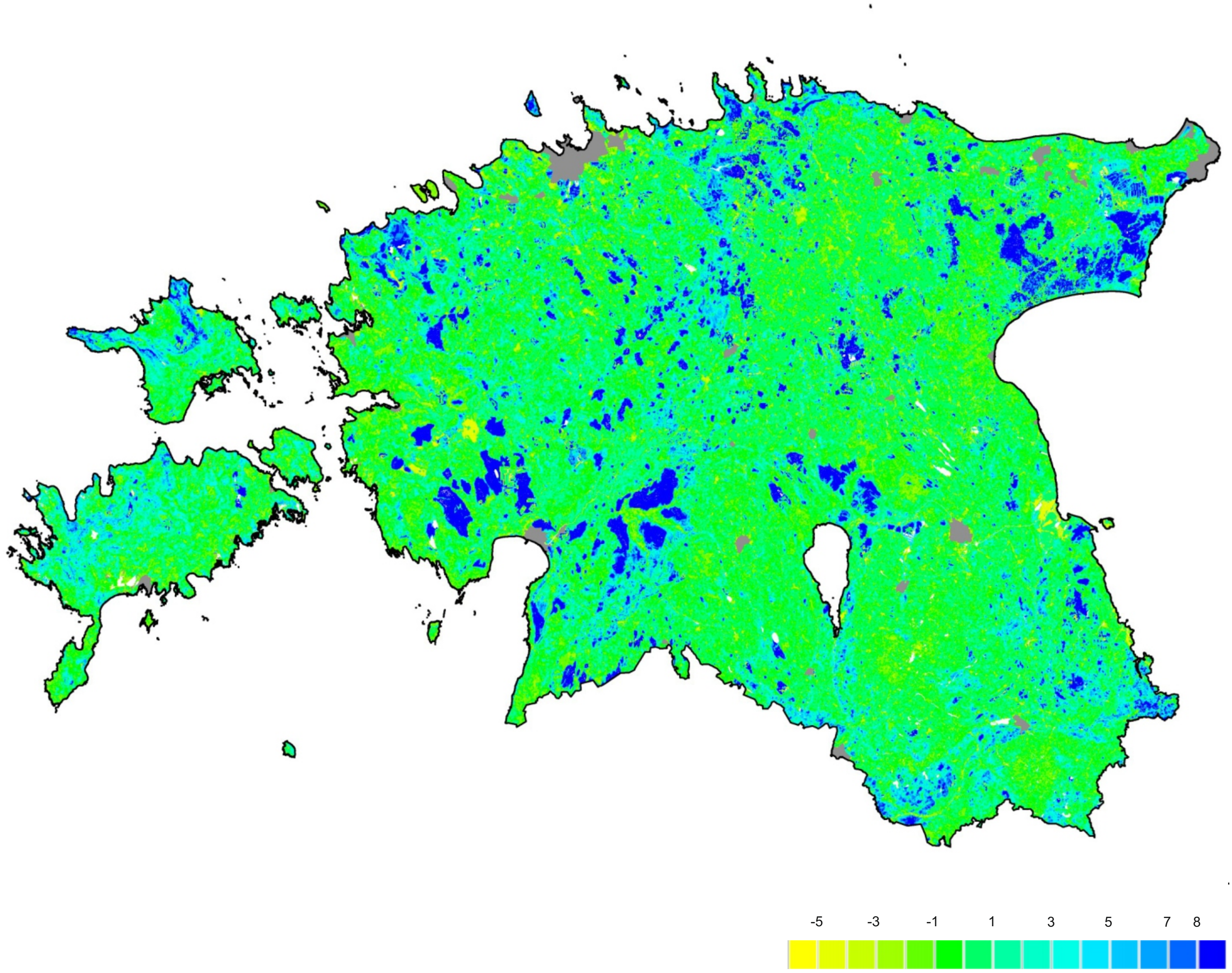

Figure S14. Extrapolated root endophyte richness map of Estonia. Colors represent relative richness of fungi accounting for land use type and estimated soil litho-genetical scalar (acidity), estimated soil moisture scalar (humidity) and vegetation data as based on the best GLM model. Grey represents towns, roads and buildings. Note that the high diversity of known root endophytes occurs in boggy areas, which may reflect biased knowledge of the root endophyte guild.

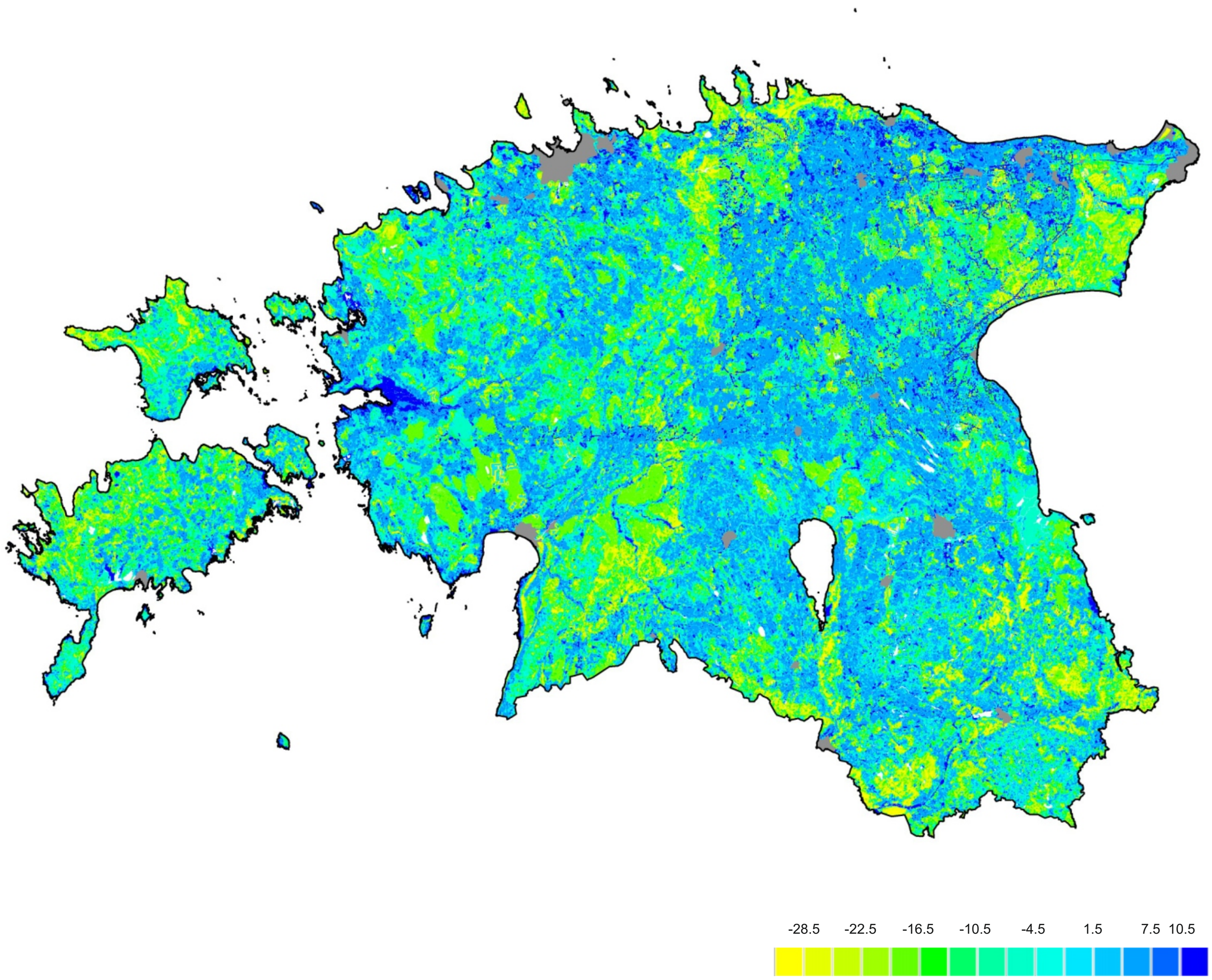

Figure S15. Extrapolated litter saprotroph richness map of Estonia. Colors represent relative richness of fungi accounting for land use type and estimated soil litho-genetical scalar (acidity), estimated soil moisture scalar (humidity) and vegetation data as based on the best GLM model. Grey represents towns, roads and buildings.

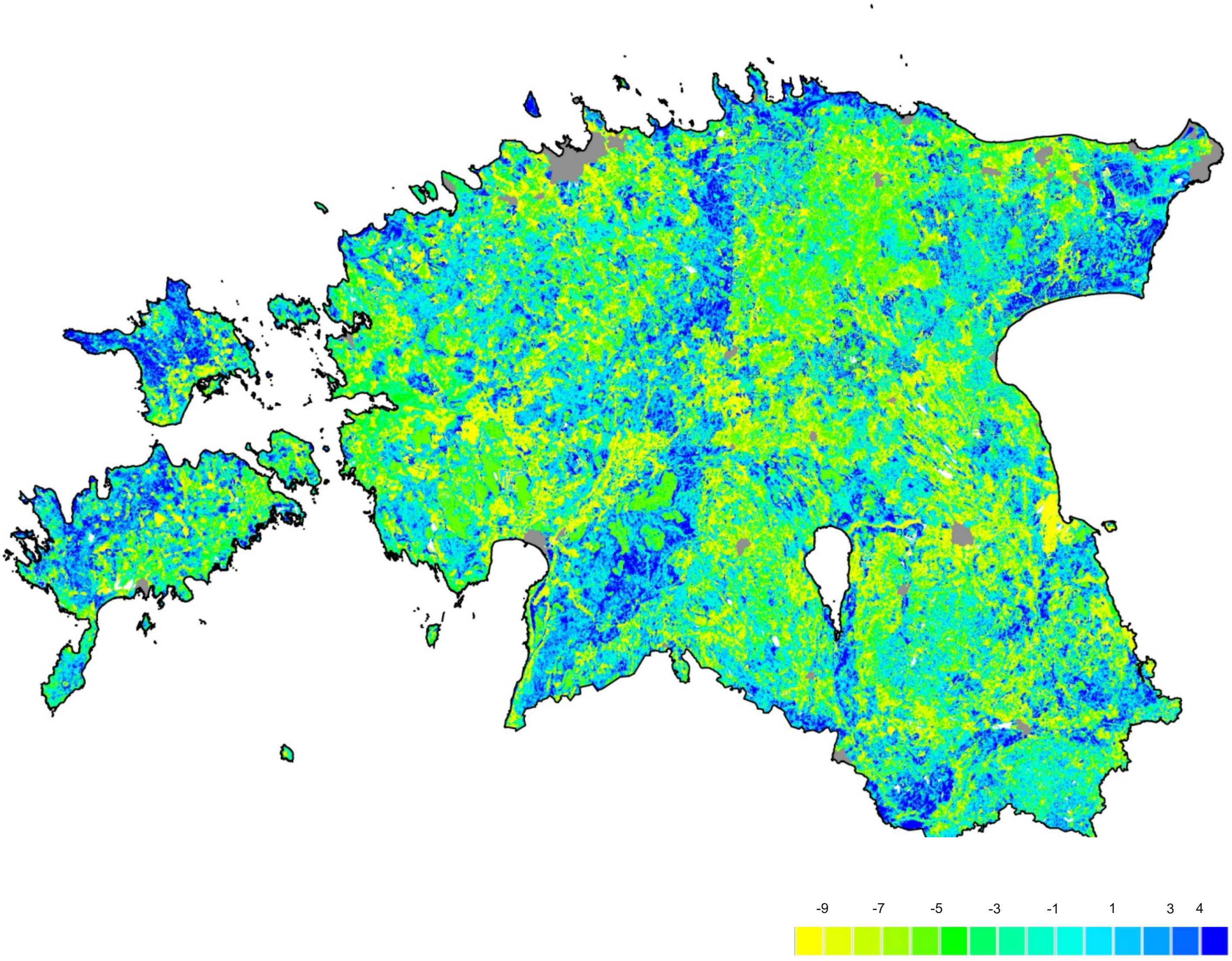

Figure S16. Extrapolated soil saprotroph richness map of Estonia. Colors represent relative richness of fungi accounting for land use type and estimated soil litho-genetical scalar (acidity), estimated soil moisture scalar (humidity) and vegetation data as based on the best GLM model. Grey represents towns, roads and buildings.

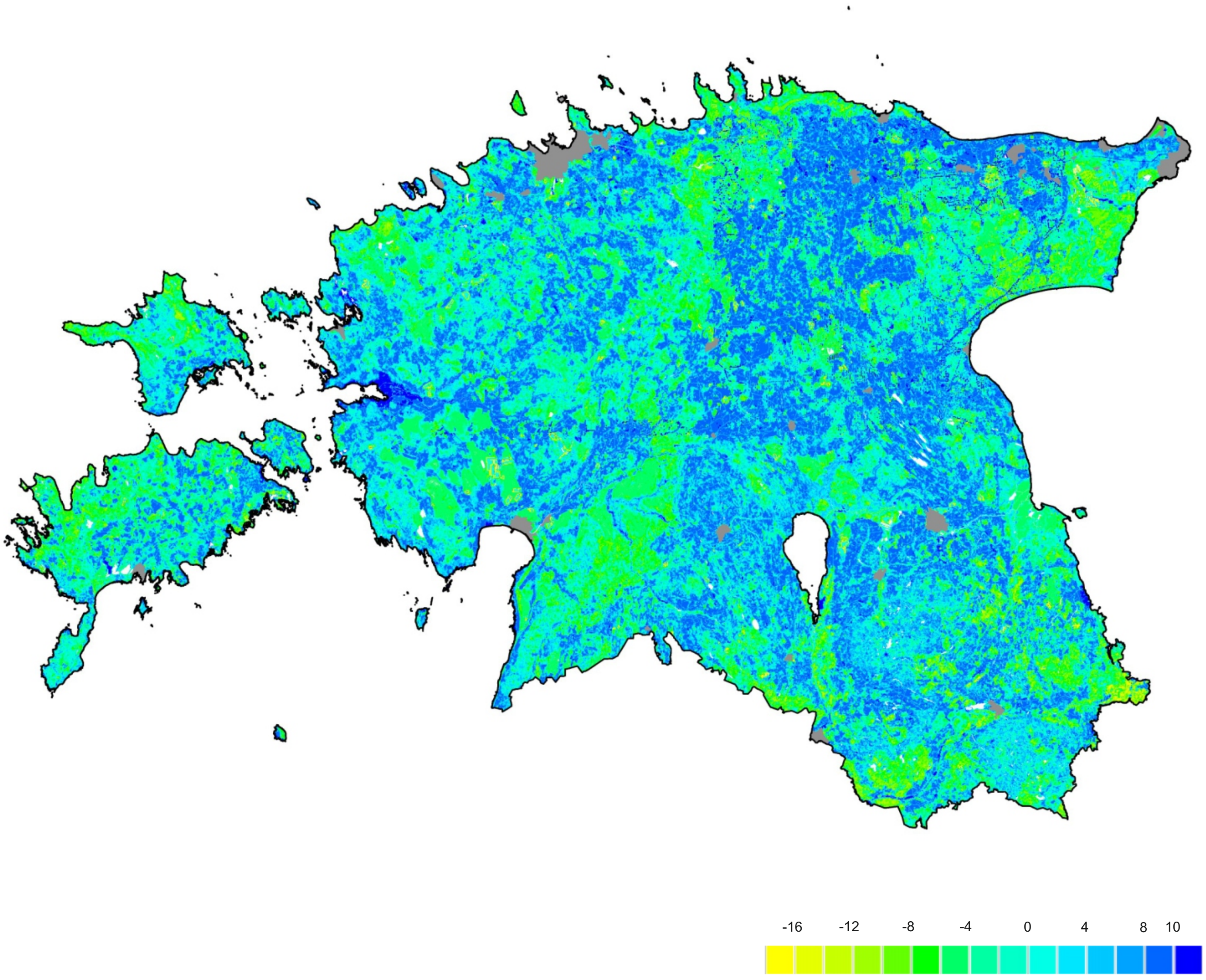

Figure S17. Extrapolated leaf pathogen richness map of Estonia. Colors represent relative richness of fungi accounting for land use type and estimated soil litho-genetical scalar (acidity), estimated soil moisture scalar (humidity) and vegetation data as based on the best GLM model. Grey represents towns, roads and buildings.

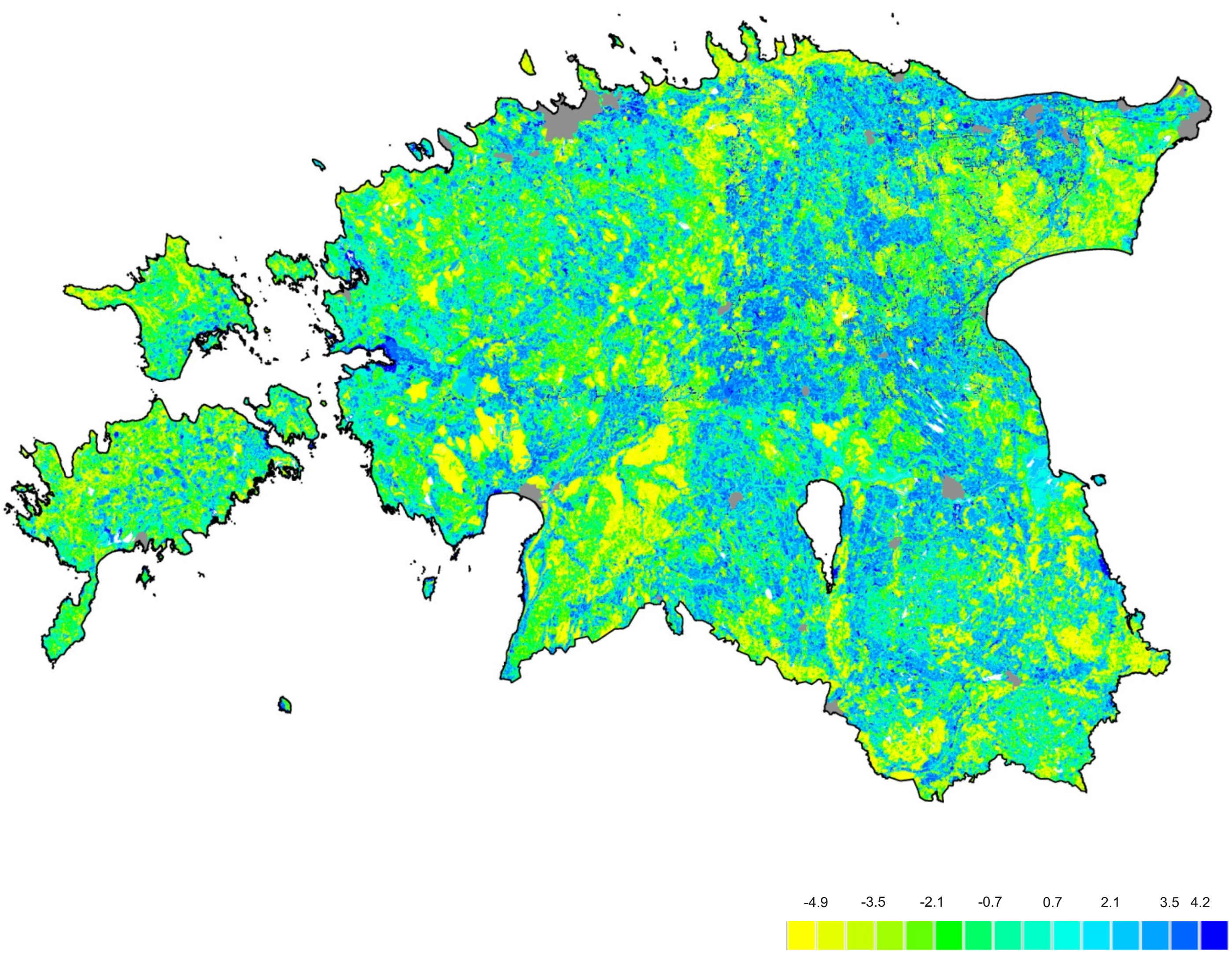

Figure S18. Extrapolated animal parasite richness map of Estonia. Colors represent relative richness of fungi accounting for land use type and estimated soil litho-genetical scalar (acidity), estimated soil moisture scalar (humidity) and vegetation data as based on the best GLM model. Grey represents towns, roads and buildings.
