# Supplementary material for: Regional-Scale In-Depth Analysis of Soil Fungal Diversity Reveals Strong pH and Plant Species Effects in Northern Europe
Source: Front Microbiol. 2020 Sep 4;11:1953. doi: 10.3389/fmicb.2020.01953 (PMC7510051; doi:10.3389/fmicb.2020.01953)
Supplement: Supplementary file 3 [file Data_Sheet_2.PDF]

Table S1. Sample metadata. **See separate .xls file**

Table S2. Characteristics of explanatory variables; ">" indicates calculated secondary variables.

Variable	Type	Transformation	Comments
sample	ID	excluded	sample ID
latitude	continuous	excluded	latitude (DD.DDDDD)
longitude	continuous	excluded	longitude (DD.DDDDD)
altitude	continuous	none	altitude (m above sea level)
>gMEM1-319	continuous	none	eigenvectors of geographical space
collector	categorical (8 levels + other for persons with <10 samples)	dummy/none	code for person collecting and pre-processing samples
sampling date	continuous	excluded	date of sample collection
>year of sampling	categorical (8 levels)	dummy/none	year (unordered)
>month of sampling	categorical (8 levels; March is incorporated into April; December to February are incorporated into November)	dummy/none	year (unordered)
>linear time	continuous	none/sqrt	time since sampling (days)
>tMEM1-74	continuous	none	eigenvectors of linear time
woody plant richness	continuous	none/sqrt	richness of all woody plants
EcM plants sampled	continuous	none/sqrt	richness of EcM plants
EcM plant%	continuous	log-ratio/(none)	% of EcM plants on basal area or coverage (shrublands, meadows) basis
Populus tremula%	continuous	log-ratio/(none)	% basal area of *Populus tremula*
Picea abies%	continuous	log-ratio/(none)	% basal area of *Picea abies*
Betula spp.%	continuous	log-ratio/(none)	% basal area of *Betula* spp.
Tilia spp.%	continuous	log-ratio/(none)	% basal area of *Tilia* spp.
Quercus robur%	continuous	log-ratio/(none)	% basal area of *Quercus robur*
Corylus avellana%	continuous	log-ratio/(none)	% basal area of *Corylus avellana*
Pinus sylvestris%	continuous	log-ratio/(none)	% basal area of *Pinus sylvestris*
Alnus incana%	continuous	excluded	% basal area of *Alnus incana*
Alnus glutinosa%	continuous	excluded	% basal area of *Alnus glutinosa*
>*Alnus* spp.%	continuous	log-ratio/(none)	% basal area of >*Alnus* spp.
Salix alba%	continuous	excluded/(log-ratio)	% basal area of *Salix alba*
Salix caprea%	continuous	excluded/(log-ratio)	% basal area of *Salix caprea*
Salix cinerea%	continuous	excluded/(log-ratio)	% basal area of *Salix cinerea*
Salix fragilis%	continuous	excluded/(log-ratio)	% basal area of *Salix fragilis*
Salix other%	continuous	excluded/(log-ratio)	% basal area of *Salix* other
Salix pentandra%	continuous	excluded/(log-ratio)	% basal area of *Salix pentandra*
Salix triandra%	continuous	excluded/(log-ratio)	% basal area of *Salix triandra*
Salix viminalis%	continuous	excluded/(log-ratio)	% basal area of *Salix viminalis*
>*Salix* spp.%	continuous	log-ratio/(none)	% basal area of >*Salix* spp.
Abies alba%	continuous	log-ratio/(none)	% basal area of *Abies alba*
Fagus sylvatica%	continuous	log-ratio/(none)	% basal area of *Fagus sylvatica*
Helianthemum nummularium%	continuous	log-ratio/(none)	% coverage of *Helianthemum nummularium*
Arctostaphylos uva-ursi%	continuous	log-ratio/(none)	% coverage of *Arctostaphylos uva-ursi*
Larix sibirica%	continuous	log-ratio/(none)	% basal area of *Larix sibirica*

Pinus strobus%	continuous	log-ratio/(none)	% basal area of *Pinus strobus*
Populus alba%	continuous	log-ratio/(none)	% basal area of *Populus alba*
Populus berolinensis%	continuous	log-ratio/(none)	% basal area of *Populus berolinensis*
Populus x wettsteinii%	continuous	log-ratio/(none)	% basal area of *Populus wettsteinii*
Pseudotsuga%	continuous	log-ratio/(none)	% basal area of *Pseudotsuga menziesii*
Pyroleae%	continuous	log-ratio/(none)	% coverage of Pyroleae
Acer negundo%	continuous	log-ratio/(none)	% basal area of *Acer negundo*
Acer platanoides%	continuous	log-ratio/(none)	% basal area of *Acer platanoides*
Aesculus%	continuous	log-ratio/(none)	% basal area of *Aesculus hippocastaneum*
Amelanchier spicata%	continuous	log-ratio/(none)	% basal area of *Amelanchier spicata*
Aronia melanocarpa%	continuous	log-ratio/(none)	% basal area of *Aronia melanocarpa*
Berberis spp.%	continuous	log-ratio/(none)	% basal area of *Berberis* spp.
Caragana spp.%	continuous	log-ratio/(none)	% basal area of *Caragana* spp.
Cornus spp.%	continuous	log-ratio/(none)	% basal area of *Cornus* spp.
Cotoneaster spp.%	continuous	log-ratio/(none)	% basal area of *Cotoneaster* spp.
Crataegus spp.%	continuous	log-ratio/(none)	% basal area of *Crataegus* spp.
Daphne mezereum%	continuous	log-ratio/(none)	% basal area of *Daphne mezereum*
Euonymus%	continuous	log-ratio/(none)	% basal area of *Euonymus europaeus*
Frangula alnus%	continuous	log-ratio/(none)	% basal area of *Frangula alnus*
Fraxinus excelsior%	continuous	log-ratio/(none)	% basal area of *Fraxinus excelsior*
Hippophae%	continuous	log-ratio/(none)	% basal area of *Hippophae rhamnoides*
Lonicera xylosteum%	continuous	log-ratio/(none)	% basal area of *Lonicera xylosteum*
Malus spp.%	continuous	log-ratio/(none)	% basal area of *Malus* spp.
Myrica gale%	continuous	log-ratio/(none)	% basal area of *Myrica gale*
Prunus padus%	continuous	log-ratio/(none)	% basal area of *Prunus padus*
Philadelphus coronarius%	continuous	log-ratio/(none)	% basal area of *Philadelphus coronarius*
Prunus cerasus%	continuous	log-ratio/(none)	% basal area of *Prunus cerasus*
Prunus domestica%	continuous	log-ratio/(none)	% basal area of *Prunus domestica*
>Prunus spp%	continuous	log-ratio/(none)	% basal area of *Prunus* spp.
Pyrus communis%	continuous	log-ratio/(none)	% basal area of *Pyrus communis*
Rhamnus%	continuous	log-ratio/(none)	% basal area of *Rhamnus catharticus*
Ribes spp.%	continuous	log-ratio/(none)	% basal area of *Ribes* spp.
Rosa spp.%	continuous	log-ratio/(none)	% basal area of *Rosa* spp.
Sambucus racemosa%	continuous	log-ratio/(none)	% basal area of *Sambucus racemosa*
Sorbaria sorbifolia%	continuous	log-ratio/(none)	% basal area of *Sorbaria sorbifolia*
Sorbus aucuparia%	continuous	log-ratio/(none)	% basal area of *Sorbus aucuparia*
Sorbus intermedia%	continuous	log-ratio/(none)	% basal area of *Sorbus intermedia*
Spiraea spp.%	continuous	log-ratio/(none)	% basal area of *Spiraea* spp.
Symphoricarpos spp.%	continuous	log-ratio/(none)	% basal area of *Symphoricarpos* spp.
Syringa spp.%	continuous	log-ratio/(none)	% basal area of *Syringa* spp.
Ulmus glabra%	continuous	excluded	% basal area of *Ulmus glabra*
Ulmus laevis%	continuous	excluded	% basal area of *Ulmus laevis*
>*Ulmus* spp%	continuous	log-ratio/(none)	% basal area of *Ulmus* spp.
Viburnum spp.%	continuous	log-ratio/(none)	% basal area of *Viburnum* spp.
Juniperus communis%	continuous	log-ratio/(none)	% basal area of *Juniperus communis*
Thuja spp.%	continuous	log-ratio/(none)	% basal area of *Thuja* spp.
plant phylogeny	distance matrix	excluded	% basal area of plant phylogeny
>cdMEM1-44	continuous	none	eigenvectors of plant phylogenetic space based on comdist model
>ntMEM1-32	continuous	none	eigenvectors of plant phylogenetic space based on nearest taxon index model

>psMEM1-119	continuous	none	eigenvectors of plant phylogenetic space based on phylosor model
vegetation age	continuous	sqrt/log2/none	estimated/determined age of oldest plants
time since fire (years)	continuous	log10	estimated/determined time since last fire (years)
site size (m^2)	continuous	log2	site size (m^2)
vegetation type (for habitat type analysis)	categorical(forest, woodland, energy plantation, park, alley, grassland, cropland, ruins, wasteland, bog	dummy/none	the main category of vegetation type
1. forest	.	.	height of woody plants>6 m, coverage >50%, excluding other vegetation types below
2. woodland	.	.	wooded meadows and pastures that have been historically managed as such (historical tree coverage <50%); typically high tree and herb diversity, herb layer well-developed
3. energy plantation	.	.	short-rotation (>20 years) monospecific plantations of Populus wettsteinii and Salix viminalis for maximum wood production, highest-productivity ecosystems; typically multiple woody plants have self-established
4. park	.	.	tree-dominated parks (coverage >50%), where trees have been planted; exotic plant species common; herb layer typically moderately developed
5. alley	.	.	parks, where trees have been planted in 1-4 rows; typically poor in tree species, with well-developed herb layer
6. grassland	.	.	various types of herb-dominated habitats or shrublands (woody plants <2 m height), solitary trees may occur (<15% coverage)
7. cropland	.	.	fields of grassy, bushy or tree crops; typically monospecific plantations on fertilized soils
8. ruins	.	.	surroundings of abandoned houses; typically rich in fruit-trees, decorative trees and/or very old trees including exotic plants; soil is heterogeneous by including parts of construction wood and stones
9. wasteland	.	.	tree-dominated, coppiced habitats included sites for dumping garbage, old quarries, soviet military remains; exotic species are common
10. bog	.	.	non-forested (trees and bushes <3 m tall) habitats on peatlands
urbanisation category	categorical(wild forested, village, urban)	dummy/none	the main category of urbanisation for tree-dominated habitats
1. wild forested	.	.	ecosystems outside villages and cities
2. village	.	.	ecosystems in villages (pop <2000)
3. urban	.	.	ecosystems in towns and cities (pop >2000)

forest park	binary	dummy	managed old forests with multiple tracks near settlements for leisure or education purpose
wooded pasture	binary	dummy	woodlands that have been traditionally grazed by sheep or cattle or horses
wooded meadow	binary	dummy	woodlands that have been traditionally mown for fodder/hay
field island	binary	dummy	unnatural tree-dominated island surrounded by fields; typically dumping grounds for rocks, refuges for burrowing animals, colonised by pioneer tree species
bog island	binary	dummy	natural tree-dominated island surrounded by bog; typically hard-to-reach, old, protected habitats on acidic soils
aquatic island	binary	dummy	natural tree-dominated island surrounded by sea, lake or river; typically species-rich and old ecosystems on near-neutral soils
virgin forest	binary	dummy	forests of >120 years with no virtual signs of historical management and abundant dead wood in all decay classes
management (parks, woodlands only)	categorical (managed, unmanaged, coppiced)	dummy/none	management of parks and woodlands
1. managed	.	.	grazed or mown regularly or once per year
2. unmanaged	.	.	grazing or mowing has ceased several years ago, with abundant establishment of saplings and seedlings of trees
3. coppiced	.	.	grazing or mowing has ceased >1 year ago, but not yer coppiced
island/fragment size (ha)	continuous	log2	size of islands or isolated forest fragments (<1000 ha)
isolation distance (m)	continuous	log2	distance of isolated islands or forest fragments to closest forested mainland habitat
permanent water bodies	binary	dummy	presence of streams or permanent water holes in the site
type of harvesting	categorical (thinning, selective, sanitary)	used as binary for harvesting effect	type of selective harvesting
time since harvesting (years)	continuous	sqrt/none	determined/estimated time since the last harvesting event
proportion harvested	continuous	excluded	proportion of trees selectively harvested
soil $\delta^{15}N$	continuous	none/square	ratio of $^{15}N/^{14}N$ relative to standard
soil $\delta^{13}C$	continuous	none	ratio of $^{13}C/^{12}C$ relative to standard
soil carbon (ppt)	continuous	log10/log10+square	soil total C concentration (g/kg)
soil nitrogen (ppt)	continuous	log10/log10+square	soil total N concentration (g/kg)
soil phosphorus (ppm)	continuous	log10/log10+square	soil total P concentration (mg/kg)
soil potassium (ppm)	continuous	log10/log10+square	soil total K concentration (mg/kg)
soil calcium (ppm)	continuous	log10/log10+square	soil total Ca concentration (mg/kg)
soil magnesium (ppm)	continuous	log10/log10+square	soil total Mg concentration (mg/kg)
soil pH	continuous	none/square	soil pH (measured in 1M KCl solution)
MAT	continuous	none	mean annual temperature (*C) as based on microclimatic extrapolations
Mean_diurnal_range	continuous	none	mean diurnal temperature range as based on microclimatic extrapolations
Isothermality	continuous	none	mean isothermality as based on microclimatic extrapolations

Temp_max	continuous	none	mean maximum monthly temperature as based on microclimatic extrapolations
Temp_min	continuous	none	mean minimum monthly temperature as based on microclimatic extrapolations
Temp_range	continuous	none	mean maximum-minimum montly temperature as based on microclimatic extrapolations
Temp_wetQ	continuous	none	mean temperature of the driest quarter as based on microclimatic extrapolations
Temp_dryQ	continuous	none	mean temperature of the warmest quarter as based on microclimatic extrapolations
Temp_warmQ	continuous	none	mean temperature of the coldest quarter as based on microclimatic extrapolations
Temp_coldQ	continuous	none	mean temperature of the wettest quarter as based on microclimatic extrapolations
MAP	continuous	none	mean annual precipitation (mm) as based on microclimatic extrapolations
Prec_max	continuous	none	maximum mean monthly precipitation as based on microclimatic extrapolations
Prec_min	continuous	none	minimum mean monthly precipitation as based on microclimatic extrapolations
Precipitation Seasonality	continuous	none	coefficient of variation of mean quarterly precipitation as based on microclimatic extrapolations
Prec_wetQ	continuous	none	mean precipitation of the driest quarter as based on microclimatic extrapolations
Prec_dryQ	continuous	none	mean precipitation of the warmest quarter as based on microclimatic extrapolations
Prec_warmQ	continuous	none	mean precipitation of the coldest quarter as based on microclimatic extrapolations
Prec_coldQ	continuous	none	mean precipitation of the wettest quarter as based on microclimatic extrapolations
Prec_CV	continuous	none	coefficient of variation of mean monthly precipitation as based on microclimatic extrapolations
Temp_Yvar	continuous	none	coefficient of variation of mean monthly temperature as based on microclimatic extrapolations

Table S3. Bioinformatics workflow.

Process and program	Reference
PacBio CCS reads (minPasses = 3, MinAccuracy = 0.9) were generated using SMRT Link v 6.0.0.47841.	https://www.pacb.com/support/software-downloads/
Subsequent processes were performed in PipeCraft 1.0	Anslan et al. 2017
Quality filtering was performed using vsearch with maximum expected error threshold of 1 (--fastq_maxee = 1) and discarding sequences with ambiguous bases (--fastq_maxns = 0).	Rognes et al. 2016
Demultiplexing of the sequencing data was performed using mothur by allowing one mismatch to tags (bdiffs = 1) and two mismatches to primers (pdiffs = 2) as implemented in PipeCraft demultiplexing panel	Schloss et al. 2009; Anslan et al. 2017

Vsearch was used for d*e-novo* and reference based chimera filtering	Rognes et al. 2016
UNITE 7.2 data set served as a reference database	Kõljalg et al. 2013
Full length ITS reads without conservative regions (18S and 28S; primer binding sites) were extracted using ITSx software	Bengtsson-Palme et al. 2013
Using UPARSE, sequences were clustered to OTUs at 98% sequence similarity. Global singletons (clusters with only one sequence) were removed during clustering process (minsize = 2).	Edgar 2013
Taxonomy was assigned to representative UPARSE sequences using blastn algorithm against UNITE 7.2 database (e-value = 0.001, word_size = 7, reward = 1, penalty = -1, gapopen = 1, gapextend = 2)	Camacho et al. 2009; Kõljalg et al. 2013

Table S4. Characteristics of functional guilds and taxonomic groups used as dependent variables.

Variable	Type	Transformation	Comments
**Main fungal variables**			
total number of sequences	continuous	sqrt/log10 for calculating richness residuals	Total number of quality-filtered eukaryote ITS sequences
proportion of fungal sequences	continuous	log-ratio	proportion of fungi among eukaryotes based on relative sequence abundance
proportion of EcM fungal sequences	continuous	log-ratio	proportion of EcM fungi among eukaryotes based on relative sequence abundance
proportion of AM fungal sequences	continuous	log-ratio	proportion of AM fungi among eukaryotes based on relative sequence abundance
number of OTUs	continuous	excluded	Sum of all OTUs based on 98% sequence similarity threshold
Fungi OTU richness	continuous	mean of residuals of sqrt-transformed sequencing depth and log10-transformed total number of sequences	number of all fungal OTUs based on 98% sequence similarity threshold
Fungi Shannon index	continuous	mean of residuals of sqrt-transformed sequencing depth and log10-transformed total number of sequences	Shannon diversity index based on sequence abundances of all fungal OTUs
**Fungal functional groups**			
ectomycorrhizal fungi	continuous	mean of residuals of sqrt-transformed sequencing depth and log10-transformed total number of sequences	OTUs assigned to ectomycorrhizal guild based on primary annotation and annotated Species Hypotheses as such
arbuscular mycorrhizal fungi	continuous	mean of residuals of sqrt-transformed sequencing depth and log10-transformed total number of sequences	OTUs assigned to Glomeromycota exclusively (we also consider certain Endogonomycetes as AM, but the majority in this study probably do not act as such)
root endophytes	continuous	mean of residuals of sqrt-transformed sequencing depth and log10-transformed total number of sequences	OTUs assigned to root endophytes guild based on primary or secondary annotation as such

leaf pathogens	continuous	mean of residuals of sqrt-transformed sequencing depth and log10-transformed total number of sequences	OTUs assigned to leaf/fruit/seed pathogen guild based on primary or secondary annotation as such
wood pathogens	continuous	mean of residuals of sqrt-transformed sequencing depth and log10-transformed total number of sequences	OTUs assigned to wood pathogen guild based on primary or secondary annotation as such
animal parasites	continuous	mean of residuals of sqrt-transformed sequencing depth and log10-transformed total number of sequences	OTUs assigned to animal parasite guild based on primary or secondary annotation as such
opportunistic human pathogens	continuous	mean of residuals of sqrt-transformed sequencing depth and log10-transformed total number of sequences	OTUs assigned to opportunistic human pathogens (except Mortierella) based on annotation as such
mycoparasites	continuous	mean of residuals of sqrt-transformed sequencing depth and log10-transformed total number of sequences	OTUs assigned to mycoparasite guild based on primary or secondary annotation as such
dung saprotrophs	continuous	mean of residuals of sqrt-transformed sequencing depth and log10-transformed total number of sequences	OTUs assigned to dung saprotroph guild based on primary or secondary annotation as such
litter saprotrophs	continuous	mean of residuals of sqrt-transformed sequencing depth and log10-transformed total number of sequences	OTUs assigned to litter saprotroph guild based on primary or secondary annotation as such
wood saprotrophs	continuous	mean of residuals of sqrt-transformed sequencing depth and log10-transformed total number of sequences	OTUs assigned to wood saprotroph guild based on primary or secondary annotation as such
white-rot decomposers	continuous	mean of residuals of sqrt-transformed sequencing depth and log10-transformed total number of sequences	OTUs assigned to white-rot decay agents based on annotation as such
total saprotrophs	continuous	mean of residuals of sqrt-transformed sequencing depth and log10-transformed total number of sequences	OTUs assigned to all saprotroph guilds based on primary or secondary annotation as such
yeasts	continuous	mean of residuals of sqrt-transformed sequencing depth and log10-transformed total number of sequences	OTUs assigned to yeast life form exclusively (mostly Pucciniomycotina, Saccharomycetes, Tremellomycetes)
dimorphic yeasts	continuous	mean of residuals of sqrt-transformed sequencing depth and log10-transformed total number of sequences	OTUs assigned to dimorphic yeast (alternate yeast and filamentous stages) life form exclusively (mostly Chaetothyriales, Pucciniomycotina, Tremellomycetes)
**Fungal taxonomic groups**			
Aphelidiomycota	continuous	mean of residuals of sqrt-transformed sequencing depth and log10-transformed total number of sequences	aphelids, mostly unicellular parasites
Archaeorhizomycetes	continuous	mean of residuals of sqrt-transformed sequencing depth and log10-transformed total number of sequences	difficult-to-culture group of soil-inhabiting, saprotrophic Taphrinomycotina of ascomycetes; erroneously considered as root-

			associated; abundance may be somewhat underestimated by primer bias
Capnodiales	continuous	mean of residuals of sqrt-transformed sequencing depth and log10-transformed total number of sequences	ascomycetous microfungi with saprotrophic, plant pathogenic and endophytic lifestyles
Pleosporales	continuous	mean of residuals of sqrt-transformed sequencing depth and log10-transformed total number of sequences	ascomycetous microfungi with saprotrophic, plant pathogenic and endophytic lifestyles
Venturiales	continuous	mean of residuals of sqrt-transformed sequencing depth and log10-transformed total number of sequences	ascomycetous microfungi with saprotrophic, plant pathogenic and endophytic lifestyles
Chaetothyriales	continuous	mean of residuals of sqrt-transformed sequencing depth and log10-transformed total number of sequences	ascomycetous microfungi with saprotrophic, plant pathogenic and root endophytic lifestyles; many dimorphic yeasts
Eurotiales	continuous	mean of residuals of sqrt-transformed sequencing depth and log10-transformed total number of sequences	ascomycetous microfungi with saprotrophic, plant pathogenic and root endophytic lifestyles; most are molds, but Elaphomyces spp. are ectomycorrhizal
Onygenales	continuous	mean of residuals of sqrt-transformed sequencing depth and log10-transformed total number of sequences	ascomycetous microfungi with saprotrophic (keratinolytic) and animal parasitic lifestyle; including human pathogens that are uncommon in the dataset
Geoglossales	continuous	mean of residuals of sqrt-transformed sequencing depth and log10-transformed total number of sequences	ascomycetous macrofungi with saprotrophic lifestyle; mostly in grasslands
Helotiales	continuous	mean of residuals of sqrt-transformed sequencing depth and log10-transformed total number of sequences	ascomycetous microfungi and macrofungi with saprotrophic, plant pathogenic, root endophytic, ericoid mycorrhizal and ectomycorrhizal lifestyles; many exclusively asexual
Thelebolales	continuous	mean of residuals of sqrt-transformed sequencing depth and log10-transformed total number of sequences	ascomycetous microfungi with saprotrophic lifestyle
Orbiliomycetes	continuous	mean of residuals of sqrt-transformed sequencing depth and log10-transformed total number of sequences	ascomycetous microfungi with saprotrophic and nematophagous lifestyles
Pezizales	continuous	mean of residuals of sqrt-transformed sequencing depth and log10-transformed total number of sequences	ascomycetous micro- and macrofungi with saprotrophic, foliar endophytic and ectomycorrhizal lifestyles; most taxa ectomycorrhizal; commonly pyrophilous and alkaliphilous
Saccharomycetales	continuous	mean of residuals of sqrt-transformed sequencing depth and log10-transformed total number of sequences	ascomycetous yeasts with saprotrophic lifestyle; many opportunistic human pathogens
Chaetosphaeriales	continuous	mean of residuals of sqrt-transformed sequencing depth	ascomycetous microfungi with saprotrophic lifestyle

		and log10-transformed total number of sequences	
Coniochaetales	continuous	mean of residuals of sqrt-transformed sequencing depth and log10-transformed total number of sequences	ascomycetous microfungi with saprotrophic, plant pathogenic and endophytic lifestyles
Glomerellales	continuous	mean of residuals of sqrt-transformed sequencing depth and log10-transformed total number of sequences	ascomycetous microfungi with mostly plant pathogenic and saprotrophic lifestyles
Hypocreales	continuous	mean of residuals of sqrt-transformed sequencing depth and log10-transformed total number of sequences	ascomycetous microfungi with saprotrophic, plant pathogenic and mycoparasitic and animal parasitic lifestyles
Microascales	continuous	mean of residuals of sqrt-transformed sequencing depth and log10-transformed total number of sequences	ascomycetous microfungi with saprotrophic and plant pathogenic lifestyles
Myrmecridiales	continuous	mean of residuals of sqrt-transformed sequencing depth and log10-transformed total number of sequences	ascomycetous microfungi with saprotrophic lifestyle
Sordariales	continuous	mean of residuals of sqrt-transformed sequencing depth and log10-transformed total number of sequences	ascomycetous microfungi with saprotrophic, plant pathogenic and endophytic lifestyles; two small ectomycorrhizal lineages
Xylariales	continuous	mean of residuals of sqrt-transformed sequencing depth and log10-transformed total number of sequences	ascomycetous microfungi with saprotrophic, plant pathogenic and endophytic lifestyles
Basidiobolomycota	continuous	mean of residuals of sqrt-transformed sequencing depth and log10-transformed total number of sequences	microfungi with hypervariable lifestyle; mostly various saprotrophs and animal pathogens, including opportunistic human pathogens
Agaricales NM	continuous	mean of residuals of sqrt-transformed sequencing depth and log10-transformed total number of sequences	basidiomycetous macrofungi with mostly saprotrophic lifestyle, including several white rot agents; ectomycorrhizal groups excluded for separate analysis
Auriculariales	continuous	mean of residuals of sqrt-transformed sequencing depth and log10-transformed total number of sequences	basidiomycetous macrofungi with saprotrophic lifestyle, including several white rot agents
Ceratobasidiaceae	continuous	mean of residuals of sqrt-transformed sequencing depth and log10-transformed total number of sequences	basidiomycetous microfungi with mostly saprotrophic and nectrotrophic-pathogenic lifestyle; includes two small ectomycorrhizal groups
Hymenochaetales	continuous	mean of residuals of sqrt-transformed sequencing depth and log10-transformed total number of sequences	basidiomycetous macrofungi with mostly wood pathogenic and saprotrophic lifestyle causing white-rot; Coltricia is ectomycorrhizal
Polyporales	continuous	mean of residuals of sqrt-transformed sequencing depth and log10-transformed total number of sequences	basidiomycetous macrofungi with mostly wood pathogenic and saprotrophic lifestyle causing white-rot or brown rotl
Sebacinales NM	continuous	mean of residuals of sqrt-transformed sequencing depth and log10-transformed total number of sequences	basidiomycetous microfungi with mostly saprotrophic and root endophytic and ericoid mycorrhizal lifestyles; three ectomycorrhizal

			groups excluded for separate analysis
Trechisporales	continuous	mean of residuals of sqrt-transformed sequencing depth and log10-transformed total number of sequences	basidiomycetous macrofungi with saprotrophic lifestyle causing white-rot; sometimes mistakenly considered ectomycorrhizal
Tremellodendropsi-dales	continuous	mean of residuals of sqrt-transformed sequencing depth and log10-transformed total number of sequences	basidiomycetous macrofungi and microfungi with saprotrophic and ectomycorrhizal lifestyles that are poorly documented
Agaricostilbomycetes	continuous	mean of residuals of sqrt-transformed sequencing depth and log10-transformed total number of sequences	basidiomycetous dimorphic yeasts with mostly saprotrophic and mycoparasitic lifestyles
Geminibasidiomycetes	continuous	mean of residuals of sqrt-transformed sequencing depth and log10-transformed total number of sequences	basidiomycetous extremophilic saprotrophs
GS27	continuous	mean of residuals of sqrt-transformed sequencing depth and log10-transformed total number of sequences	basidiomycetous monophyletic group with no information on biology
Microbotryomycetes	continuous	mean of residuals of sqrt-transformed sequencing depth and log10-transformed total number of sequences	basidiomycetous yeasts with saprotrophic and plant pathogenic lifestyles
Cystofilobasidiales	continuous	mean of residuals of sqrt-transformed sequencing depth and log10-transformed total number of sequences	basidiomycetous yeasts with saprotrophic and mycoparasitic lifestyles
Filobasidiales	continuous	mean of residuals of sqrt-transformed sequencing depth and log10-transformed total number of sequences	basidiomycetous yeasts with saprotrophic lifestyles
Tremellales	continuous	mean of residuals of sqrt-transformed sequencing depth and log10-transformed total number of sequences	basidiomycetous dimorphic yeasts and yeasts with saprotrophic and animal parasitic lifestyles
Trichosporonales	continuous	mean of residuals of sqrt-transformed sequencing depth and log10-transformed total number of sequences	basidiomycetous dimorphic yeasts and yeasts with saprotrophic and mycoparasitic lifestyles
Blastocladiomycota	continuous	mean of residuals of sqrt-transformed sequencing depth and log10-transformed total number of sequences	zoosporic saprotrophs, algal parasites and plant pathogens
Chytridiomycetes	continuous	mean of residuals of sqrt-transformed sequencing depth and log10-transformed total number of sequences	zoosporic saprotrophs and animal parasites
Lobulomycetes	continuous	mean of residuals of sqrt-transformed sequencing depth and log10-transformed total number of sequences	zoosporic pollen saprotrophs and algal parasites
Rhizophlyctidomycetes	continuous	mean of residuals of sqrt-transformed sequencing depth and log10-transformed total number of sequences	zoosporic saprotrophs and algal parasites
Spizellomycetes	continuous	mean of residuals of sqrt-transformed sequencing depth	zoosporic pollen saprotrophs and algal parasites and plant pathogens

		and log10-transformed total number of sequences	
Archaeosporales	continuous	mean of residuals of sqrt-transformed sequencing depth and log10-transformed total number of sequences	arbuscular mycorrhizal
Diversisporales	continuous	mean of residuals of sqrt-transformed sequencing depth and log10-transformed total number of sequences	arbuscular mycorrhizal
Glomerales	continuous	mean of residuals of sqrt-transformed sequencing depth and log10-transformed total number of sequences	arbuscular mycorrhizal
GS01	continuous	mean of residuals of sqrt-transformed sequencing depth and log10-transformed total number of sequences	monophyletic unicellular early diverging group with no information on biology
Monoblepharomycota	continuous	mean of residuals of sqrt-transformed sequencing depth and log10-transformed total number of sequences	zoosporic saprotrophs and algal parasites
Mortierellomycetes	continuous	mean of residuals of sqrt-transformed sequencing depth and log10-transformed total number of sequences	zygomycetous molds
Endogonomycetes	continuous	mean of residuals of sqrt-transformed sequencing depth and log10-transformed total number of sequences	zygomycetous saprotrophs, arbuscular mycorrhizal and ectomycorrhizal fungi. In this study, none of the taxa were included in AM guild
Mucoromycetes	continuous	mean of residuals of sqrt-transformed sequencing depth and log10-transformed total number of sequences	zygomycetous molds, sometimes opportunistic human pathogens
Umbelopsidomycetes	continuous	mean of residuals of sqrt-transformed sequencing depth and log10-transformed total number of sequences	zygomycetous molds
Neocallimastigomycota	continuous	mean of residuals of sqrt-transformed sequencing depth and log10-transformed total number of sequences	zoosporic anaerobic or microaerophilic endosymbionts of animals
Olpidiomycota	continuous	mean of residuals of sqrt-transformed sequencing depth and log10-transformed total number of sequences	zoosporic plant pathogens and animal parasites
Rozellomycota	continuous	mean of residuals of sqrt-transformed sequencing depth and log10-transformed total number of sequences	unicellular aflagellate animal parasites and algal parasites and probably necrotrophic saprotrophs
Zoopagomycota	continuous	mean of residuals of sqrt-transformed sequencing depth and log10-transformed total number of sequences	zygomycetous animal parasites and mycoparasites
unknown fungi	continuous	mean of residuals of sqrt-transformed sequencing depth and log10-transformed total number of sequences	various group of divergent fungi that cannot be reliably placed to any phylum, most probably representing early-diverging lineages

**Molds**			
Pezizomycotina mold proportion	continuous	log-ratio	proportion of Pezizomycotina molds (mostly Aspergillaceae, Trichocomaceae) relative to total number of sequences
Pezizomycotina mold max. abundance	continuous	log-ratio	maximum relative abundance of any Pezizomycotina mold (mostly Aspergillaceae, Trichocomaceae) OTU relative to total number of sequences in a sample
Mortierellales mold proportion	continuous	log-ratio	proportion of Mortierellales molds relative to total number of sequences
Mortierellales mold max. abundance	continuous	log-ratio	maximum relative abundance of any Mortierellales mold OTU relative to total number of sequences in a sample
Mucorales mold proportion	continuous	log-ratio	proportion of Mucorales molds relative to total number of sequences
Mucorales mold max. abundance	continuous	log-ratio	maximum relative abundance of any Mucorales mold OTU relative to total number of sequences in a sample
Umbelopsidales mold proportion	continuous	log-ratio	proportion of Umbelopsidales molds relative to total number of sequences
Umbelopsidales mold max. abundance	continuous	log-ratio	maximum relative abundance of any Umbelopsidales mold OTU relative to total number of sequences in a sample
**Ectomycorrhizal exploration types**			
contact	continuous	log-ratio	taxa that produce very little mycelium emanating from root tips
long-distance	continuous	log-ratio	taxa that produce long, differentiated rhizomorphs emanating from root tips
mat	continuous	log-ratio	taxa that produce abundant short rhizomorphs and soil-binding mycelium emanating from root tips
medium-distance fringe	continuous	log-ratio	taxa that produce hairy rhizomorphs and abundant mycelium in a short distance
medium-distance smooth	continuous	log-ratio	taxa that produce smooth rhizomorphs in a short distance
short-distance delicate	continuous	log-ratio	taxa that produce no rhizomorphs but little to abundant mycelium with thin hyphae and cell walls
short-distance coarse	continuous	log-ratio	taxa that produce no rhizomorphs but little to abundant mycelium with thick hyphae and cell walls
**Ectomycorrhizal fungal lineages**			
/albatrellus	continuous	mean of residuals of sqrt-transformed sequencing depth and log10-transformed total number of sequences	basidiomycetous lineage comprising taxa with resupinate (Byssoporia), sequestrate (Mycolevis) and boletoid (Albatrellus) fruitbodies; mostly found in coniferous forests
/amanita	continuous	mean of residuals of sqrt-transformed sequencing depth	basidiomycetous lineage comprising taxa (Amanita) with mostly

		and log10-transformed total number of sequences	agaricoid fruitbodies; contact exploration type
/amphinema-tylospora	continuous	mean of residuals of sqrt-transformed sequencing depth and log10-transformed total number of sequences	basidiomycetous lineage comprising taxa with resupinate (Amphinema, Tylospora) fruitbodies; characteristic of coniferous forests; MDF and SDD exploration types
/boletopsis	continuous	mean of residuals of sqrt-transformed sequencing depth and log10-transformed total number of sequences	basidiomycetous lineage comprising taxa with boletoid (Boletopsis spp.) fruitbodies; characteristic of coniferous forests; mat exploration type
/boletus	continuous	mean of residuals of sqrt-transformed sequencing depth and log10-transformed total number of sequences	basidiomycetous lineage comprising taxa with boletoid (Boletus, Xerocomus, Tylopilus, Leccinum, etc.) and sequestrate (Octaviania and others) fruitbodies; LD exploration type
/byssocorticium	continuous	excluded	basidiomycetous lineage comprising taxa with resupinate (Byssocorticium) fruitbodies; MDF exploration type
/cantharellus	continuous	mean of residuals of sqrt-transformed sequencing depth and log10-transformed total number of sequences	basidiomycetous lineage comprising taxa with agaricoid (Cantharellus), hydnoid (Hydnum) and resupinate (Sistotrema p. parte) fruitbodies
/cenococcum	continuous	mean of residuals of sqrt-transformed sequencing depth and log10-transformed total number of sequences	ascomycetous lineage comprising taxa with no sexual state; ubiquitous; SDC exploration type
/ceratobasidium2	continuous	excluded	basidiomycetous lineage comprising taxa with resupinate (Ceratobasidium p. parte) fruitbodies; SDC exploration type
/clavariadelphus	continuous	excluded	basidiomycetous lineage comprising taxa with large clavarioid (Clavariadelphus p. parte) fruitbodies; characteristic of coniferous forests; mat exploration type
/clavulina	continuous	mean of residuals of sqrt-transformed sequencing depth and log10-transformed total number of sequences	basidiomycetous lineage comprising taxa with clavarioid (Clavulina) and resupinate (Membranomyces) fruitbodies; contact exploration type
/coltricia	continuous	excluded	basidiomycetous lineage comprising taxa with boletoid-polyporoid (Coltricia) fruitbodies; SDC exploration type
/cortinarius	continuous	mean of residuals of sqrt-transformed sequencing depth and log10-transformed total number of sequences	basidiomycetous lineage comprising taxa with agaricoid and sequestrate (Cortinarius) fruitbodies; the most diverse group based on fruit-body-based descriptions; MDF exploration type
/elaphomyces	continuous	mean of residuals of sqrt-transformed sequencing depth and log10-transformed total number of sequences	ascomycetous lineage comprising taxa with sequestrate hypogeous (Elaphomyces) fruitbodies; characteristic of coniferous forests; SDD exploration type

/endogone1	continuous	excluded	zygomycetous lineage comprising taxa with sequestrate fruitbodies (Jimgerdemannia); characteristic of coniferous forests; SDD exploration type
/entoloma	continuous	mean of residuals of sqrt-transformed sequencing depth and log10-transformed total number of sequences	basidiomycetous lineage comprising taxa with agaricoid (Entoloma p. parte) fruitbodies; MDS exploration type
/galactinia	continuous	mean of residuals of sqrt-transformed sequencing depth and log10-transformed total number of sequences	ascomycetous lineage comprising taxa with cup-shaped and sequestrate (Peziza subgen Galactinia) fruitbodies; contact exploration type
/genea-humaria	continuous	mean of residuals of sqrt-transformed sequencing depth and log10-transformed total number of sequences	ascomycetous lineage comprising taxa with cup-shaped (Humaria) and sequestrate (Genea, etc.) fruitbodies; SDC exploration type
/geopora	continuous	mean of residuals of sqrt-transformed sequencing depth and log10-transformed total number of sequences	ascomycetous lineage comprising taxa with cup-shaped and sequestrate (Geopora) fruitbodies; SDC exploration type
/hebeloma-alnicola	continuous	mean of residuals of sqrt-transformed sequencing depth and log10-transformed total number of sequences	basidiomycetous lineage comprising taxa with agaricoid (Hebeloma, Alnicola, etc.) and sequestrate (Hymenogaster) fruitbodies
/helotiales1	continuous	mean of residuals of sqrt-transformed sequencing depth and log10-transformed total number of sequences	ascomycetous lineage comprising taxa with unknown fruitbody type; SDD exploration type
/hydnellum-sarcodon	continuous	mean of residuals of sqrt-transformed sequencing depth and log10-transformed total number of sequences	basidiomycetous lineage comprising taxa with hydnoid (Hydnellum, Sarcodon) fruitbodies; characteristic of coniferous forests; mat exploration type
/hydnotrya	continuous	mean of residuals of sqrt-transformed sequencing depth and log10-transformed total number of sequences	ascomycetous lineage comprising taxa with sequestrate (Hydnotrya) fruitbodies; contact exploration type
/hygrophorus	continuous	mean of residuals of sqrt-transformed sequencing depth and log10-transformed total number of sequences	basidiomycetous lineage comprising taxa with agaricoid (Hygrophorus) fruitbodies; contact exploration type
/hysterangium	continuous	mean of residuals of sqrt-transformed sequencing depth and log10-transformed total number of sequences	basidiomycetous lineage comprising taxa with sequestrate (Hysterangium, etc.) fruitbodies; mat exploration type
/inocybe	continuous	mean of residuals of sqrt-transformed sequencing depth and log10-transformed total number of sequences	basidiomycetous lineage comprising taxa with agaricoid (Inocybe, Mallocybe) fruitbodies; one of the most species-rich groups; SDD exploration type
/laccaria	continuous	mean of residuals of sqrt-transformed sequencing depth and log10-transformed total number of sequences	basidiomycetous lineage comprising taxa with agaricoid (Laccaria) fruitbodies; pioneer strategy; SDD exploration type
/leotia	continuous	excluded	ascomycetous lineage comprising taxa with clavarioid (Leotia) fruitbodies; SDD exploration type

/marcelleina-peziza gerardii	continuous	mean of residuals of sqrt-transformed sequencing depth and log10-transformed total number of sequences	ascomycetous lineage comprising taxa with mostly sequestrate (Hydnobolites, Delastria, etc.) fruitbodies
/meliniomyces	continuous	mean of residuals of sqrt-transformed sequencing depth and log10-transformed total number of sequences	ascomycetous lineage comprising taxa with no fruitbodies (Hyaloscypha finlandica, H. bicolor); SDD exploration type
/otidea	continuous	mean of residuals of sqrt-transformed sequencing depth and log10-transformed total number of sequences	ascomycetous lineage comprising taxa with elongated cup-shaped (Otidea) fruitbodies; SDC exploration type
/pachyphloeus-amylascus	continuous	mean of residuals of sqrt-transformed sequencing depth and log10-transformed total number of sequences	ascomycetous lineage comprising taxa with sequestrate (Pachyphlodes, etc.) and disk-shaped (Scabropezia) fruit-bodies; SDC exploration type
/paralyophyllum	continuous	mean of residuals of sqrt-transformed sequencing depth and log10-transformed total number of sequences	basidiomycetous lineage comprising taxa with agaricoid (Lyophyllum s. stricto) fruitbodies; MDF exploration type
/paxillus-gyrodon	continuous	mean of residuals of sqrt-transformed sequencing depth and log10-transformed total number of sequences	basidiomycetous lineage comprising taxa with agaricoid (Paxillus), boletoid (Gyrodon) and sequestrate (Melanogaster) fruitbodies; LD exploration type
/phaeocollybia	continuous	excluded	basidiomycetous lineage comprising taxa with agaricoid (Phaeocollybia) fruitbodies; MDF exploration type
/phellodon-bankera	continuous	mean of residuals of sqrt-transformed sequencing depth and log10-transformed total number of sequences	basidiomycetous lineage comprising taxa with boletoid (Bankera) and hydnoid (Phellodon) fruitbodies; characteristic of coniferous forests; mat exploration type
/piloderma	continuous	mean of residuals of sqrt-transformed sequencing depth and log10-transformed total number of sequences	basidiomycetous lineage comprising taxa with resupinate (Piloderma, Tretomyces) fruitbodies; characteristic of coniferous forests; MDF exploration type
/pisolithus-scleroderma	continuous	mean of residuals of sqrt-transformed sequencing depth and log10-transformed total number of sequences	basidiomycetous lineage comprising taxa with sequestrate (Pisolithus, Scleroderma) and boletoid (Gyroporus) fruitbodies; LD exploration type
/pseudotomentella	continuous	mean of residuals of sqrt-transformed sequencing depth and log10-transformed total number of sequences	basidiomycetous lineage comprising taxa with resupinate (Pseudotomentella) fruitbodies; MDS exploration type
/pulvinula	continuous	mean of residuals of sqrt-transformed sequencing depth and log10-transformed total number of sequences	ascomycetous lineage comprising taxa with cup-shaped (Pulvinula) fruitbodies; SDD exploration type
/pustularia	continuous	mean of residuals of sqrt-transformed sequencing depth and log10-transformed total number of sequences	ascomycetous lineage comprising taxa with cup-shaped (Pustularia patavina) fruitbodies; SDC exploration type
/ramaria-gautieria	continuous	mean of residuals of sqrt-transformed sequencing depth and log10-transformed total number of sequences	basidiomycetous lineage comprising taxa with large clavarioid (Ramaria s. stricto) and sequestrate (Gautieria) fruitbodies;

			characteristic of coniferous forests; mat exploration type
/rhodoscypha	continuous	mean of residuals of sqrt-transformed sequencing depth and log10-transformed total number of sequences	ascomycetous lineage comprising taxa with cup-shaped (Rhodoscypha) fruitbodies
/russula-lactarius	continuous	mean of residuals of sqrt-transformed sequencing depth and log10-transformed total number of sequences	basidiomycetous lineage comprising taxa with agaricoid and sometimes sequestrate fruitbodies (Russula, Lactarius, Lactifluus)
/sebacina	continuous	mean of residuals of sqrt-transformed sequencing depth and log10-transformed total number of sequences	basidiomycetous lineage comprising taxa with resupinate (Sebacina) and tremelloid (Helvellosebacina) fruitbodies; contact or SDD exploration types
/serendipita1	continuous	mean of residuals of sqrt-transformed sequencing depth and log10-transformed total number of sequences	basidiomycetous lineage comprising taxa with no known fruitbodies; SDD exploration type
/serendipita2	continuous	mean of residuals of sqrt-transformed sequencing depth and log10-transformed total number of sequences	basidiomycetous lineage comprising taxa with no known fruitbodies; SDD exploration type
/sordariales2	continuous	mean of residuals of sqrt-transformed sequencing depth and log10-transformed total number of sequences	ascomycetous lineage comprising taxa with no known fruitbodies; found only with Salix; SDD exploration type
/sphaerosporella	continuous	mean of residuals of sqrt-transformed sequencing depth and log10-transformed total number of sequences	ascomycetous lineage comprising taxa with cup-shaped (Sphaerosporella, Trichophaea p. parte) fruitbodies; SDC exploration type
/suillus-rhizopogon	continuous	mean of residuals of sqrt-transformed sequencing depth and log10-transformed total number of sequences	basidiomycetous lineage comprising taxa with boletoid (Suillus, Gomphidius) and sequestrate (Rhizopogon) fruitbodies; LD exploration type
/tarzetta	continuous	mean of residuals of sqrt-transformed sequencing depth and log10-transformed total number of sequences	ascomycetous lineage comprising taxa with cup-shaped (Tarzetta) fruitbodies; contact exploration type
/terfezia-peziza depressa	continuous	mean of residuals of sqrt-transformed sequencing depth and log10-transformed total number of sequences	ascomycetous lineage comprising taxa with sequestrate and cup-shaped (Peziza p. parte) fruitbodies; SDC exploration type
/tomentella-thelephora	continuous	mean of residuals of sqrt-transformed sequencing depth and log10-transformed total number of sequences	basidiomycetous lineage comprising taxa with resupinate (Tomentella) or clavarioid (Thelephora) fruitbodies; MDS exploration type
/tomentellopsis	continuous	mean of residuals of sqrt-transformed sequencing depth and log10-transformed total number of sequences	basidiomycetous lineage comprising taxa with resupinate (Tomentellopsis) fruitbodies; MDS exploration type
/tremellodendropsis	continuous	mean of residuals of sqrt-transformed sequencing depth and log10-transformed total number of sequences	basidiomycetous lineage comprising taxa with resupinate and clavarioid (Tremellodendropsis) fruitbodies; SDD exploration type
/tricholoma	continuous	mean of residuals of sqrt-transformed sequencing depth	basidiomycetous lineage comprising taxa with agaricoid (Tricholoma) fruitbodies; MDF exploration type

		and log10-transformed total number of sequences	
/tuber-helvella	continuous	mean of residuals of sqrt-transformed sequencing depth and log10-transformed total number of sequences	ascomycetous lineage comprising taxa with sequestrate (Tuber) and helvelloid (Helvella) fruitbodies; contact exploration type
/tulasnella1	continuous	mean of residuals of sqrt-transformed sequencing depth and log10-transformed total number of sequences	basidiomycetous lineage comprising taxa with no known fruitbody type
/wilcoxina	continuous	mean of residuals of sqrt-transformed sequencing depth and log10-transformed total number of sequences	ascomycetous lineage comprising taxa with cup-shaped (Wilcoxina, Trichophaea p. parte) fruitbodies; pioneer strategy; SDC exploration type
uncertain1	continuous	mean of residuals of sqrt-transformed sequencing depth and log10-transformed total number of sequences	ascomycetous group that may be potentially ectomycorrhizal based on islolations from EcM roots
uncertain2	continuous	mean of residuals of sqrt-transformed sequencing depth and log10-transformed total number of sequences	ascomycetous group that may be potentially ectomycorrhizal based on islolations from EcM roots

Table S5. Factors determining the abundance of molds. All effects are significant (P<0.001).

Dependent variable	Predictor (direction[1] and variation explained based on GLM, %)
Mortierellales mold relative abundance	month (6.0), soil pH (∩11.3), soil C (∩3.6), soil N (↘2.0), soil Ca (∩1.9), tMEM04 (0.8), tMEM05 (2.0), tMEM06 (2.8)
Mortierellales mold OTU max. abundance	collector (27.2), year (23.8), pH (∩6.8), EcM plant proportion (↗1.5)
Mucorales mold relative abundance	cropland (↗2.4), soil P (↗0.8), soil K (↗0.5), tMEM03 (1.2), tMEM05 (2.8), tMEM06 (3.2), gMEM03 (0.7)
Mucorales mold OTU max. abundance	year (7.2), cropland (↗4.9), bog (↗1.6)
Umbelopsidales mold relative abundance	collector (30.2), year (11.2), month (1.2), soil pH (↘21.5), *Betula*% (↗4.7), *Populus tremula*% (↗1.1), *Pinus*% (↗0.8), *Prunus padus*% (↗0.5), soil Mg (∩0.5)
Umbelopsidales mold OTU max. abundance	collector (27.4), year (13.5), month (0.8), soil pH (∩7.2), soil C (↗1.3), *Picea*% (↗0.8), soil K (↗0.8), soil N (↗0.7)
Pezizomycotina mold relative abundance	year (24.8), month (2.7), collector (2.0), vegetation age (∩1.5),
Pezizomycotina mold OTU max. abundance	collector (4.2), month (2.0), year (1.8), Mortierellales mold% (↘17.1)

[1]symbols in continuous predictors: ↗, near-linear increase; ↘, near-linear decline; ʃ, sigmoid increase; Γ, cumulative increase; ∩, unimodal

Table S6. Richness and relative abundance of ectomycorrhizal fungal lineages (dataset#4).

Lineage	OTUs	OTUs (%)	Sequences (%)	OTUs per sample±SD
/tomentella-thelephora	618	22.22	16.44	21.11±12.85
/inocybe	505	18.16	22.84	14.30±10.40
/cortinarius	367	13.20	7.31	7.36±5.80
/russula-lactarius	205	7.37	14.45	8.39±6.16
/sebacina	150	5.39	5.787	6.59±7.14
/hebeloma-alnicola	68	2.44	3.992	4.33±3.69
/cantharellus	65	2.33	0.593	0.80±1.30

/clavulina	63	2.26	2.191	1.70±1.66
/pseudotomentella	59	2.12	1.430	1.80±1.69
/piloderma	49	1.76	3.114	2.25±2.53
/tricholoma	48	1.72	0.923	0.74±1.05
/amphinema-tylospora	38	1.36	7.449	3.63±3.06
/genea-humaria	36	1.29	1.009	1.72±1.94
/tuber-helvella	33	1.18	1.247	1.76±1.93
/amanita	29	1.04	1.122	0.82±1.05
/pulvinula	26	0.93	0.118	0.25±0.67
/boletus	25	0.90	0.322	0.67±0.96
/terfezia-peziza depressa	25	0.90	0.188	0.51±0.84
/geopora	24	0.86	0.224	0.44±1.12
/otidea	23	0.83	0.263	0.45±0.76
/hygrophorus	21	0.75	0.358	0.40±0.69
/suillus-rhizopogon	20	0.72	0.244	0.50±0.88
/ramaria-gautieria	19	0.68	0.129	0.05±0.23
/meliniomyces	17	0.61	1.626	2.34±2.49
/entoloma	17	0.61	0.139	0.32±0.61
/tomentellopsis	16	0.57	0.064	0.17±0.47
/cenococcum	15	0.54	1.689	2.20±1.94
/paxillus-gyrodon	15	0.54	0.349	0.92±1.09
/wilcoxina	14	0.50	1.510	1.24±1.48
/elaphomyces	14	0.50	0.112	0.26±0.59
/hysterangium	13	0.47	0.070	0.14±0.41
/laccaria	12	0.43	0.884	1.06±1.09
/pachyphloeus-amylascus	12	0.43	0.110	0.38±0.74
/hydnellum-sarcodon	11	0.39	0.015	0.03±0.21
/tarzetta	10	0.36	0.140	0.28±0.53
/paralyophyllum	8	0.29	0.043	0.01±0.10
/pisolithus-scleroderma	7	0.25	0.284	0.27±0.65
/marcelleina-peziza gerardii	7	0.25	0.087	0.22±0.48
/serendipita1	6	0.22	0.220	0.43±0.80
/sphaerosporella	6	0.22	0.111	0.23±0.52
/pustularia	6	0.22	0.093	0.16±0.47
/serendipita2	6	0.22	0.054	0.19±0.55
/helotiales1	6	0.22	0.021	0.10±0.32
/albatrellus	6	0.22	0.006	0.02±0.17
/sordariales2	5	0.18	0.263	0.06±0.32
/galactinia	5	0.18	0.238	0.44±0.75
/byssocorticium	4	0.14	0.034	0.04±0.20
/tremellodendropsis	4	0.14	0.020	0.12±0.33
/hydnotrya	4	0.14	0.011	0.06±0.25
/phellodon-bankera	4	0.14	0.003	0.02±0.14
/phaeocollybia	3	0.11	0.005	0.01±0.12
/ceratobasidium2	3	0.11	0.005	0.03±0.17
/rhodoscypha	2	0.07	0.038	0.18±0.40
/clavariadelphus	2	0.07	0.009	<0.01±0.07
/leotia	1	0.04	0.005	0.02±0.14
/coltricia	1	0.04	0.001	<0.01±0.07
/tulasnella1	1	0.04	0.001	<0.01±0.09
/endogone1	1	0.04	<0.001	<0.01±0.04
/boletopsis	1	0.04	<0.001	<0.01±0.03

Table S7. Factors affecting richness of EcM fungal lineages (dataset#4). All effects are significant (P<0.001).

Dependent variable	MSE_{total} (random forest)	Predictors (directionality[1], MSE of random forest; variation explained based on GLM, %)

number of EcM fungal lineages	48.2	*Betula*% (Γ28.5;**18.9**), *Quercus*% (∩25.3;11.0), Ca (∩23.0;11.1), Umbelopsidales mold% (∩22.0;3.6), pH (∩21.0;6.2), Umb max (∩20.5;1.4), age (Γ20.2;5.9), EcM plant% (∩17.9;6.4), EcM plants (↗15.7;2.3)
EcM fungal richness	58.0	Mortierellales mold% (↘37.6;8.1), Ca (Γ28.9;**26.0**), *Betula*% (∩28.5;21.3), *Corylus*% (∩23.8;13.6), EcM plant% (∩22.2;13.5), N/P (↗21.5;4.7), pH (∩20.0;8.4)
relative abundance of EcM fungi	56.0	Mort% (↘58.5;**24.0**), EcM plant% (Γ40.8;3.8), Pez mold max (↘29.4;9.2), *Picea*% (↗27.1;0.6)
**EcM fungal lineages**		
albatrellus	-9.6	δ^{15}N (↘11.1;**1.3**)
/amanita	33.7	pH (↘36.1;**25.9**), Umbelopsidales mold% (26.7;-), Umb max (17.9;-), C/N (∩12.5;4.6)
/amphinema-tylospora	62.6	*Picea*% (Γ85.5;**57.4**), δ15N (↘23.4;10.2), *Pinus*% (∩22.8;3.2),C/N (∩22.1;1.0), Umbelopsidales mold% (15.1;-)
/boletus	17.9	Umbelopsidales mold% (20.2;1.3), Umb max (18.6;0.7), δ^{15}N (↘14.5;**5.9**), pH (↘13.3;0.8)
/cantharellus	23.2	Umbelopsidales mold% (20.5;0.6), C/N (∩20.5;**13.4**), δ15N (↘17.4;2.0), Umb max (14.3;1.5), *Betula*% (↗8.7;2.0)
/cenococcum	33.2	Mortierellales mold% (29.7;7.6), δ^{15}N (↘29.0;**9.0**), C/N (∩18.4;3.0), Umbelopsidales mold% (16.6;3.4)
/ceratobasidium2	18.7	Mg (↘19.2;**7.7**), C/P ratio (↗11.4;0.8)
/clavulina	22.1	C/P (↗18.2;1.2), Umbelopsidales mold% (17.4;0.9), δ^{15}N (↘17.0;0.4), P (↘16.9;**12.2**), N/P (↗16.0;1.3), C/N (↗15.5;-)
/cortinarius	39.8	*Populus tremula*% (↗20.8;8.2), C/N (Γ20.7;**16.4**), δ^{15}N (↘20.7;4.4), Umbelopsidales mold% (20.0;4.6), tMEM01 (14.7;3.5)
/entoloma	11.0	*Tilia*% (↗12.0;**2.0**)
/galactinia	43.2	pH (↗47.4;**27.6**), *Quercus*% (↗32.6;9.7), *Tilia*% (↗15.4;0.8), urban (↗13.7;0.6)
/genea-humaria	38.1	pH (↗32.5;11.3), *Quercus*% (Γ32.3;**17.5**), *Corylus*% (↗21.4;6.6), Ca (14.5;-), *Tilia*% (↗14.5;1.8)
/geopora	59.3	*Salix*% (Γ54.5;**42.3**), pH (↗31.2;7.9), δ^{15}N (↗17.0;1.6), Populus berolinensis% (↗11.3;4.6), urban (↗11.1;5.5)
/hebeloma-alnicola	52.4	pH (Γ47.5;**40.6**), *Salix*% (↗21.0;9.0), Umbelopsidales mold% (18.3;0.6), EcM plant% (∩17.6;3.4), *Picea*% (↘15.6;3.4)
/helotiales1	10.6	δ^{15}N (↘21.6;**4.7**), C/N (↗17.3;-), pH (↗12.3;1.8), *Picea*% (↗10.8;1.1)
/hydnellum-sarcodon	23.9	*Pinus*% (↗18.4;**10.8**), pH (↘11.7;-), C/N (↗11.3;1.2)
/hydnotrya	5.8	pH (↘25.5;**3.6**), *Quercus*% (↗13.6;0.5), gMEM043 (13.3;), *Tilia*% (↗10.6;0.5)
/hygrophorus	23.4	*Picea*% (Γ33.2;**10.6**), C/P (↗14.5;3.9), pH (↘13.6;0.6), δ^{15}N (↘13.0;-)
/hysterangium	7.0	cdMEM4 (14.6;**2.8**), cdMEM5 (14.2;2.3), *Picea*% (↗10.0;0.7)
/inocybe	64.2	pH (∩50.7;**44.4**), *Corylus*% (Γ26.6;14.3), Ca (↗24.5;3.6), age (↗24.3;8.3), Mortierellales mold% (22.7;3.7), *Tilia*% (Γ21.0;2.0)
/laccaria	28.0	pH (∩32.6;**16.7**), C/N (↘24.4;1.4), δ^{13}C (U 24.3;3.5)
/marcelleina-peziza gerardii	22.1	*Tilia*% (Γ22.1;6.2), Ca (↗20.1;**7.0**), pH (↗15.6;-), *Corylus*% (Γ15.2;4.6), N/P (↗12.2;3.3)
/meliniomyces	45.9	*Populus tremula*% (↗41.2;6.3), age (↘38.5;**13.5**), Umbelopsidales mold% (28.4;-), Umb max (19.4;-), *Picea*% (↗16.1;10.2)
/otidea	22.1	*Quercus*% (↗35.8;**9.5**), age (↗17.0;2.8), Ca (↗16.5;-)
/pachyphloeus-amylascus	20.6	*Corylus*% (↗14.3;**6.4**), ntMEM5 (14.0;), *Tilia*% (↗12.0;0.9), *Quercus*% (↗11.7;2.5)
/paxillus-gyrodon	30.4	*Corylus*% (↗21.1;6.4), *Salix*% (↘19.5;3.6), pH (↗19.1;**13.0**), Ca (↗18.4;1.5), *Quercus*% (∩14.2;3.6)
/phellodon-bankera	16.6	C/N ratio (↗17.6;**10.8**), Mg (↘10.2;1.7)
/piloderma	42.0	δ^{15}N (↘41.8;**32.0**), C/N (∩26.5;3.1), Umbelopsidales mold% (20.8;-), pH (↘18.9;0.6), *Picea*% (∩16.4;1.6)
/pisolithus-scleroderma	40.3	*Quercus*% (↗49.8;**23.9**), *Tilia*% (↗26.4;4.5), village (↗13.4;4.3), Ca (↗13.4;-)
/pseudotomentella	29.8	*Picea*% (↗30.1;**13.2**), C/N (∩20.3;4.8), δ^{15}N (↘18.0;-), *Corylus*% (↗14.3;4.3)

/pulvinula	41.8	*Salix*% (↗44.5;**33.4**), pH (↗19.6;2.5)
/pustularia	23.2	*Salix*% (↗23.6;**13.7**), pH (↗19.0;2.5)
/ramaria-gautieria	-4.9	-
/rhodoscypha	25.6	*Picea*% (↗48.5;**17.8**), pH (∩19.2;3.6), C/N (↘15.0;-)
/russula-lactarius	56.3	pH (∩41.1;**43.8**), Umbelopsidales mold% (37.0;-), *Betula*% (∩30.4;13.0), δ^{15}N (↘25.7;5.2), P (∩21.7;1.4), Umb max (19.8;1.2), age (∩18.4;3.3)
/sebacina	64.8	Mortierellales mold% (35.2;8.4), pH (Γ32.8;**31.1**), Ca (↗31.8;1.0), *Tilia*% (∩30.6;2.4), age (↗24.5;6.5), N/P (↗23.8;**26.7**), *Corylus*% (↗22.1;3.8)
/serendipita1	31.6	*Salix*% (↗41.3;**12.8**), age (↘39.0;5.0), *Betula*% (↗21.3;5.2), δ^{13}C (↘18.6;0.2)
/serendipita2	20.1	C/N (∩20.7;**6.9**), δ^{15}N (↘16.5;3.1), *Picea*% (∩13.7;1.0)
/sordariales2	27.6	*Salix*% (↗27.8;**15.8**), cdMEM10 (17.5;-)
/sphaerosporella	21.4	pH (↗21.1;13.5)
/suillus-rhizopogon	34.8	*Pinus*% (↗57.6;**21.3**), *Larix*% (↗20.8;0.6), C/N (↗17.1;-)
/tarzetta	17.0	pH (↗16.1;**6.8**), *Tilia*% (↗14.3;-)
/terfezia-peziza depressa	28.7	δ^{15}N (↗20.2;**14.4**), pH (↗17.1;3.0), *Quercus*% (↗15.0;2.1), *Salix*% (↗14.9;1.9), C/N (↘11.9;-), *Tilia*% (↗10.4;1.0)
/tomentella-thelephora	62.7	Mortierellales mold% (44.8;8.8), pH (∩39.0;**41.3**), *Betula*% (Γ23.9;18.9), Ca (∩23.3;9.2), P (∩22.4;12.6), *Quercus*% (Γ20.8;7.2)
/tomentellopsis	17.6	C/P (↗17.2;2.7), C/N (↗14.3;**8.2**), N/P (↗10.8;-)
/tremellodendropsis	8.2	cdMEM21 (12.2;1.4), age (↗10.0;**2.7**)
/tricholoma	23.8	age (↗13.3;**5.6**), *Betula*% (↗12.9;2.5), Mortierellales mold% (12.6;5.9)
/tuber-helvella	54.3	pH (↗48.4;**44.4**), Umbelopsidales mold% (20.0;0.7), Ca (↗15.0;-)
/tulasnella1	-2.8	psMEM29 (10.9;3.6)
/uncertain1	-1.0	gMEM017 (15.4;0.5)
/uncertain2	28.0	pH (∩23.5;14.6), *Betula*% (∩19.5;6.8), *Picea*% (∩19.3;1.3)
/wilcoxina	53.4	*Picea*% (↗67.9;**38.6**), pH (∩21.9;5.8), C/N (∩17.7;4.2), age (↘16.9;1.8)
**Exploration types**		
contact	43.5	pH (↘34.7;**29.9**), Ca (↘16.9;0.9), age (↗14.3;1.1)
LDD_LR	27.2	*Salix*% (↘47.9;4.3), age (↗25.4;**12.3**), *Quercus*% (↗16.0;1.8)
mat_LR	0	pH (↘-;**1.3**)
MD_F_LR	50.8	δ^{15}N (↘28.7;**27.5**), *Picea*% (↗26.1;3.7), age (↘23.1;3.2), pH (↘22.6;1.1)
MD_S_LR	31.2	C/N (∩23.7;14.0), age (Γ18.4;2.8), *Alnus* (↗14.1;2.5), *Pinus*% (↘13.7;-)
SD_C_LR	16.0	*Salix*% (↗19.7;2.7), pH (↗19.3;**2.8**), age (↘19.1;-)
SD_D_LR	51.2	pH (↗43.7;**44.7**), C/N (↘19.4;2.1)
SD_TOT_LR	53.1	pH (↗50.5;**46.7**), age (↘28.1;0.5)

[1]symbols in continuous predictors: ↗, near-linear increase; ↘, near-linear decline; ʃ, sigmoid increase; Γ, cumulative increase; ∩, unimodal

Table S8. Effects of environmental variables on composition of ectomycorrhizal fungi, saprotrophs and pathogens based on AICc-based best DISTLM models. All effects are significant (P<0.001).

Dependent variable	F_{pseudo}-value	Variation explained (%)
**Ectomycorrhizal fungi (OTUs)**		
soil pH	60.5	5.3
Picea abies%	29.1	2.5
soil C/N ratio	16.4	1.4
vegetation age	14.7	1.2
soil P	9.9	0.8
soil Mg	9.5	0.8
Alnus spp.%	9.1	0.7
Populus x wettsteinii%	8.0	0.6
Betula spp.%	7.8	0.6
Tilia spp.%	7.3	0.6
**Ectomycorrhizal fungi (lineages)**		
soil pH	325.0	23.0
Picea abies%	70.3	4.7
vegetation age	39.7	2.6
soil C/N ratio	38.7	2.4
Alnus%	24.1	1.5
time	17.4	1.0
Populus x wettsteinii%	15.1	0.9
soil $\delta^{15}N$	13.8	0.8
soil Ca	11.4	0.7
soil P	10.5	0.6
**Saprotrophs (combined)**		
soil pH	150.7	11.4
Picea abies%	23.0	1.7
Umbelopsidales molds max.%	21.2	1.5
Mortierellales molds%	21.4	1.5
soil P	14.7	1.0
soil $\delta^{15}N$	12.1	0.8
Pinus sylvestris%	11.7	0.8
soil Mg	7.8	0.5
tMEM01	7.6	0.5
Prunus padus%	6.9	0.5
Fraxinus excelsior%	4.6	0.3
soil C/N ratio	4.5	0.3
**Plant pathogens (combined)**		
soil pH	80.8	6.4
soil P	20.7	1.6
Picea abies%	15.9	1.2
soil $\delta^{15}N$	13.5	1.0
Pinus sylvestris%	12.3	0.9
soil N	11.2	0.8
Tilia spp.%	9.1	0.7
soil C	7.6	0.6
Umbelopsidales molds max.%	7.5	0.6
Mortierellales molds%	5.9	0.4
Quercus robur%	5.8	0.4
tMEM02	5.4	0.4
soil Mg	5.4	0.4
Salix spp.%	4.9	0.4
EcM plant richness	4.7	0.3
tMEM01	4.3	0.3
cdMEM1	4.2	0.3
soil C/N ratio	3.7	0.3
soil K	3.3	0.2

Table S9. Niche models of 50 most frequent EcM fungal OTUs. All effects are significant (P<0.001).

Dependent variable	UNITE Species Hypothesis	MSE_{total} (random forest)	Predictors (directionality[1], MSE of random forest; variation explained based on GLM, %)
OTU00002 Tylospora asterophora	SH1427431.08FU	68.9	*Picea*% (↗61.8;**49.0**), pH (↘28.0;6.7), δ^{15}N (↘19.6;-), age (↘18.8;4.6)
OTU00007 Amphinema byssoides	SH1490949.08FU	61.2	*Picea*% (↗81.0;**50.0**), pH (∩27.9;8.5), δ^{15}N (↘18.9;3.9), *Larix*% (↗14.8;1.6)
OTU00010 Cortinarius diasemospermus	SH1405300.08FU	31.7	age (↘29.7;**9.0**), *Populus tremula*% (↗26.4;3.4), δ^{13}C (↘17.3;1.8), energy plantation (↗16.0;4.3), *Betula*% (↗14.4;1.6)
OTU00013 Membranomyces sp	SH1479373.08FU	37.9	*Picea*% (↗34.4;**18.0**), pH (∩18.2;2.9), C/N (↘17.3;1.8), δ^{15}N (↘13.0;1.6)
OTU00015 Tomentella sp	SH1390004.08FU	37.5	pH (∩28.5;**20.3**), δ^{15}N (↘25.0;4.7), *Picea*% (↗20.9;3.1), C/N (↘19.6;2.0)
OTU00018 Amanita fulva	SH1459578.08FU	50.9	pH (↘50.8;**36.8**), Umb max (18.9;0.8), Umbelopsidales mold% (17.9;-), C/N (↗17.6;2.0), δ^{15}N (↘16.8;0.8)
OTU00019 Amphinema sp	SH1490954.08FU	53.0	*Picea*% (↗50.0;**26.9**), pH (∩22.9;6.1)
OTU00025 Trichophaea aff gregaria	SH1415791.08FU	47.0	*Picea*% (↗38.7;**25.7**), C/N (↘15.7;1.7)
OTU00033 Russula firmula	SH1399861.08FU	36.9	*Picea*% (↗42.4;**16.9**), ntMEM3 (16.4;2.2), Ca (↗14.7;3.5)
OTU00035 Trichophaea gregaria	SH1415791.08FU	44.7	*Picea*% (↗53.7;**24.1**), pH (∩26.1;8.7), age (↘23.9;1.1), *Larix*% (↗14.5;0.6), Umbelopsidales mold% (13.4;0.9), C/N (↘13.1;1.6)
OTU00040 Inocybe acutofulva	SH1412557.08FU	23.2	pH (∩18.7;**7.7**), *Populus tremula*% (↗16.2;2.2), C/N (↘13.0;-), *Betula*% (↗12.5;1.6), δ^{13}C (↘12.3;0.7)
OTU00044 Russula delica	SH1373615.08FU	18.8	*Tilia*% (↗26.5;4.2), pH (∩18.9;**6.7**)
OTU00055 Cadophora finlandica	SH1385938.08FU	33.4	*Populus tremula*% (↗44.9;6.3), age (↘38.4;**10.6**), energy plantation (↗12.3;4.5)
OTU00056 Lactarius tabidus	SH1381881.08FU	30.7	pH (↘35.9;**15.9**), Umbelopsidales mold% (12.0;-); *Picea*% (↗10.0;1.8)
OTU00059 Laccaria macrocystidia	SH1411776.08FU	32.1	pH (∩34.4;**17.7**), Ca (∩25.5;6.8), C/N (↘20.3;1.2)
OTU00075 Cadophora aff finlandica	SH1385938.08FU	52.3	Age (↘35.3;11.1), *Salix*% (↗30.2;**25.1**),
OTU00077 Cadophora aff finlandica	SH1385938.08FU	35.9	Age (↘38.6;**17.9**), *Picea*% (↗15.1;2.4), *Populus tremula*% (↗14.6;-)
OTU00081 Inocybe mixtilis	SH1369422.08FU	11.2	pH (∩19.9;**7.7**), P (↘17.2;1.9), N/P (↗12.6;)
OTU00091 Tomentella aff coerulea	SH1366919.08FU	15.9	pH (∩33.6;**13.3**), δ^{15}N (↘14.2;2.5), C/N (∩13.7;-), Ca (∩11.9;2.3)
OTU00101 Cortinarius casimiri	SH1405298.08FU	31.4	δ^{15}N (↘30.8;**14.9**), Umbelopsidales mold% (20.3;0.5), *Betula*% (∩18.7;5.5), *Picea*% (∩16.0;1.3), C/N (∩14.0;1.6)
OTU00102 Inocybe aff maculata	SH1412557.08FU	17.8	pH (∩14.2;**5.9**), *Betula*% (↗13.5;2.8), *Populus tremula*% (↗10.5;0.6)
OTU00106 Tomentella aff lammiensis	SH1390007.08FU	22.1	δ^{15}N (↘23.3;**11.7**), *Picea*% (Г21.7;4.0), pH (∩14.2;4.8)
OTU00110 Inocybe posterula	SH1459516.08FU	16.1	*Picea*% (↗18.1;**6.2**), pH (∩14.2;4.5), P (↘13.6;1.8)
OTU00114 Tomentella sp	SH1366884.08FU	23.7	Ca (∩19.6;4.2), pH (∩19.0;**10.6**), N (↘14.3;1.5), C/N (↘11.5;-)
OTU00115 Cortinarius decipiens	SH1405298.08FU	27.8	*Populus tremula*% (↗36.9;8.7); *Salix*% (↗21.4;**9.3**), cdMEM8 (13.6;0.9)
OTU00117 Inocybe geophylla	SH1459519.08FU	16.8	pH (∩20.2;**9.0**), P (↘18.8;3.4), C/N (↘13.7;1.7), cdMEM5 (13.0;0.8)

OTU00119 Hymenogaster vulgaris	SH1420890.08FU	38.6	pH (↗28.5;**19.5**), Ca (∩16.8;3.7), Mg (∩14.8;0.9)
OTU00137 Meliniomyces bicolor	SH1385938.08FU	31.7	pH (↘22.6;**18.7**), Umbelopsidales mold% (15.0;-)
OTU00139 Piloderma sp	SH1404924.08FU	15.4	pH (∩17.5;**5.4**), *Quercus*% (↗10.5;1.2)
OTU00141 Inocybe maculata	SH1426217.08FU	33.8	pH (↗27.6;**18.0**), *Tilia*% (↗24.0;6.2)
OTU00143 Amphinema sp	SH1490949.08FU	47.1	*Picea*% (↗57.4;**16.9**), pH (∩26.5;10.8), Umbelopsidales mold% (17.2;1.0)
OTU00150 Inocybe sp	SH1432619.08FU	29.7	pH (∩34.0;**16.1**), P (↘16.6;3.7), Ca (∩14.3;3.3), C/N (↘14.1;-), N (↘14.0;0.8)
OTU00183 Tomentella cinereoumbrina	SH1390059.08FU	16.6	pH (∩11.8;**7.7**), *Picea*% (↗10.5;2.9)
OTU00207 Tomentella sp	SH1366866.08FU	11.5	Ca (∩14.9;4.0), *Betula*% (↗11.6;2.2), *Sorbus*% (↗11.4;3.2), pH(∩10.0;**7.8**)
OTU00210 Inocybe rimosa	SH1492224.08FU	23.5	pH (∩20.2;**8.5**), *Quercus*% (↗16.5;3.0), age (↗15.1;1.1), C/N (↘13.5;-),
OTU00215 Humaria hemisphaerica	SH1457269.08FU	13.1	*Tilia*% (∩13.2;2.2), pH (∩12.1;**5.9**)
OTU00300 Hebeloma incarnatulum	SH1420888.08FU	11.2	Plant richness (↗14.3;**2.5**), gMEM254 (13.3;0.6)
OTU00339 Tuber rufum	SH1404396.08FU	18.3	pH (↗23.1;**9.1**), Umbelopsidales mold% (11.3;-), *Quercus*% (↗10.0;1.5)
OTU00379 Hebeloma leucosarx	SH1421029.08FU	9.3	*Betula*% (↗14.6;**4.0**)
OTU00392 Inocybe melliolens	SH1492224.08FU	17.0	pH (↗20.6;**11.3**), *Quercus*% (↗10.1;1.5)
OTU00401 Sebacina sp	SH1419243.08FU	17.0	pH (↗19.0;**7.4**), Ca (∩15.5;-), *Tilia*% (↗9.3;-)
OTU00688 Tomentella sp	SH1390003.08FU	31.5	pH (↗26.1;**10.6**), N/P (↗22.6;1.2), *Tilia*% (↗16.4;3.8); P (↘15.8;1.8)
OTU00689 Helvellosebacina sp	SH1378964.08FU	19.1	Ca (↗20.4;-), pH (↗19.5;**7.5**), *Quercus*% (↗10.0;0.9)
OTU00779 Hebeloma fragilipes	SH1420886.08FU	27.3	*Populus tremula*% (↗43.6;**11.3**), *Betula*% (↗9.9;0.9)
OTU00821 Inocybe aff maculata	SH1369787.08FU	46.3	*Tilia*% (↗43.5;**9.6**), pH (↗31.6;8.3), $\delta^{13}C$ (↗17.6;3.1)
OTU01069 Melanogaster broomeanus	SH1401114.08FU	25.5	pH (↗26.8;**5.5**), *Tilia*% (↗14.8;2.5), *Corylus*% (10.1;2.7)
OTU01472 Inocybe umbrinella	SH1492227.08FU	37.9	pH (↗35.0;**13.7**), *Corylus*% (↗11.0;2.4), *Quercus*% (10.0;0.7)
OTU01890 Hebeloma quercetorum	SH1420888.08FU	27.8	*Tilia*% (↗31.5;**6.1**), pH (↗19.9;0.4), *Quercus*% (↗17.5;4.8), Mort max (12.7;4.2)
OTU02132 Tuber sp	SH1423757.08FU	36.7	*Salix*% (↗27.1;**9.9**), pH (↗21.3;3.2), *Betula*% (↗14.4;0.4), $\delta^{15}N$ (↗13.5;1.5)
OTU02234 Sebacina sp	SH1463121.08FU	36.7	pH (↗34.5;**16.6**), forest (↗12.8;-), *Quercus*% (↗10.0;2.0)

[1]symbols in continuous predictors: ↗, near-linear increase; ↘, near-linear decline; ʃ, sigmoid increase; Γ, cumulative increase; ∩, unimodal

Table S10. Niche analysis of 50 most frequent non-EcM fungal OTUs (dataset#3). All effects are significant (P<0.001).

OTU ID and identification	UNITE Species Hypothesis	MSE_{total} (random forest)	Predictors (directionality[1], MSE of random forest; variation explained based on GLM, %)
OTU00001 Mortierella sp	SH1456793.08FU	69.3	pH (∩78.6;**69.7**), δ^{15}N (↘42.5;7.8), *Picea*% (Γ 25.3;3.6), Ca (∩20.3;2.3), time (13.2;0.7)
OTU00004 Solicoccozyma terricola	SH1491809.08FU	61.6	pH (∩61.6;**62.3**), *Picea*% (Γ 28.3;6.4), Ca (∩28.0;4.6), δ^{15}N (↘21.8;3.1), C/N (↘18.3;0.6)
OTU00005 Umbelopsis sp	SH1384653.08FU	62.9	pH (↘45.3;**52.5**), Mg (↘29.7;15.5), C/N (∩23.5;4.0), δ^{15}N (↘20.6;-)
OTU00006 Saitozyma podzolica	SH1422455.08FU	45.7	pH (∩37.8;**28.8**), P (∩29.1;11.1), *Betula*% (Γ 26.5;8.1), δ^{15}N (∩25.1;5.7), *Picea*% (↗18.1;2.8)
OTU00012 Mortierella sp	SH1456793.08FU	45.3	pH (Γ 40.8;**34.6**), C/N (↘36.2;-), δ^{15}N (Γ 29.2;6.3)
OTU00014 Mortierella sp	SH1415237.08FU	32.1	C/N (↘30.8;0.9), δ^{15}N (↗25.8;**19.7**), pH (↗15.2;1.9), EcM plant% (↘14.5;1.4), C/P (↘13.7;1.6)
OTU00032 Mortierella sp	SH1415237.08FU	50.3	pH (∩51.4;**26.7**), C/N (↘40.7;4.7), C (∩26.6;4.4), Ca (∩23.6;6.6)
OTU00034 Apodus sp	SH1433123.08FU	37.6	pH (Γ 45.2;**24.4**), *Picea*% (↘24.0;0.6), C/N (↘19.0;2.3)
OTU00036 Mortierellaceae	SH1390676.08FU	42.0	pH (∩41.3;**23.5**), C/N (↘21.9;4.3), δ^{15}N (∩19.9;1.9), *Picea*% (Γ 17.7;2.6), Ca (∩16.8;2.5)
OTU00047 Trichocladium sp	SH1488764.08FU	19.7	C/N (↘23.8;4.9), *Picea*% (↗19.9;**6.5**), pH (∩14.9;1.2), Mg (∩14.9;1.7)
OTU00050 Ascobolus sp	SH1501145.08FU	42.9	pH (↗52.3;**32.1**), Ca (↗19.7;-)
OTU00051 Geminibasidium sp	SH1420343.08FU	31.3	P (↘30.6;**11.6**), pH (Γ 19.3;3.5), *Corylus*% (↗18.7;2.5)
OTU00063 Mortierella gamsii	SH1415237.08FU	32.1	pH (∩28.3;**14.2**), C/N (↘19.6;1.5), time (19.0;2.6), tMEM05 (16.2;3.3)
OTU00065 Ilyonectria sp	SH1406223.08FU	18.9	C/N (↘30.3;1.1), pH (∩24.3;**10.4**), age (↗13.5;2.0), tMEM03 (13.3;0.8), tMEM04 (12.3;0.7), *Pinus*% (∩12.2;1.3)
OTU00066 Exophiala equina	SH1421394.08FU	26.1	pH (Γ 37.9;**13.6**), *Padus*% (↗14.4;6.9), cropland (↗12.5;6.7)
OTU00074 Ganoderma applanatum	SH1414035.08FU	24.8	*Tilia*% (↗16.7;**5.3**), *Fraxinus*% (↗10.1;3.8)
OTU00078 Mortierella sp	SH1415239.08FU	55.6	pH (↗55.7;**42.6**), Ca (↗35.1;2.6), δ^{15}N (Γ 22.4;2.2), C/N (↘18.6;0.4)
OTU00080 Mortierella sp	SH1415237.08FU	30.5	C/N (↗40.1;2.2), pH (∩32.0;**11.4**), δ^{15}N (∩17.1;3.3), *Quercus*% (↗13.6;2.5)
OTU00084 Helotiales	SH1423055.08FU	39.9	pH (∩51.7;**25.6**), C/N (↘36.6;2.5), *Quercus*% (∩18.4;4.9), Ca (∩16.2;3.3)
OTU00092 Geomyces auratus	SH1415373.08FU	40.4	age (Γ 35.9;5.3), pH (↘34.1;**21.1**), *Picea*% (Γ 32.3;5.8), δ^{15}N (↘16.0;-), *Pinus*% (∩14.4;4.8)
OTU00097 Cadophora sp	SH1405811.08FU	26.6	pH (Γ 36.2;**14.9**), C/N (↘25.1;-), AM-conifer% (↗13.5;2.2)
OTU00098 Leucosporidiales	SH1385079.08FU	41.6	*Pinus*% (↗32.4;5.6), δ15N (↘24.9;-), pH (↘23.9;**24.9**), K (↘19.2;6.9)
OTU00103 Humicolopsis cephalosporioides	SH1385231.08FU	37.0	P (↘24.7;7.0), δ^{15}N (∩23.9;2.1), pH (↘19.7;**20.0**), Mg (↘17.5;2.1)
OTU00120 Phialocephala fortinii	SH1490039.08FU	38.7	*Picea*% (↗31.7;7.3), pH (↘26.2;**25.4**), δ^{15}N (↘22.5;), K (↘17.1;3.0)
OTU00130 Dactylonectria hordeicola	SH1458599.08FU	52.4	pH (↗52.1;**46.1**), δ^{15}N (↗23.4;6.0), C/N (↘21.9;-), EcM plant% (↘15.5;2.8)

OTU00142 Hyaloscyphaceae	SH1385236.08FU	58.9	C/N (∩31.5;4.2), *Pinus*% (↗28.9;12.9), *Betula*% (Γ27.8;2.2), pH (↘20.8;**44.7**), C (↗19.1;6.5), $\delta^{15}N$ (↘16.8;-)
OTU00145 Preussia sp	SH1375028.08FU	46.8	pH (↗42.9;**35.4**), C/N (↘27.7;0.8), EcM plant% (↘19.4;-), $\delta^{15}N$ (↗19.2;4.2), *Acer*% (↗17.3;4.2)
OTU00148 Mortierella sp	SH1415243.08FU	26.5	pH (∩28.1;**13.4**), Ca (∩18.3;1.2), C/N (↘13.5;0.8)
OTU00151 Mycoarthris	SH1385243.08FU	28.0	*Populus tremula*% (↗34.9;**12.3**), plant richness (↗16.3;3.0), pH (Γ12.6;2.1)
OTU00155 Apiotrichum dulcitum	SH1464364.08FU	21.2	C/N (↘32.7;1.1), pH (↗23.5;**12.2**), EcM plant% (↘18.2;)
OTU00160 Pseudogymnoascus pannorum	SH1415371.08FU	21.4	age (Γ13.4;1.2), *Picea*% (↗10.0;**3.5**), pH (∩9.9;2.4)
OTU00173 Mortierella alpina	SH1492736.08FU	16.9	pH (Γ17.9;**2.8**), ntMEM3 (15.4;2.1), Ca (↗15.3;2.2)
OTU00197 Apiotrichum sp	SH1464365.08FU	16.7	pH (∩26.9;**9.5**), tMEM02 (15.7;3.7), *Picea*% (Γ 13.7;0.8), tMEM05 (10.1;1.5)
OTU00211 Hyaloscyphaceae	SH1385373.08FU	25.5	*Betula*% (Γ22.3;4.8), N/P (↗15.4;**9.2**), P (∩15.0;0.9)
OTU00213 Penicillium spinulosum	SH1391391.08FU	44.0	*Betula*% (↗30.8;6.6), time (28.2;10.6), C/N (∩26.7;2.7), pH (↘21.6;3.4), *Pinus*% (↗16.3;**21.4**)
OTU00246 Mortierella pseudozygospora	SH1390676.08FU	7.5	*Picea*% (↗12.8;**2.3**), tMEM20 (10.7;-)
OTU00252 Metarhizium sp	SH1411349.08FU	25.2	pH (∩24.1;**12.5**), Ca (↗19.3;5.0), time (15.6;4.5), patch size (∩14.8;-)
OTU00298 Neonectria candida	SH1406222.08FU	47.5	C/N (↘31.9;0.9), pH (↗31.1;**28.3**), *Acer*% (↗20.1;7.0)
OTU00312 Hysteriaceae	SH1406793.08FU	48.6	park (↗21.8;**26.1**), $\delta^{15}N$ (↗15.9;7.2), forest (↘15.6;11.2), C/N (↘15.0;3.0), pH (↗10.0;4.8)
OTU00316 Operculomyces laminatus	SH1399519.08FU	39.5	pH (↗38.6;**25.1**), $\delta^{15}N$ (↗18.5;2.2), EcM plant% (↘16.3;2.3)
OTU00330 Tolypocladium sp	SH1438847.08FU	22.7	*Padus*% (↗18.7;**13.3**), *Salix*% (↗16.7;4.7), *Corylus*% (↗14.6;3.6), pH (↗10.1;2.1)
OTU00421 Alatospora	SH1405852.08FU	19.4	plant richness (↗22.2;**12.3**), pH (Γ16.0;3.4)
OTU00626 Cladosporium sp	SH1428520.08FU	28.8	forest (↘18.8;10.5), time (17.7;1.3), EcM plant% (↘16.4;1.7), park (↗13.7;3.1)
OTU00683 Chaetomium	SH1463351.08FU	27.7	C/N (↘24.7;2.2), EcM plant% (↘19.5;1.8), pH (Γ19.3;**10.0**)
OTU00712 Tetracladium sp	SH1491365.08FU	43.7	pH (↗39.2;**31.4**), C/N (↘25.8;0.5), EcM plant% (↘22.4;3.0), *Quercus*% (↗19.0;2.8)
OTU00722 Tetracladium sp	SH1491368.08FU	52.9	pH (↗45.2;**50.1**), EcM plant% (↘23.2;3.9), Ca (↗22.9;0.7), C/N (↘22.8;0.4), Amelanchier% (↗20.5;-)
OTU00767 Trichoderma viride	SH1424493.08FU	36.3	pH (↘28.5;5.0), time (18.3;6.7), *Pinus*% (↗17.9;**18.8**), C/N (Γ16.7;), tMEM03 (16.0;1.4)
OTU01015 Lasiosphaeriaceae	SH1433158.08FU	38.4	pH (↗38.2;**28.5**), Ca (↗16.1;-), EcM plant% (∩12.6;5.1)
OTU01102 Ascomycota	SH1491364.08FU	20.1	*Acer*% (Γ17.2;**10.7**), EcM plant% (∩17.2;-); pH(↗10.0;3.0)
OTU02260 Trichocladium asperum	SH1463303.08FU	39.0	*Quercus*% (↗24.6;10.5), $\delta^{15}N$ (↗23.1;4.1), pH (↗16.2;**15.2**), EcM plant% (↘15.2;3.5)

[1]symbols in continuous predictors: ↗, near-linear increase; ↘, near-linear decline; ʃ, sigmoid increase; Γ, cumulative increase; ∩, unimodal

Table S11. Effect of habitat types on richness of fungal groups and environmental parameters. All effects are significant (P<0.001; differences among habitat type levels P<0.05).

Dependent variable	Habitat type (R^2, %)	Habitat type effect[1]
Fungi	6.54	ruins=woodland>park=woodland=alley=grassland=forest>energy=cropland>>bog
Fungi (Shannon index)	3.27	ruins>woodland=grassland>park=alley=woodland=cropland=energy=forest>bog
**Fungal functional guilds**		
ectomycorrhizal fungi	10.45	woodland>forest=park=ruins=alley>woodland>bog=energy=grassland>>cropland
AM fungi	8.28	grassland>cropland=woodland>ruins=alley=energy=park=woodland>forest>bog
root endophytes	3.86	bog>>forest>grassland=energy=ruins=woodland>park=cropland=woodland=alley
dung saprotrophs	6.17	grassland>woodland=alley=ruins=park=cropland=woodland>energy>forest>bog
litter saprotrophs	3.76	ruins=grassland=woodland=alley=woodland=park=cropland>energy=forest>bog
wood saprotrophs	3.43	ruins>woodland=cropland=alley=woodland=park=grassland=energy=forest>bog
white-rot saprotrophs	8.49	ruins=forest>park=woodland=alley=woodland=bog>grassland=cropland=energy
**Fungal taxonomic groups**		
Aphelidiomycota	8.59	energy>woodland=cropland=alley=grassland=ruins=park>woodland=forest=bog
Pleosporales	10.26	energy=cropland=grassland>woodland=alley=woodland=ruins=park>forest=bog
Onygenales	7.35	woodland=cropland=grassland=woodland>alley=park=ruins=energy>forest=bog
Geoglossales	11.20	woodland=grassland=woodland>ruins=park=alley=bog=cropland=energy=forest
Helotiales	9.06	ruins>forest=bog=woodland=park>woodland=alley=grassland>energy=cropland
Thelebolales	3.61	ruins=park=alley>woodland=forest=woodland=cropland=grassland>energy>bog
Pezizales	7.22	alley=ruins=park=woodland=woodland>grassland=forest=energy=cropland>bog
Glomerellales	3.85	cropland=ruins=alley>park=woodland=energy=woodland=grassland>forest=bog
Hypocreales	3.99	cropland=grassland>woodland=ruins=alley=park=woodland>forest=energy>bog
Microascales	10.28	cropland>grassland=woodland=alley>ruins=park=woodland=energy>forest=bog
Sordariales	7.79	grassland=cropland>woodland=alley=ruins=woodland=park>energy=forest>bog
Xylariales	4.19	ruins>park=energy=cropland>alley=woodland=woodland=grassland=forest=bog
Agaricales (NM)	8.22	woodland=ruins=grassland>alley=woodland=forest=park=energy=cropland>bog
Auriculariales	3.70	grassland>ruins=woodland=forest=alley=park=woodland=cropland=bog>energy
Ceratobasidiaceae	4.50	grassland>park=ruins=woodland=alley=cropland=woodland=energy>forest=bog
Hymenochaetales	4.49	forest>energy=ruins=park=woodland=bog=alley=cropland=grassland=woodland
Sebacinales (NM)	5.61	energy=bog>grassland=ruins=cropland=forest=woodland=woodland=park=alley
Trechisporales	4.06	forest>bog=woodland=ruins=park=cropland=grassland=alley=woodland=energy
Spizellomycetes	5.71	cropland>energy=grassland=ruins=woodland=woodland=park=alley=forest>bog
Diversisporales	5.88	energy=cropland=grassland>ruins=alley=bog=woodland=park=woodland=forest
Glomerales	8.46	grassland>woodland=cropland=ruins=alley=park=energy=woodland>forest=bog
Monoblepharomycota	9.34	energy>cropland>grassland=bog=ruins=forest=woodland=park=alley=woodland
Neocallimastigomycota	10.22	energy>grassland=woodland=cropland=alley=ruins=park=forest=woodland=bog
Rozellomycota	4.90	energy=woodland>alley=ruins=forest=woodland=park=grassland>cropland=bog
Zoopagomycota	6.17	energy=cropland=grassland>forest=woodland=park=bog=woodland=ruins=alley
unknown fungi	13.33	energy>cropland=grassland=woodland=ruins=alley>park=woodland=bog>forest
**Lineages of EcM fungi**		
lineage richness	2.46	ruins=forest=woodland=park=alley=woodland>energy
/amanita	7.47	forest>woodland>ruins=woodland=park=energy>alley
/amphinema-tylospora	19.62	forest>ruins=woodland=park>woodland>energy>alley
/cantharellus	3.99	forest>woodland=ruins=alley=energy=woodland=park
/cenococcum	5.47	woodland=forest>park=alley=ruins>woodland>energy
/clavulina	5.03	forest>woodland=ruins=park=alley>energy>woodland
/cortinarius	7.64	forest=energy=woodland>ruins=woodland>park=alley
/galactinia	14.63	park=alley=woodland>ruins=woodland>energy=forest
/genea-humaria	4.67	woodland=alley=ruins=park>forest>woodland>energy
/geopora	9.06	alley=woodland=energy>park=ruins=woodland=forest
/hebeloma-alnicola	12.48	energy>ruins=alley=woodland=woodland=park>forest
/hygrophorus	6.73	forest>ruins=woodland=alley=park>woodland=energy
/inocybe	8.68	park=ruins=woodland=alley>forest=woodland>energy
/laccaria	3.00	energy>ruins>forest=park=woodland=alley>woodland
/meliniomyces	11.69	energy>forest>woodland=ruins=woodland>park=alley
/otidea	3.33	woodland=ruins=park=alley=forest>woodland>energy

/piloderma	10.90	forest>ruins=woodland=park=woodland=alley>energy
/pisolithus-scleroderma	13.28	park=ruins=alley>woodland=woodland=forest>energy
/pseudotomentella	5.45	forest>woodland=ruins=woodland=park=alley>energy
/pulvinula	4.22	energy=woodland=alley=ruins>park=forest=woodland
/rhodoscypha	4.03	forest=woodland>park=ruins=woodland=alley=energy
/russula-lactarius	12.93	forest>woodland=ruins=park=energy=woodland=alley
/sebacina	3.26	woodland>park=forest=alley=ruins=woodland>energy
/sphaerosporella	10.57	alley=woodland=park=woodland>ruins=forest>energy
/terfezia-peziza depressa	9.78	alley=park=woodland=energy>ruins=forest=woodland
/tomentella-thelephora	4.22	woodland>ruins=park=forest=alley>woodland=energy
/tricholoma	3.47	woodland>ruins=forest=park=alley=woodland>energy
/tuber-helvella	19.46	alley=park=woodland>ruins=woodland>energy=forest
/wilcoxina	6.63	forest>ruins=woodland=park=woodland=alley=energy
uncertain2	7.83	forest>woodland=ruins=woodland=park=energy=alley
**Soil properties and floristic variables**		
soil $\delta^{15}N$	35.35	cropland>alley=woodland=energy=grassland=park=village>woodland>forest>bog
soil C/N ratio	24.13	bog=cropland>forest=woodland=ruins=alley=park=woodland>energy>grassland
soil C/P ratio	32.13	bog>forest=woodland>grassland=cropland=ruins=park=alley=woodland>energy
soil N/P ratio	29.47	woodland=forest=bog=grassland>ruins=park=alley=woodland>cropland>energy
soil pH	27.96	woodland=alley=grassland=park=ruins=cropland=woodland>energy>forest>bog
woody plant richness	18.06	ruins>woodland=woodland=forest=park=alley=energy>bog>grassland=cropland
EcM plant richness	21.33	ruins=forest=energy=woodland=park>woodland=alley=bog>grassland=cropland
EcM plant%	31.95	bog=energy>forest>woodland=park=alley=woodland>ruins>grassland=cropland
vegetation age	25.23	ruins=park=woodland>bog=forest=alley>grassland=woodland>energy>cropland
Populus x wettsteinii%	90.00	energy=woodland=forest=bog=grassland=ruins=park=alley=woodland=cropland
Acer platanoides%	20.14	ruins=park=alley>woodland>forest=woodland>energy>bog=grassland=cropland
Picea abies%	19.68	forest>ruins=park=energy=woodland>bog=woodland>alley=cropland>grassland
Tilia spp.%	19.12	park=ruins>alley=forest=woodland=woodland>energy>bog=grassland=cropland
Quercus robur%	16.51	woodland=park=ruins>grassland=alley=forest>energy=woodland>bog=cropland
Fraxinus excelsior%	14.94	ruins>park=woodland=alley=woodland>forest>energy>bog=grassland=cropland
Pinus sylvestris%	10.84	bog>forest>woodland=woodland=park=ruins>energy>alley=grassland=cropland
Ulmus glabra%	10.43	ruins=park=alley=woodland>forest=woodland=energy>bog>grassland>cropland

[1]>/=/< indicate significant differences among groups

Table S12. Effect of anthropogenic impacts on soil properties, vegetation and richness of fungal groups in woody plant-dominated habitats. All effects are significant (P<0.001; differences among habitat type levels P<0.05).

Dependent variable	Biome effect (R^2, %)	Differences among biomes (ranked order)[1]
Fungi richness	4.05	V>W=U
Fungi Shannon index	3.13	V=U>W
**Fungal functional groups**		
arbuscular mycorrhizal fungi	9.99	V>U>W
root endophytes	5.07	W>V=U
dung saprotrophs	19.03	V=U>W
litter saprotrophs	9.37	V>U>W
wood saprotrophs	3.00	V>U=W
leaf pathogens	7.51	V=U>W
animal parasites	4.34	U=V>W
opportunistic human pathogens	3.77	V=U>W
**Fungal taxonomic groups**		
Aphelidiomycota	3.41	V=U>W
Pleosporales	13.31	U=V>W
Onygenales	7.38	U=V>W
Pezizales	14.41	V=U>W
Glomerellales	10.87	V=U>W
Hypocreales	9.36	V=U>W
Microascales	12.94	V=U>W
Sordariales	15.64	V=U>W
Xylariales	7.74	V=U>W
Certobasidiaceae	10.77	V=U>W
Hymenochaetales	3.54	W>V=U
Trechisporales	4.68	W>U=V
Cystofilobasidiales	7.57	U=V>W
Rhizophlyctidomycetes	11.43	V>U>W
Spizellomycetes	3.75	V>U=W
Glomerales	11.18	V>U>W
Endogonomycetes	3.22	W>V=U
Umbelopsidomycetes	12.43	W>U=V
unknown fungi	4.45	U=V>W
**Lineages of EcM fungi**		
/amanita	5.13	W>U=V
/amphinema-tylospora	11.8	W>V=U
/cantharellus	3.12	W>U=V
/cenococcum	3.41	W>U=V
/cortinarius	6.31	W>U=V
/galactinia	10.42	U=V>W
/geopora	4.31	U>V>W
/hebeloma-alnicola	3.89	V>U>W
/hygrophorus	4.56	W>U=V
/inocybe	5.81	V>U>W
/meliniomyces	8.72	W>U=V
/piloderma	5.94	W>U=V
/pisolithus-scleroderma	10.52	V=U>W
/russula-lactarius	8.69	W>>U=V
/sphaerosporella	5.82	V=U>W
/terfezia-peziza depressa	6.78	U>V>W
/tuber-helvella	14.52	U=V>W
/wilcoxina	3.82	W>V=U
uncertain2	4.24	W>U=V
**Edaphic and floristic variables**		
soil $\delta^{15}N$	23.07	V>U>>W
soil C/N ratio	7.71	W>U=V

soil C/P ratio	21.42	W>U=V
soil N/P ratio	18.43	W>U=V
soil pH	16.81	V=U>W
tree richness	4.36	V>W=U
EcM plant%	10.71	W=U>V
Tilia spp.%	15.44	V>U>W
Fraxinus excelsior%	11.32	V>W=U
Picea abies%	10.31	W>V>U
Quercus robur%	9.08	V>U=W
Pinus sylvestris%	3.75	W>U=V

[1]abbreviations: U, urban; V, village; W; wild forested biomes combined; >/=/< indicate significant differences among groups

Table S13. Effect of island habitat and its type on richness of fungi and environmental parameters. Values in bold indicate significant relationships (P<0.001).

Dependent variables	Island type (R^2, %)[1]	Differences among island types[2]	Patch size[3] (direction; R^2, %)	Island type x patch size (R^2, %)	Isolation distance[3] (direction; R^2, %)	Type x isolation distance (R^2, %)
Fungi richness	2.89	F>B=F>N	↘1.78	1.04	**↘6.19**	2.40
Fungi Shannon index	2.68	F>A=B>N	↘1.49	ns	**↘7.82**	2.60
**Functional guilds**						
arbuscular mycorrhizal	1.38	F>A>N>B	↘2.55	1.91	ns	ns
root endophytes	2.88	B=N>A=F	↗3.06	ns	ns	ns
saprotrophs (all)	**4.44**	F>>A>N=B	**↘4.35**	ns	↘3.35	1.55
dung saprotrophs	**5.98**	F>A=N=B	**↘8.34**	ns	ns	ns
litter saprotrophs	**4.94**	F>A=N=B	**↘5.82**	ns	**↘4.35**	2.14
wood saprotrophs	2.48	F>A>N=B	↘1.69	ns	↘3.06	2.16
leaf pathogens	**5.73**	F>>A>B=N	**↘4.57**	1.05	ns	ns
animal parasites	**6.38**	F>A>N=B	**↘4.92**	ns	ns	ns
opportunistic human	**5.33**	F=A>B=N	↘2.43	ns	ns	ns
dimorphic yeasts	3.63	F=A>B=N	1.32	ns	ns	ns
**Fungal taxonomic**						
Aphelidiomycota	**3.98**	F>A=N=B	↘3.73	1.37	ns	ns
Pleosporales	**12.36**	F>>A>N=B	**↘9.99**	1.84	ns	ns
Capnodiales	ns	ns	ns	ns	↘3.46	**5.24**
Onygenales	**7.87**	F=A>N=B	↘2.56	ns	ns	ns
Pezizales	**8.25**	F>A>N=B	**↘4.30**	ns	ns	ns
Saccharomycetales	3.73	B>N=A>F	↘1.35	ns	ns	ns
Coniochaetales	3.70	B=N=F>A	ns	ns	ns	ns
Glomerellales	**4.70**	F>A=N=B	**↘8.06**	1.35	ns	ns
Hypocreales	**7.71**	F>A>N=B	↘6.19	ns	ns	ns
Microascales	**8.24**	F>A=N=B	↘6.99	ns	ns	ns
Myrmecridiales	2.65	F=A>N=B	ns	ns	ns	ns
Sordariales	**9.08**	F>>A>N=B	**↘9.27**	1.32	ns	ns
Xylariales	3.40	F>A=N=B	↘2.81	1.38	**↘4.48**	3.66
Ceratobasidiaceae	**5.98**	F>A=N=B	↘3.94	ns	ns	ns
Hymenochaetales	1.83	B=N=A>F	**↗3.47**	ns	ns	ns
Cystofilobasidiales	**4.19**	F>A=N=B	↘2.10	1.06	ns	ns
Filobasidiales	2.41	F>A=N=B	↘2.98	ns	ns	ns
Rhizophlyctidomycetes	2.56	F>A=N>B	↘3.69	ns	ns	ns
Glomerales	1.43	F>A=N>B	↘3.33	2.02	ns	ns
Endogonomycetes	3.18	B=N>F=A	↗1.22	ns	ns	ns
Umbelopsidomycetes	**6.19**	B>N>A=F	**↗4.63**	ns	ns	ns
**EcM fungal lineages**						
/amanita	**4.43**	B=N>A=F	↗2.97	ns	ns	ns
/amphinema-tylospora	**8.43**	B=N>A>F	↗1.95	ns	ns	ns
/cantharellus	3.06	B>N=A=F	↗1.49	ns	ns	ns
/cenococcum	ns		↗3.25	ns	ns	ns
/clavulina	ns		↗2.99	ns	ns	ns
/galactinia	**4.76**	A=F>B=N	ns	ns	ns	ns
/genea-humaria	**5.24**	F=A>B=N	ns	ns	↘3.10	**8.93**
/geopora	**4.08**	F=A>B=N	↘2.36	ns	ns	ns

/hebeloma-alnicola	**4.98**	F=A>N=B	↘2.91	1.23	ns	ns
/inocybe	2.29	F=A>N=B	↘0.43	1.75	ns	ns
/otidea	1.78	F=B>A=N	↘2.29	ns	ns	ns
/piloderma	**4.78**	B>N>A=F	↗**4.28**	ns	ns	ns
/pisolithus-scleroderma	ns		↘**4.73**	ns	ns	ns
/russula-lactarius	**8.10**	N=B=A>F	↗2.28	1.24	ns	ns
/sebacina	ns	ns	ns	ns	↘2.18	3.07
/sphaerosporella	**4.01**	A=F>N=B	ns	ns	ns	ns
/terfezia-peziza depressa	3.69	A>F=B=N	ns	ns	ns	ns
/tomentella-thelephora	2.21	F=A>N=B	↘1.53	1.66	↘**5.54**	ns
/tomentellopsis	3.14	B>A=N=F	ns	ns	ns	ns
/tuber-helvella	**5.68**	F>A>N=B	↘3.45	1.50	ns	ns
/wilcoxina	2.45	B=N>A=F	ns	ns	ns	ns
**Environmental variables**						
Soil pH	**7.66**	F=A>N=B	↘**4.50**	1.40	ns	ns
Soil C/P	3.42	B=A=N>F	↗**9.76**	ns	ns	ns
Soil δ^{15}N	**8.50**	F=A>N>B	↘**10.30**	ns	ns	ns
Picea abies%	**7.89**	B=N>A=F	ns	ns	ns	ns
Salix spp.%	**4.34**	F>A>N>B	↘**5.98**	ns	ns	ns

[1]ns, not suggestive at P>0.05.

[2]abbreviations: A, aquatic islands; B, bog islands; F; field islands; N, no island = control; >/=/< indicate significant differences among groups;

[3]symbols in continuous predictors: ↗, near-linear increase; ↘, near-linear decline

Table S14. The effect of age and virgin habitat for old forests (>99 years). Values in bold are significant (P<0.001).

	Virgin vs. non-virgin (R^2, %)[1]	Virgin vs. non-virgin effect[2]	Age effect (R^2, %)[3]
**Fungal taxonomic and functional groups**			
Fungi richness	ns	ns	ns
white-rot decomposers	**3.0**	V>NV	↗1.6
Capnodiales	**5.6**	V>NV	↗1.8
Hymenochaetales	**2.7**	V>NV	ns
Polyporales	**2.9**	V>NV	ns
Trechisporales	**3.2**	V>NV	ns
Agaricostilbomycetes	**2.2**	NV>V	ns
**Lineages of EcM fungi**			
amphinema-tylospora	**6.5**	V>NV	ns
cortinarius	**2.6**	V>NV	ns
elaphomyces	**3.7**	V>NV	ns
meliniomyces	ns	ns	↘**3.0**
piloderma	**4.3**	V>NV	ns
pisolithus-scleroderma	ns	ns	↗**2.2**
**Edaphic and floristic variables**			
Soil C	**4.7**	V>NV	ns
Soil $\delta^{15}N$	**4.9**	NV>V	ns
Soil phosphorus	**2.4**	V>NV	ns
Soil potassium	**5.9**	V>NV	ns
Quercus robur%	**4.4**	NV>V	↗**6.3**
Picea abies%	**4.3**	V>NV	ns
Pyroleae spp.%	**4.4**	V>NV	ns

[1]ns, not suggestive at P>0.05;

[2]abbreviations: V, virgin forest; NV, non-virgin forest; WP, wooded pasture; >/=/< indicate significant differences among groups;

[3]abbreviations: C, coppiced; M, managed; UM, unmanaged; >/=/< indicate significant differences among groups

Table S15. The effect of selective harvesting and time since harvesting on fungal richness and environmental variables. Values in bold are significant (P<0.001).

Dependent variable	Harvesting effect (direction; R^2, %)[1]	Time since harvesting (direction; R^2, %)
Fungi richness	**↘4.0**	1.5
Fungi Shannon index	**↘4.5**	ns
**Fungal functional guilds and life forms**		
ectomycorrhizal fungi	**↘1.7**	ns
white-rot saprotrophs	ns	**↘11.4**
mycoparasites	**↘2.1**	ns
animal parasites	**↘2.9**	ns
opportunistic human pathogens	**↘5.1**	ns
yeasts	**↘4.2**	ns
dimorphic yeasts	**↘4.9**	ns
**Fungal taxonomic groups**		
Capnodiales	**↘3.1**	ns
Venturiales	**↘2.2**	ns
Chaetothyriales	**↘3.1**	ns
Eurotiales	**↘3.7**	ns
Helotiales	**↘3.9**	ns
Glomerellales	**↘1.8**	ns
Hypocreales	**↘2.5**	ns
Auriculariales	**↘2.6**	ns
Sebacinales (non-EcM)	**↘2.2**	ns
**Lineages of EcM fungi**		
amphinema-tylospora	**↗2.4**	ns
cortinarius	**↘5.0**	**↗5.0**
hygrophorus	ns	**↗5.5**
inocybe	ns	**↘5.6**
pisolithus-scleroderma	ns	**↘4.3**
pseudotomentella	**↗2.7**	ns
russula-lactarius	**↗3.6**	ns
tricholoma	**↘2.4**	ns
tuber-helvella	ns	**↘3.5**
wilcoxina	**↗2.9**	ns
**Soil properties and vegetation**		
soil carbon	**↘8.7**	ns
soil magnesium	**↘4.3**	ns
plant richness	ns	**↘3.6**
Quercus robur%	ns	**↗5.7**
Picea abies%	**↗5.7**	**↗6.7**
Pinus sylvestris%	**↘2.6**	ns
Sorbus aucuparia%	**↗2.1**	ns

[1]symbols in continuous predictors: ↗, near-linear increase; ↘, near-linear decline; ns, not suggestive at P>0.05
